# Trail Pheromone Does Not Modulate Subjective Reward Evaluation in *Lasius niger* Ants

**DOI:** 10.3389/fpsyg.2020.555576

**Published:** 2020-09-23

**Authors:** Felix B. Oberhauser, Stephanie Wendt, Tomer J. Czaczkes

**Affiliations:** ^1^Animal Comparative Economics Laboratory, University of Regensburg, Regensburg, Germany; ^2^Centre for the Advanced Study of Collective Behaviour, University of Konstanz, Konstanz, Germany

**Keywords:** social information, value perception, preference, conformity, recruitment, pheromone trails

## Abstract

Comparing the value of options is at the heart of economic decision-making. While an option may have an absolute quality (e.g. a food source has a fixed energy content), the perceived value of the option may be malleable. The factors affecting the perceived value of an option may thus strongly influence which option is ultimately chosen. Expectations have been shown to be a strong driver of perceived value in both humans and social insects, causing an undervaluation of a given option if a better option was expected, and an overvaluation if a poorer one was expected. In humans, perceived value can be strongly affected by social information. Value perception in some insects has also been shown to be affected by social information, showing conformism as in humans and other animals. Here, over a series of experiments, we tested whether pheromone trail presence, a social information source, influenced the perceived value of a food source in the ant *Lasius niger*. We found that the presence of pheromone trails leading to a sucrose solution does not influence food acceptance, pheromone deposition when returning from a food source, drinking time, or frequency of U-turns on return from the food. Two further assays for measuring changes in food acceptance, designed to increase sensitivity by avoiding ceiling effects, also showed no effect of pheromone presence on food acceptance. In a separate study, *L. niger* have also been found to show no preference for, or avoidance of, odors associated with foods found in the presence of pheromone. We are thus confident that trail pheromone presence does not affect the perceived value of a food source in these ants.

## Introduction

Understanding the mechanisms behind decision-making is key to understanding animal behavior. Decision-making systems lie on a trade-off continuum between cheap (in terms of processing power or time) but inaccurate systems and costly but more reliable ones. Perhaps the cheapest but least accurate is random choice. Somewhat costlier, but much more effective, is the use of heuristics (‘rules of thumb’), such as “do what I did last time,” “do what is easiest,” or “do what others are doing.” Finally, the value of each decision outcome can be estimated and compared to other available options, often resulting in a choice for the option reaping the largest total reward. This type of decision-making is the basis of the majority of human economic decision-making models, such as the Expected Utility Theory (Mankiw, 2011), as well as of classical optimality-based models of animal behavior (Krebs et al., 1978; Stephens and Krebs, 1986). In order to compare the value of options, however, one must first assign a value to each option. Value perception thus has a large influence on decision-making.

The perceived value of options can be influenced by various things such as aspects of the option itself, or attributes of the decision maker. Moreover, the influences on perceived value may be absolutely rational, boundedly or ecologically rational, or completely irrational. Examples of completely rational influences on perceived value include time discounting (Frederick et al., 2002; Craft, 2016; Hayden, 2016), although often such time-discounting is too strong to be considered fully rational (Hillemann et al., 2014; Hayden, 2016).

However, perceived value can be influenced irrationally as well. For example, it has been argued that risk aversion when risking gains, and risk-seeking when risking losses, can emerge directly from the non-linear nature of perception and learning (Kacelnik and El Mouden, 2013), resulting in irrational behavior (but see Lim et al., 2015). Similar mechanisms can also result in a preference for rewards experienced when in a low state (e.g. hungry or stressed) over otherwise identical rewards experienced when in a high state (Schuck-Paim et al., 2004; Pompilio et al., 2006; Aw et al., 2011), or a preference for rewards associated with harder work over easier-to-access rewards (Kacelnik and Marsh, 2002; Czaczkes et al., 2018).

Finally, value attribution can itself be carried out by following heuristics, which allow more rapid valuation at the risk of making errors. This is often termed ‘bounded rationality’ (Simon, 1990) or ‘ecological rationality’ (Todd and Gigerenzer, 2012), as they often result in optimal behavior given the resource and information limits humans and animals find themselves under. For example, valuation can be performed in a relative manner: rather than assign independent valuations to each option, options can be compared and ranked along one or a few criteria. This can cause the perceived value of an option to rise or fall, depending on the reference point (Kahneman and Tversky, 1979; Flaherty, 1996). Such relative value perception is common in humans (Camerer, 2004), and has been described in animals, including rats, honeybees, and ants (Bitterman, 1976; Flaherty, 1996; Wendt et al., 2019). Relative valuation can result in irrational behavior such as taking into account irrelevant alternatives, resulting in decoy effects which have been reported in humans, other vertebrates, insects, and even slime-molds (Huber et al., 1982; Shafir et al., 2002; Latty and Beekman, 2011; Sasaki and Pratt, 2011). Another important influence on perceived value, which can be boundedly rational, is social information: how others evaluate the option may affect one’s own evaluation.

Social information has a very large impact on many aspects of decision-making. Strategic information use – whether to rely on privately acquired information or social information – has received extensive attention by researchers (Coolen et al., 2003; Dall et al., 2005; Kendal et al., 2005, 2009; Leadbeater and Chittka, 2007). Social information use allows the costs of information collection to be avoided (Valone and Templeton, 2002). However, social information may be less nuanced than privately collected information, for example lacking the full suite of sensory dimensions offered by privately acquired information (Czaczkes et al., 2019). Social information is also open to dishonest signaling from competitors (Bugnyar and Heinrich, 2006), and carries its own costs, such as time invested in acquiring and providing social information (Dechaume-Moncharmont et al., 2005; Grüter and Leadbeater, 2014). Nonetheless, social information use, and the copying of others, is extremely common in both humans and non-human animals (Rendell et al., 2010). In humans, conformity can result in people changing their evaluation of item quality depending on the evaluation of others (Pincus and Waters, 1977; Bone, 1995; Jayles et al., 2017). By analogy, animals may also change their evaluation of a resource according to the revealed evaluation of others. Indeed, inadvertent social information – the observation of the behavior of others – has been shown to strongly influence preference, for example during food selection in rats (Jolles et al., 2011) and bees (Avarguès-Weber et al., 2018) or mate selection in fruit-flies (Mery et al., 2009; Danchin et al., 2018). For social insects, social information is relied on to make collective decisions, as usually a collective decision must be reached without all individuals having direct experience of all the options, or even more than one option (Robinson et al., 2009). Pheromone trails are an important source of social information for many ants. They not only signal resource location, but correlate (albeit very roughly) with resource quality (Beckers et al., 1992; Detrain and Prieur, 2014; Wendt et al., 2019), and act as an important source of reassurance to experienced foragers, allowing them to run faster and straighter (Czaczkes et al., 2011). Pheromone deposition, in turn, can reflect the certainty of an ant’s memory, and whether its environment has changed (Czaczkes and Heinze, 2015).

Several studies have shown an effect of intentional social information (signals) on subjective evaluation in insects. A study on the stingless bee *Melipona quadrifasciata*, showed that thoracic vibrations from the donor during trophallaxis increase with increasing food quality, and result in improved associative learning in the receiver (Mc Cabe et al., 2015). This effect may also work via modulating perceived value, but may alternatively function via other supports to learning, such as increasing the receiver’s attention. More conclusively, Baracchi et al. (2017) demonstrated that aversive pheromones can reduce the appetitive response in honeybees to sucrose, while attractive pheromones, usually used to signal the nest entrance, can increase appetitive responses. Rossi et al. (2018) demonstrated the converse pattern for aversive stimuli, which were enhanced by aversive pheromones and reduced by attractive ones. Even sex pheromones appear to positively modulate the response to food rewards in moths and honeybees (Hostachy et al., 2019; Baracchi et al., 2020). Finally, and very relevant for the current study, Rossi et al. (2020) conducted a study in parallel to ours, and asking an identical question to that investigated here. They found a positive effect of pheromone trails on resource evaluation in the Argentine ant, *Linepithema humile.*

The ant, *Lasius niger*, is an emerging insect model in the study of value perception and social information use (Grüter et al., 2011; Czaczkes et al., 2019; Wendt et al., 2019). Here, we set out to test whether *L. niger* ants are influenced by social signals when evaluating food quality. Resource evaluation by *L. niger* is distorted by a range of non-social effects, such as by comparison against other resources (Wendt et al., 2019), by shared associations with previously evaluated food (Wendt and Czaczkes, 2020), and by the effort invested in obtaining a reward (Czaczkes et al., 2018). Inadvertent social information also affects resource evaluation, with ants undervaluing resources consumed in the presence of other ants, and preferring odors associated with solitary feeding over those associated with group feeding in a binary choice assay (Wendt et al., 2020). As in many other ants, *L. niger* deposit pheromone trails to food sources, and deposit more pheromone to resources they perceive as being of higher quality (Beckers et al., 1992; Detrain and Prieur, 2014; Frizzi et al., 2018; Wendt et al., 2019). Pheromone deposition is also depressed by repeatedly encountering nestmates on the trail or while feeding (Czaczkes et al., 2013b; Wendt et al., 2020) or by trail pheromone already being present on the substrate (Czaczkes et al., 2013a). In a series of experiments, we test whether the presence of a strong pheromone trail, implying positive evaluation by nestmates, drives preference or distorts perceived value in *L. niger* foragers.

## Materials and Methods

### Animals

All experiments were conducted on queenless *Lasius niger* colony fragments consisting of ca. 2,000 workers and small amounts of male brood, which were kept in plastic foraging boxes with a layer of plaster of Paris on the bottom and a circular plaster nest (14 cm diameter, 2 cm high). The colonies were provided with 1M sucrose syrup and water *ad libitum* and were starved 4 days prior to testing. The number of ants and colonies tested in each experiment is provided in Table 1.

**TABLE 1:** Number of ants and colonies tested in each experiment – colonies are in brackets.

Solution	Trail attraction	Experiment 1	Experiment 2	Experiment 3
2 gl/ml	366 (2)	22 (6)	16 (4)	–
4 gl/ml	178 (3)	22 (6)	–	233 (11)
8 gl/ml	254 (4)	23 (6)	16 (4)	–
DCM	198 (3)	24 (6)	14 (4)	237 (11)

### Pheromone Extraction

A pheromone extract was created following a procedure modified from von Thienen et al. (2014). *L. niger* workers were killed by keeping them in a freezer for 45 min. Afterwards, pheromone was obtained by dissecting the gaster to isolate the hindgut and rupturing it in a vial containing dichloromethane (DCM) as solvent. This way, solutions of three different strengths were created: (i) strong – 8 glands per ml DCM, (ii) medium – 4 glands/ml or (iii) weak – 2 glands/ml DCM, henceforth referred to as 2, 4, and 8 gl/ml. Trails created using 10 μl of the 4-gland solution over 10 cm are the equivalent of a strong naturally formed trail (von Thienen et al., 2014). A DCM-only solution was used as control. All solutions were stored at −20°C and kept on ice during experiments. To reduce for evaporation, the vials were immediately closed after solution was taken out and the solutions were replaced after three sessions at most. The content of the solutions was unknown to the experimenter and also during video analysis (see below).

### Statistical Analysis Tools

All analyses were conducted in R version 3.6.3. Data handling and visualization were performed using the xlsx, dplyr, and ggplot2 packages (Dragulescu and Arendt, 2020; Wickham et al., 2020a, b). For statistical analyses, (generalized) linear mixed models were run using the glmmTMB package (Magnusson et al., 2020). All models were tested for fit using the DHARMa package (Hartig, 2020). Main effects were tested using the Anova command from the car package (Fox et al., 2020). To test performance against chance level of 50% and to conduct pairwise comparisons, we used the emmeans package (Lenth et al., 2020). For those comparisons, we provide the 95% confidence interval for the pairwise difference between levels for linear mixed models (where 0 corresponds to no difference), or the 95% confidence interval ratio for generalized linear mixed models (where 1 corresponds to no difference). In cases of simultaneous inference, *p*-value adjustments for multiple comparisons were conducted using the mvtnorm package (Genz et al., 2020). Please see the Electronic Supplementary Material (ESM) 1 for an analysis protocol covering all analysis steps with limited commentary and for a comprehensive list of package versions. All raw data used for analysis can be found in Electronic Supplementary Material 2.

### Concentration-Dependent Pheromone Trail Attraction

We first assessed whether the pheromone solutions were perceived by ants, i.e., evoked following behavior. To this end, we measured the attraction of three different pheromone concentrations (2, 4, or 8 gl/ml, see above) against a solvent-only (DCM) solution, as well as DCM against itself.

#### Procedure

Ants were allowed onto a Y-maze (following Czaczkes, 2018, Figure 1A) via a drawbridge. One of the 10 cm long arms presented a disposable paper overlay with a trail of DCM created by applying 3 μl × 2μl of DCM with a glass microcapillary (Servoprax GmbH, Germany). On the other arm, the same amount of one of the 3 pheromone solutions was also applied on disposable paper. As control for potential biases of the setup, we also presented DCM on both arms of the Y-maze. The maze stem was covered with untreated paper. We then counted and removed all ants which crossed a line 9 cm inwards of either arm for 10 min. After the test, the ants were put back into the colony. Each solution was tested separately on both sides to control for side biases.

**FIGURE 1:**
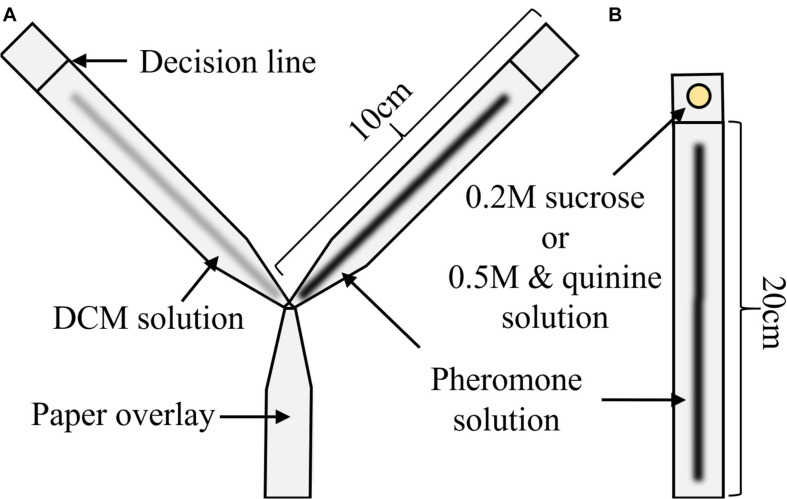
Setups used during the experiments. **(A)** To assess pheromone attraction, ants had to decide between a 10 cm × 1 cm arm treated with DCM (here left, gray) or pheromone (here right, black). When ants reached the decision line, their decision was scored and they were there removed from the maze. **(B)** In experiments 1 and 2, ants crossed a runway treated either with pheromone (shown in black) or DCM (not shown) to reach a sucrose syrup drop at the end of a 20 cm × 1 cm long runway. In experiment 3, the setup was identical, but instead of a 0.2 M sucrose syrup, 0.5 M sucrose containing either 58.6 or 78.1 μM quinine was presented. Please note that the applied pheromone solutions were invisible once applied and are colored here for illustration purposes.

#### Analysis

To test whether applied pheromone extracts significantly affect ants’ decisions in a Y-maze, we calculated a ratio of ants moving toward the arm with applied pheromone $\left(\frac{N\text{ants}\text{chose}\text{pheromone}}{N\text{total}}\right)$. In the DCM only control, one DCM side was picked for the enumerator. We then ran a linear mixed model (LMM) with solution, side of pheromone application, and their interaction as independent variables. To account for colony variability, we also added colony as random intercept. The model formula was:

*Ratio_(Ants chose pheromone)_ ∼ Solution_(DCM, 2, 4_, or _8 *gl/ml)*_^∗^ Side of pheromone_(left/right)_* + *random intercept_(Colony)_.*

### Experiment 1 – Food Acceptance After 4 Days of Food Deprivation

Overview: in this experiment, ants had to cross a runway treated with either pheromone-DCM or DCM-only solutions established as above. At the end of the runway, a 0.2 M sucrose syrup droplet was presented (see Figure 1B). 0.2 M sucrose was used as it was shown to lead to lower overall food acceptance by ants (Wendt et al., 2019), which should be strengthened by the fact that colonies were fed with 1 M sucrose before starvation. Lower food acceptances should reduce overall acceptance levels, allowing us to identify any positive effects of pheromone on food acceptance.

#### Procedure

The setup consisted of a 20 cm long runway which was covered by a paper overlay marked with one of the 4 solutions containing different amounts of pheromone (see Table 2). A solution was applied on the 20 cm long paper overlays by drawing a line of 6 μl × 2 μl of solution over the whole 20 cm (see above for details).

**TABLE 2:** Number of ants choosing a pheromone treated or DCM (control) treated arm of a Y-maze over 10 min.

Solution	Ants at pheromone	Ants at DCM	Ratio	Pheromone on left/total trials
DCM only	100	98	0.51	3/6
2 gl/ml	249	117	0.73	3/6
4 gl/ml	155	23	0.85	3/6
8 gl/ml	231	23	0.94	4/7

*Note that in the DCM only treatment, DCM without pheromone was present on both sides, one of which is shown in the ‘Ants at pheromone’ column.*

One ant at a time was allowed onto the setup using a bridge to cross the runway and drink at the 0.2 M sucrose syrup while being recorded from above with a Panasonic DMC-FZ1000 camera. While the ant was drinking at the feeder platform, the paper overlay of the runway was carefully replaced with a fresh one without interrupting the ant. This was done to prevent the presence of pheromone solution from affecting the pheromone deposition of the ant on its way back. Pheromone depositions were counted on the 20 cm long runway to the nest. Pheromone deposition in *L. niger* is a stereotyped behavior and deposition events can be counted by eye (Beckers et al., 1993). After each experimental run, the investigated ants were freeze killed in order to prevent pseudo-replication. The setup was cleaned using ethanol after each experimental run. All data used for analysis, except for pheromone depositions, was extracted from the recorded videos using the free video analysis software Solomon Coder (Péter, 2018). The extracted variables are described in Table 3.

**TABLE 3:** Overview of variables obtained from video analysis in experiments 1 and 2, with references supporting their link to perceived value.

Variable	Description	Support
**Food acceptance**	Scored 1 if ant touched sucrose drop and did not move for 3 s, otherwise scored 0	Detrain and Prieur, 2014; Oberhauser and Czaczkes, 2018; Wendt et al., 2019; Rossi et al., 2020
**Duration of first drinking**	Seconds spent until first drinking interruption	(Wendt and Czaczkes, 2020), also *a priori* due to its relationship with food acceptance.
**Total drinking time**	Seconds ant spent drinking overall	Josens et al., 1998; Detrain and Prieur, 2014; Wendt and Czaczkes, 2020
**Drinking interruptions**	Total number of drinking interruptions. Counted once the ant touched the sucrose drop again	Informal observations of more drinking interruptions for less-preferred food
**U-turns to food, U-turns to nest**	Number of U-turns followed by at least 2 cm walking in opposite direction. Turning back to the initial direction was not counted.	Informal observations of more U-turns when returning from less-preferred food
**Duration to food**	Seconds needed to traverse the 20 cm runway to the food	Ants run faster on pheromone-laden trails; an internal control for pheromone trail efficacy (Czaczkes et al., 2011).
**Total time on setup**	Seconds the ant spent on the setup (excluding bridge)	Informal observations of more time spent on setup for less-preferred food

#### Analysis

All statistical models followed the same structure:

*Dependent variable ∼ Independent variable* + *random intercept_(Colony)_.*

All durations were log transformed to account for right-skewed distributions and analyzed using a Gaussian (normal) error distribution, while count data (drinking interruptions, U-turns, and pheromone depositions) used a Poisson distribution or, if the model fit was poor, a negative binomial distribution (see Electronic Supplementary Material 1 for details).

Video analysis provided us with multiple variables of potential interest, some of which might co-vary. We thus picked one variable – duration of first drinking event – *a priori* as the main focus of our analysis. In addition, we tested all relevant other variables separately in an explorative analysis. Picking one variable *a priori* as our focus was important to avoid multiple hypothesis testing. We picked duration the first of drinking event, as this is analogous to the well-established food acceptance score (Oberhauser and Czaczkes, 2018; Wendt et al., 2019), but due to being a continuous variable, should be more sensitive.

### Experiment 2 – Food Acceptance After 2 Days of Food Deprivation

Due to the high food acceptance in experiment 1, we repeated the experiment after only 2 days of starvation. This was expected to lower the observed food acceptance (Oberhauser et al., 2018). Furthermore, only 3 solutions were used which covered the whole range used in this study: 2 gl/ml, 8 gl/ml, and DCM-only.

#### Procedure and Analysis

The procedure was the same as in experiment 1, except that the treated paper overlay was not exchanged while the ant was drinking, as pheromone depositions were not scored in experiment 2. The analysis was the same as in experiment 1.

### Experiment 3 – Food Acceptance of Sucrose-Quinine Solution

Perceived food value in experiments 1 and 2 may have been so high as to be close to maximum. This may have resulted in a ceiling effect, preventing additional increases in perceived value due to pheromone trails. To counter this, we reduced food acceptability here by adding small amounts of quinine. We used the same setup and procedure as in experiments 1 and 2. However, instead of presenting a 0.2 M sucrose syrup, we instead used 0.5 M sucrose and decreased its attractiveness by adding quinine (Merck KGaA, Darmstadt, Germany). We piloted the ants’ food acceptance on a serial dilution starting with a 10 mM quinine in 0.5 M sucrose solution and halving the quinine content in each step until we reached a food acceptance of around 50%, meaning that half of the ants interrupted drinking within the first 3 seconds. 50% acceptance was reached in step 8, which corresponded to a 78.1 μM quinine solution. Furthermore, we also added an 8.5 dilution to get closer to a food acceptance of ∼50%, which corresponded to a 58.6 μM quinine solution. We only tested ants on DCM-only and 4 gl/ml pheromone solutions.

#### Analysis

For the analysis, we included pheromone solution and quinine dilution as predictors as well as their interaction. As the response variable was binary (1/0), we used a binomial error distribution. The model was as follows:

*Food acceptance_(1 or0)_ ∼ Solution_(4 *gl/ml or DCM)*_^∗^ Quinine dilution_(58.6 μ*M or 78.1* μ*M)*_* + *random intercept_(Colony)_.*

As there was no evidence of a pheromone solution effect (see section “Results”), we chose to forego the extensive video analysis necessary to extract the data needed for the other analyses performed in experiments 1 and 2.

## Results

### Concentration-Dependent Pheromone Trail Attraction

An overview of the overall results can be found in Table 2. Pheromone was present on the left for 50% of trials (3 of 6) except for the 8 glands/ml condition (4 of 7) (see Table 2 and Electronic Supplementary Material 1). The four solutions differed significantly in their attraction to ants (χ^2^ = 75.79, *p* < 0.0001). Neither the side of pheromone presence nor the interaction between concentration and side had a significant effect (χ^2^ = 1.11, *p* = 0.29; χ^2^ = 3.76, *p* = 0.29, respectively). All pheromone concentrations were chosen significantly more often than by chance alone (8 gl/ml: 94.4%, *p* < 0.0001, 4 gl/ml: 85.1%, *p* < 0.0001, 2 gl/ml: 73.4%, *p* = 0.0001), while ants chose randomly when only DCM was present (51.2%, *p* = 0.99).

Pairwise comparisons showed that all concentrations attracted significantly more ants than DCM [DCM vs. 2 gl/ml, 95% confidence interval of pairwise contrast difference (CI_*diff*_) = 0.07–0.37, *p* = 0.0042; DCM vs. 4 gl/ml, (CI_*diff*_) = 0.18–0.49, *p* = 0.0001; DCM vs. 8 gl/ml, CI_*diff*_ = 0.28–0.58, *p* < 0.0001, see Electronic Supplementary Material 1], while only the strongest and weakest pheromone concentrations differed significantly in their attraction (8 gl/ml vs. 2 gl/ml, CI_*diff*_ = 0.06–0.36, *p* = 0.005).

### Experiment 1 – Food Acceptance After 4 Days of Food Deprivation

In total, 91 ants from 6 colonies were tested. *Duration to food* was significantly different between solutions (χ^2^ = 32.34, *p* < 0.0001), with all pheromone solutions leading to significantly shorter time spent on the runway than the control DCM-only solution [DCM vs. 2 gl/ml, 95% confidence interval of pairwise contrast ratio (CI_*ratio*_) = 0.55–0.83, *p* < 0.0001; DCM vs. 4 gl/ml, CI_*ratio*_ = 0.58–0.88, *p* = 0.0006; DCM vs. 8 gl/ml, CI_*ratio*_ = 0.54–0.81]. The number of *U-turns on the way to the food* did not differ between treatments (χ^2^ = 4.18, *p* = 0.24). These measures are taken before the food is encountered, and so do not reflect perceived value of the food source.

Ants had a very high food acceptance, with only one ant interrupting drinking within the first 3 s. However, pheromone deposition was very low, with only 10 ants depositing pheromone on the way back. Thus, those two variables were not used for analysis.

**FIGURE 2:**
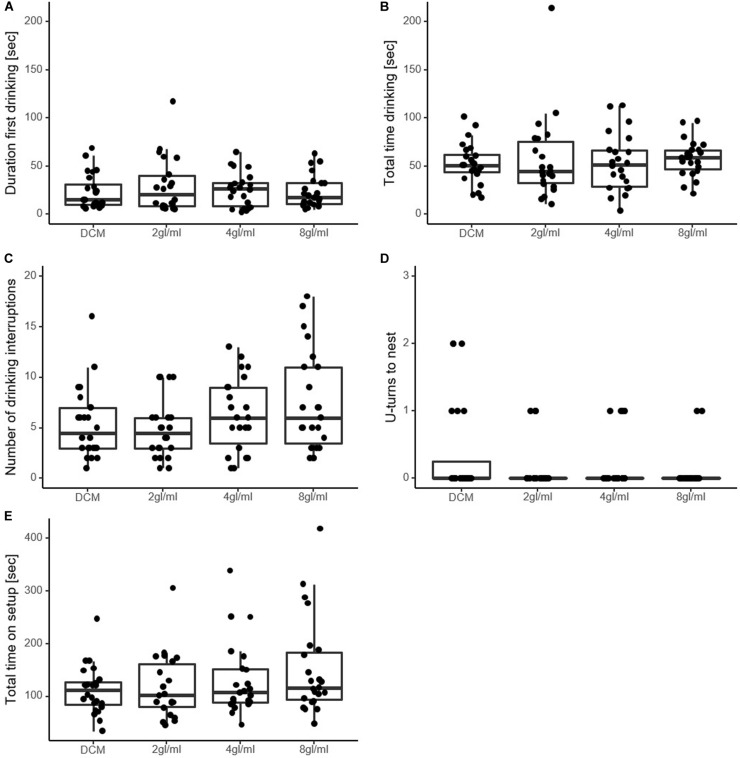
Measures of perceived food value in experiment 1. **(A)** Seconds ants spent drinking until they first moved away from the sucrose drop. **(B)** Seconds spent drinking in total. **(C)** Number of drinking interruptions over the course of the trial. **(D)** Number of U-turns made by the ant on the way back to the nest. Was only counted when ants moved at least 2 cm in an opposite direction. The majority of ants did not perform U-turns. **(E)** Seconds the ants spent on the setup in total. Note that none of the variables differed significantly between the solutions, except in **(C)**, where 8 gl/ml is significantly higher than DCM. DCM, dichloromethane solvent; Xgl/ml, x pheromone glands per milliliter DCM.

The *duration of the first drinking event* did not reveal any significant effect of pheromone (χ^2^ = 0.39, *p* = 0.94, see Figure 2A). Similarly, *total drinking time* and *total time on setup* also did not differ significantly among solutions (χ^2^ = 3.08, *p* = 0.38; χ^2^ = 5.23, *p* = 0.15, respectively, Figures 2B,E).

Furthermore, the *number of interruptions* differed significantly between the solutions (χ^2^ = 14.73, *p* = 0.0021, Figure 2C). Pairwise analysis revealed significantly more interruptions in the highest pheromone concentration (8 gl/ml) than the DCM-only control (CI_*ratio*_ = 1.04–1.82, *p* = 0.0186), while the other two pheromone solutions did not differ significantly from DCM (2 gl/ml vs. DCM, CI_*ratio*_ = 0.65–1.22, *p* = 0.73; 4 gl/ml vs. DCM, CI_*ratio*_ = 0.86–1.55, *p* = 0.48). The number of U-turns on the way back to the nest did not differ significantly from DCM (χ^2^ = 4.51, *p* = 0.21, Figure 2D).

### Experiment 2 – Food Acceptance After 2 Days of Food Deprivation

In total, 46 ants from 6 colonies were tested. Surprisingly, the *duration to food* showed no difference between solutions (χ^2^ = 2.56, *p* = 0.28). The number of U-turns to the food differed significantly (χ^2^ = 6.69, *p* = 0.0352). This difference was caused by significantly more U-turns in the presence of the highest pheromone concentration compared to DCM (8 gl/ml vs. DCM, CI_*ratio*_ = 1.04–5.15, *p* = 0.0381; 2 gl/ml vs. DCM, CI_*ratio*_ = 0.58–3.31, *p* = 0.57).

Ants again had a very high food acceptance, with only three ants not accepting the food. As in experiment 1, the *duration of the first drinking event* did not reveal any significant effect of pheromone (χ^2^ = 1.22, *p* = 0.54). This was also the case for the *total drinking time* (χ^2^ = 4.22, *p* = 0.12) and *total time spent at setup* (χ^2^ = 0.52, *p* = 0.77). Unlike in experiment 1, the number of interruptions did not differ significantly between the solutions (χ^2^ = 1.79, *p* = 0.41). The number of *U-turns* to the nest also showed no difference between solutions (χ^2^ = 3.1, *p* = 0.21).

### Experiment 3 – Food Acceptance of Sucrose-Quinine Solution

**FIGURE 3:**
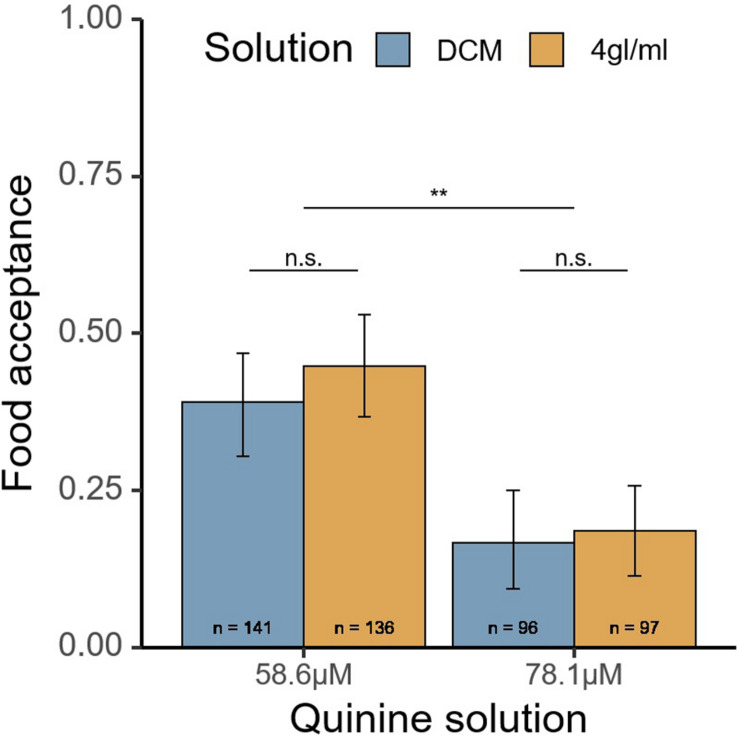
Food acceptance of the quinine solution in experiment 3 after passing a runway treated with pheromone (4 gl/ml) or DCM solvent (DCM). Food acceptance of 1 would correspond to uninterrupted drinking in the first 3 s. Quinine solution corresponds to XμM of quinine in 0.5 M sucrose syrup. 4 gl/ml, 4 pheromone glands per milliliter DCM. **p < 0.01; n.s., not significant.

In total, 470 ants from 11 colonies were tested. No difference in food acceptance was found between the 4 gl/ml and DCM-only solutions (χ^2^ = 0.3, *p* = 0.59, see Figure 3). The lower quinine concentration (58.6 μM), unsurprisingly, had significantly higher acceptance (χ^2^ = 9.81, *p* = 0.0017).

## Discussion

Over a series of experiments we tested whether the presence of social information, in the form of pheromone trails advertising high-value food, influences perceived value in an ant. However, although there was a good reason to expect such an effect (see section “Introduction”), we found no evidence for such a value-distortion effect and conclude that pheromone trail presence does not distort value perception in *L. niger.* We base this conclusion primarily on two lines of evidence which we defined *a priori* as our main variables of interest: Lack of increase in first drinking event length (experiments 1 and 2, Figure 2A), and lack of change in acceptance rates (experiment 3, Figure 3), both variables which correlate with perceived value (see Table 3). However, other factors which are expected to correlate with perceived value, but were analyzed only in an explorative manner, also showed no effect. These include duration of time spent feeding (Figure 2B) and total time spent on the setup (Figure 2E). However, the pheromone trails we created were clearly biologically active: ants followed them in a dose-dependent manner and showed some evidence of running faster on pheromone trails in experiment 1, but not in experiment 2. While the artificial trails used lacks some aspects of a naturally laid trail (the dotted two-dimensional structure, concurrent cuticular hydrocarbons), we believe that it mimics real trails well enough for us to make strong conclusions.

Nonetheless, one comparison returned significant effects: in experiment 1 (but not experiment 2), we found significantly more feeding interruptions in the 8 gland/ml treatment as compared to DCM-only controls. Taken at face value, this result would suggest that extremely high levels of trail pheromone may be aversive. However, the lack of concurrence between the almost identical experiments 1 and 2, the small difference, and the broad confidence intervals of the estimates, leads us to place little weight on this finding – especially given that these effects are only present at the highest pheromone trail strength. More critically, the vast majority of the evidence implies no effect. This includes the very highly powered experiment 3 (*n* = 470), which showed very good sensitivity to small changes in aversive substance, but no evidence of value distortion due to pheromone presence. In addition, a separate study (Oberhauser et al., in preparation) also showed no value distortion effects of pheromone, using identical associative learning methods to those that have been used successfully to detect many such effects in the same species (Czaczkes et al., 2018; De Agrò et al., 2019, 2020; Wendt et al., 2019, 2020; Wendt and Czaczkes, 2020). Thus, while completely proving a lack of effect is not possible, we are nonetheless quite confident that pheromone trail presence does not influence perceived value in *L. niger*.

In drawing this conclusion, we are making the assumption that any value-distorting effect would be linear, in that we only tested trails equivalent to a well-established trail (4 glands per ml), half that strength, and double that strength (von Thienen et al., 2014). We chose strong trails in order to detect even very weak effects, if they exist. However, it is theoretically possible that such value distortion effects would exist for very weak pheromone trails, but disappear again at strong but natural levels. We can conceive of no convincing biological explanation for why this should be the case, and so remain confident about the lack of effect we describe.

We originally hypothesized that conformism would be seen in ants, whereby social information suggesting a high-value resource (a strong pheromone trail) would increase the perceived value of this resource, since such effects are strong in humans (Cohen and Golden, 1972; Bone, 1995; Shi et al., 2016; Jayles et al., 2017), as long as the difference between the social ratings and the absolute value of the product is small. Recent research has found many parallels between value perception in humans and insects (Shafir et al., 2002; Sasaki and Pratt, 2011; Tan et al., 2015; Czaczkes et al., 2018; Oberhauser and Czaczkes, 2018; Wendt et al., 2019), even resulting in irrational behavior, suggesting that a further parallel was likely. Reliance on social information to make valuation decisions is common in animals (Kendal et al., 2004; Danchin et al., 2018; Otake and Dobata, 2018), especially when no other useful information sources are available. Studies on honeybees report that alarm and Nasanov (attractive) pheromones can modulate the response to positive and aversive stimuli (Baracchi et al., 2017, 2020; Rossi et al., 2018).

Importantly, a study conducted in parallel to our own (Rossi et al., 2020) found just such an effect when studying the Argentine ant *Linepithema humile*. This study found that Argentine ants pre-exposed to artificial pheromone trails showed higher food acceptance for a range of sucrose solution concentrations, although they found no effect on feeding duration. While the methodologies differed (pre-exposure was away from the feeding context, and artificial pheromone trails were used which may be much stronger than naturally formed trails), we believe the reason for the different findings is the species used. The contrast between our results and those reported by Rossi et al. (2020) mirrors a similar contrast between the two species in pheromone use: when making navigational decision, *L. niger* relies much more strongly on private information (route memory) than pheromone trail information, when the two conflict (Aron et al., 1993; Grüter et al., 2011; von Thienen et al., 2016). By contrast, *Li. humile* relies more strongly on pheromone information, and follows that preferentially when it conflicts with route memory (von Thienen et al., 2016). It is interesting that this differential reliance on social information extends beyond navigation into subjective resource evaluation. However, honeybees tend, like *L. niger*, to ignore social information if it conflicts with private information (Grüter et al., 2008), and yet show robust modulation of subjective reward or punishment evaluation due to social information (Baracchi et al., 2017, 2020; Rossi et al., 2018).

Most demonstrations of parallels between human and animal behavior have generally been in the context of individual decision-making. *L. niger* make coordinated collective decisions, based on the evaluations of individuals. Conformism in resource evaluation during collective decision-making would likely be detrimental, as it reduces the number of independent evaluations available, and thus the accuracy of collective decisions (Pratt and Sasaki, 2018). Indeed, it is precisely independent evaluation of resources which allows collective decision-making by insects to side-step individual-level cognitive biases, and enables rational collective decision-making to emerge from decisions by individuals that fall prey to cognitive fallacies (Sasaki and Pratt, 2011; Sasaki et al., 2018, 2020). One exception may be in the formation of quorums during collective decision-making, where evaluation conformism would result in positive feedback, and potentially speed up quorum formation for suitable options (Bose et al., 2017; Marshall et al., 2019). From the results of Rossi et al. (2020), we predict that more conformist Argentine ants will be more likely to make irrational collective decisions than *L. niger*, which in turn will be more likely to make irrational collective decisions than the *Temnothorax* ants studied by Sasaki and Pratt (2011) and Sasaki et al. (2020).

More broadly, during resource evaluation where the attributes of the resource are clear, there is little benefit to using social information. Social information becomes valuable when gaining high-quality personal information is costly, or when it is not available (Danchin et al., 2004; Kendal et al., 2005, 2009). As the ant has already paid any costs associated with gaining personal information, there would seem to be no additional reason to attend to social information which give less reliable readings of the same resource attributes. It is possible that Argentine ants respond positively to such social information in order to build in an addition positive feedback loop, accelerating collective decision-making as the cost of increased inflexibility. Alternatively, modulating subjective evaluation due to (potentially irrelevant) social information may be a pleiotropic effect which is not repressed in some species.

Evaluation seems to be unaffected by social information signaling resource value. However, pheromone deposition is: *L. niger* ants deposit less pheromone when encountering nestmates on trails (Czaczkes et al., 2013b), at food sources (Wendt et al., 2020), and on pheromone-marked paths (Czaczkes et al., 2013a, 2016). This implies that ants can disentangle their evaluation of a resource from their recruitment to it: ants can lower pheromone deposition when encountering many other foragers, or on paths with pheromone trails, without changing their evaluation of the food they are returning to. This may allow them to make more accurate foraging decisions in the future. For example, ants modify their perceived value of a food source after being offered substantially better (or worse) food from nestmates (Wendt et al., 2019), and may use this information to decide whether to continue exploiting a known food location or try a new one (Czaczkes et al., 2019).

While pheromone trails (social signals) do not influence the perceived value of a food source for *L. niger*, nestmate presence at the food source, a social cue, does seem to have such an effect: Ants prefer to forage on otherwise identical food sources which are not accompanied by nestmate presence (Wendt et al., 2020). These results seem inconsistent. We interpret these differences as arising from the very different temporal nature of the information. Pheromone trails are long lasting – a strong *L. niger* trail is detectable by ants after at least 8 h (Evison et al., 2008). They thus can become outdated, providing false exploitation-level information if exploitation level changes – for example due to a brief spell of inclement weather. Ant colonies can become ‘trapped’ by outdated pheromone trail information (Beckers et al., 1990; Latty and Beekman, 2013). By contrast, nestmate encounters provide an instantaneous reading of exploitation level. Ants may thus do well to attend to the instantaneous information when making foraging choices, but may ignore the possibly outdated pheromone information. This is especially likely as, in the current experiment, the ants were tested alone, so had first-hand information that the resource was underexploited, and that the pheromone information did not match current foraging conditions.

In conclusion, we found no effect of pheromone trail presence on the perceived value of a food source. This is in contrast to results reported in other ant species (Rossi et al., 2020), honeybees (Baracchi et al., 2017, 2020; Rossi et al., 2018), and even a moth (Hostachy et al., 2019). Why some animals in some situations are influenced by social information, when other animals in the same situation or the same animal in a different situation are not, is a major question which remains to be tackled.

## Data Availability Statement

All datasets generated for this study are included in the article/Supplementary Material.

## Author Contributions

FO, SW, and TC conceived the experiments. FO conducted the experiments and analyzed and visualized the data. All authors wrote the first draft, revised and approved the final manuscript.

## Conflict of Interest

The authors declare that the research was conducted in the absence of any commercial or financial relationships that could be construed as a potential conflict of interest. The reviewer CG declared a past co-authorship with one of the authors TC to the handling editor.
