# Supplementary material for: Trail Pheromone Does Not Modulate Subjective Reward Evaluation in Lasius niger Ants
Source: Front Psychol. 2020 Sep 23;11:555576. doi: 10.3389/fpsyg.2020.555576 (PMC7540218; doi:10.3389/fpsyg.2020.555576)
Supplement: Supplementary file 1 [file Data_Sheet_1.PDF]

ESM 1 - data handling protocol

Supplement to: Social information in the form of pheromone trails does not distort perceived value in ants

Oberhauser FB, Wendt S & Czaczkes TJ

Corresponding author: Felix.Oberhauser@outlook.com

Contents

Please note 2

Preparation 3
- R version 3
- Libraries 3
- Set colour and aesthetics for plots 3
- Create table function 3
- Modify DHARMa::testResiduals() function 4
  - Add residual vs. predictors function 4

Prerequisite - Pheromone concentration dependent attraction 4
- Load data 4
- Sample size 4
- Overview 5
- Analysis 5

Experiment 1 - Food acceptance after four days of starvation 11
- Load data 11
- Sample size 11
- Analysis 11
  - Food acceptance 11
  - Pheromone depositions 12
  - Duration of first drinking 12
    - Results 17
  - Total drinking time 18
    - Results 21
  - Interruptions 22
    - Results 25
  - U-turns to food 26
    - Results 29
  - U-turns to nest 30
    - Results 33
  - Duration to food 34
    - Results 37
  - Total time on setup 38
    - Results 41
  - Panel figure 42

Experiment 2 - Food acceptance after two days of starvation **43**
Load data . . . 43
Sample size . . . 43
Analysis . . . 44
Food acceptance . . . 44
Duration of first drinking . . . 44
Results . . . 47
Total drinking time . . . 48
Results . . . 51
Interruptions . . . 52
Results . . . 57
U-turns to food . . . 58
Results . . . 61
U-turns to nest . . . 62
Results . . . 65
Duration to food . . . 66
Results . . . 69
Total time on setup . . . 70
Results . . . 73
Panel figure . . . 74

Experiment 3 - Food acceptance of sucrose-quinine solution **75**
Load data . . . 75
Sample size . . . 75
Dilution . . . 76
Analysis . . . 76
Food acceptance . . . 76
Results . . . 82
Figure . . . 83

Package information **84**
Package versions . . . 84
Package references . . . 84

Please note

This protocol leads through all analysis steps and results presented in the manuscript. It contains commentary, but has not been optimised for legibility.

The pipe command "%>%" is used to help the understanding of coding steps. The pipe forwards the output of one function to the next. Example:

This code

```
mean(c(1,2,3))
```

```
## [1] 2
```

is the same as this

```
c(1,2,3) %>%
  mean()
```

```
## [1] 2
```

Preparation

R version

```r
R.version.string
```

```
## [1] "R version 3.6.3 (2020-02-29)"
```

Libraries

```r
lib <- c(
  "xlsx",      # loading xlsx files
  "ggplot2",   # plot data
  "cowplot",   # create panel plots
  "knitr",     # display tables
  "DHARMa",    # model validation
  "glmmTMB",   # modelling GLMMs
  "car",       # ANOVA
  "emmeans",   # contrasts
  "dplyr",     # data restructuring
  "janitor")   # adding summary row to tables

lapply(lib, require, character.only = TRUE)
```

Set colour and aesthetics for plots

```r
col <- c("#7a9eba","#DEA757")
theme_set(
  theme_classic(20) +
  theme(
    legend.position = c(.5, .95),
    legend.direction = "horizontal",
    legend.background = element_blank()
    )
)
```

Create table function

```r
create_table <- function(data,
                         column_names = NA,
                         caption = NULL,
                         alignment = "c",
                         digits = 2) {
  return(
    kable(
      data,
      digits = digits,
      align = alignment,
      caption = caption,
      col.names = column_names)
  )
}
```

Modify DHARMa::testResiduals() function

As standard, the function prints AND returns test results. We deactivate one to reduce redundancy. Furthermore, we combine two functions, so we do not need to call the plot(simulationoutput) function separately.

```
checkmodel<-function(simulationOutput){
  plot(simulationOutput)

  testResiduals <-
    function (simulationOutput){
    oldpar = par(mfrow = c(1, 2)) #show only two plots each row
    out = list()
    out$uniformity = testUniformity(simulationOutput, plot = F) #deactivate plot
    out$dispersion = testDispersion(simulationOutput)
    out$outliers = testOutliers(simulationOutput)
    par(oldpar)
    #print(out) deactivated
    return(out)}

  testResiduals(simulationOutput)
}
```

Add residual vs. predictors function

To plot all predictors against the model residuals, we add another function.

```
plot_predictors <-function(data, simulatedResiduals, predictors){
  for (predict in predictors){
    plot(data[[predict]], simulatedResiduals$scaledResiduals,
         xlab = predict,
         ylab = "scaled residuals")
  }
}
```

Prerequisite - Pheromone concentration dependent attraction

Load data

```
concentration <- read.xlsx("ESM2_raw_data.xlsx", sheetIndex = 1)
concentration$Solution <- factor(concentration$Solution,
                                 levels = c("DCM only",
                                            "2gl/ml",
                                            "4gl/ml",
                                            "8gl/ml"))
```

Sample size

```
concentration %>%
  group_by(Solution) %>%
  mutate(
    side_num = ifelse(Side.of.pheromone == "l",
                      1,
                      0)) %>%
  summarise(
    tested_ants = sum(Ants.at.DCM.side, Ants.at.pheromone.side),
```

```
    N = length(levels(droplevels(Colony))),
    trials = paste(sum(side_num), "/", length(side_num))) %>%

  create_table(
    column_names =
      c("Solution",
        "Number of tested ants",
        "Number of colonies",
        "Pheromone on left / total trials"))
```

Solution	Number of tested ants	Number of colonies	Pheromone on left / total trials
DCM only	198	3	3 / 6
2gl/ml	366	2	3 / 6
4gl/ml	178	3	3 / 6
8gl/ml	254	4	4 / 7

Overview

Do solutions differ in their attractiveness to ants? Here, we see the ratio of ants going to the side of the maze with the solution applied.

```
concentration %>%
  mutate(ratio_pheromone =
           round(
             Ants.at.pheromone.side /
               (Ants.at.pheromone.side + Ants.at.DCM.side),
             4)) %>%
  {. ->> concentration} #save updated data.frame

concentration %>%
  select(-Date,-Colony, -Side.of.pheromone) %>%
  group_by(Solution) %>%
  summarise(
    `Ants at Pheromone` = sum(Ants.at.pheromone.side),
    `Ants at DCM` = sum(Ants.at.DCM.side),
    Ratio = mean(ratio_pheromone)) %>%
  create_table(digits = 2)
```

Solution	Ants at Pheromone	Ants at DCM	Ratio
DCM only	100	98	0.51
2gl/ml	249	117	0.73
4gl/ml	155	23	0.85
8gl/ml	231	23	0.94

Analysis

```
m<-glmmTMB(
    ratio_pheromone ~ Solution * Side.of.pheromone + (1|Colony),
    family="gaussian",
    data=concentration)
```

Check model

```
mres<-simulateResiduals(m)
checkmodel(mres)
```

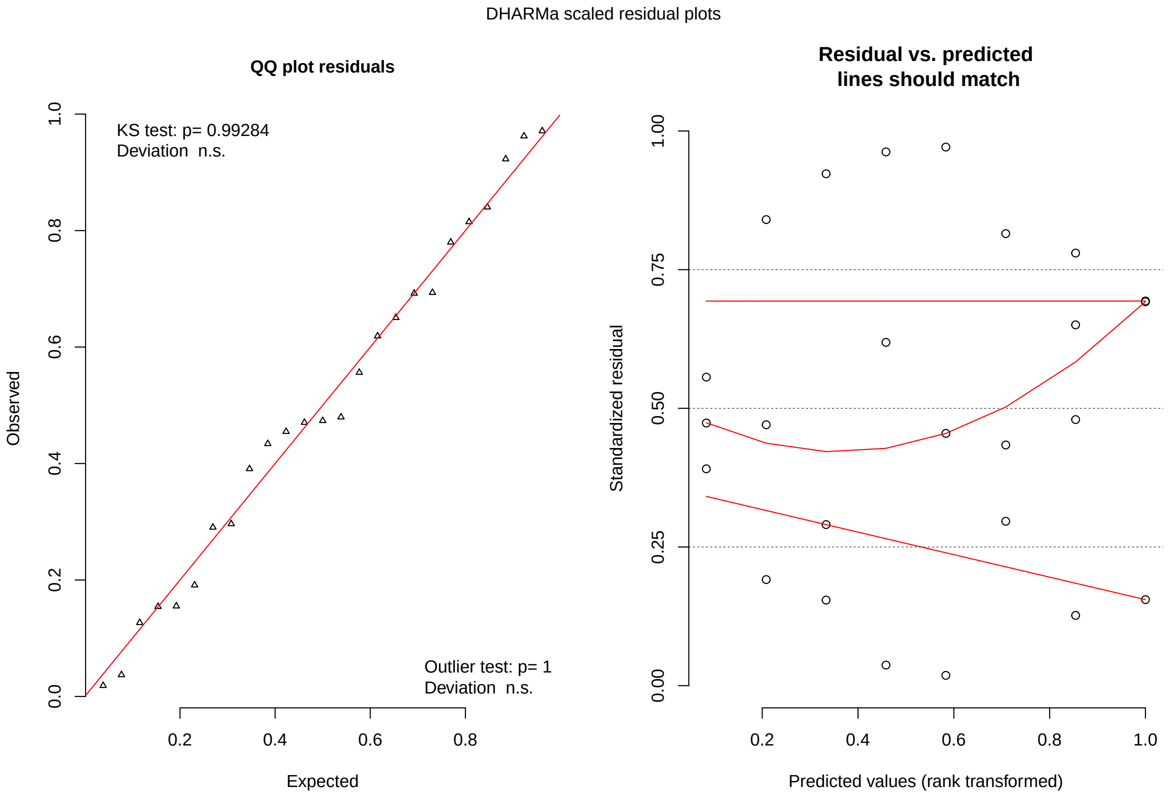

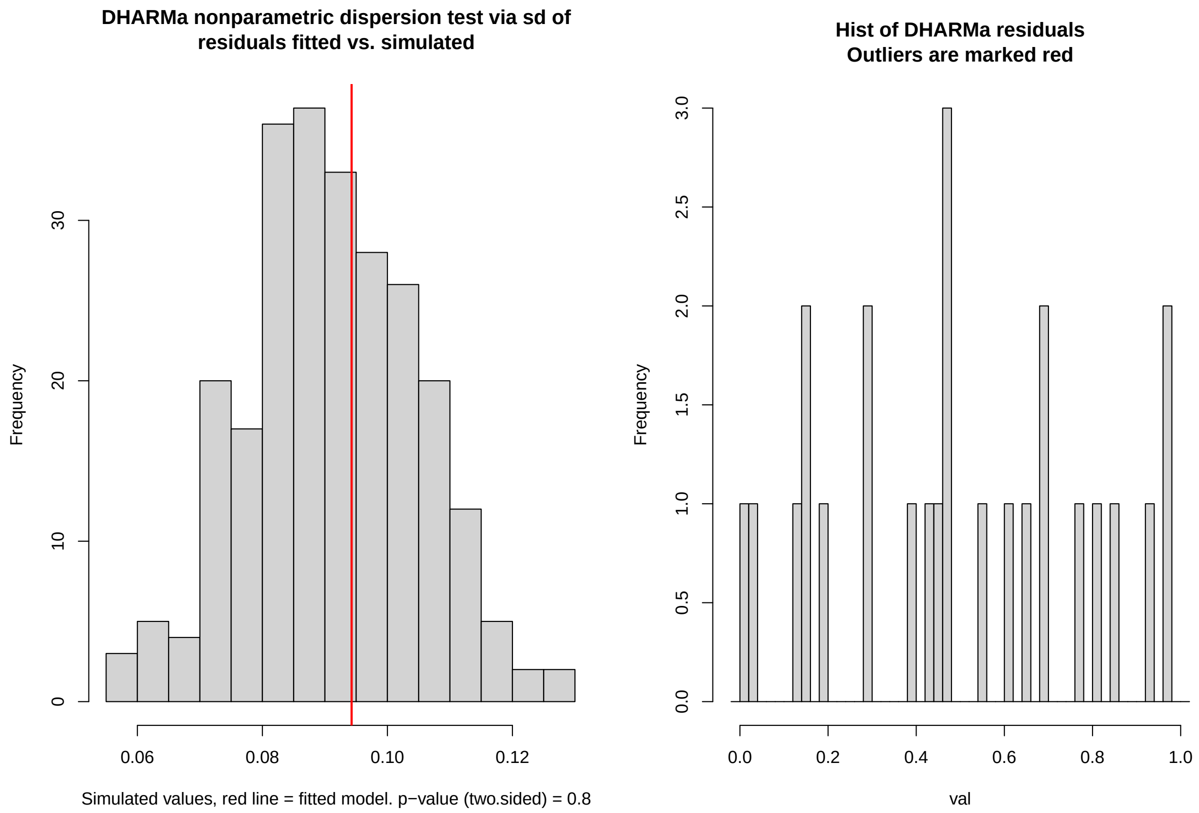


```
## $uniformity
##
##  One-sample Kolmogorov-Smirnov test
##
## data:  simulationOutput$scaledResiduals
## D = 0.080285, p-value = 0.9928
## alternative hypothesis: two-sided
##
##
## $dispersion
##
##  DHARMa nonparametric dispersion test via sd of residuals fitted vs.
##  simulated
##
## data:  simulationOutput
## ratioObsSim = 1.0351, p-value = 0.8
## alternative hypothesis: two.sided
##
##
## $outliers
##
##  DHARMa outlier test based on exact binomial test
##
## data:  simulationOutput
## outLow = 0.0000000, outHigh = 0.0000000, nobs = 25.0000000, freqH0 =
## 0.0039841, p-value = 1
## alternative hypothesis: two.sided
```

```r
plot_predictors(data = concentration,
                simulatedResiduals = mres,
                predictors = c("Solution", "Side.of.pheromone"))
```

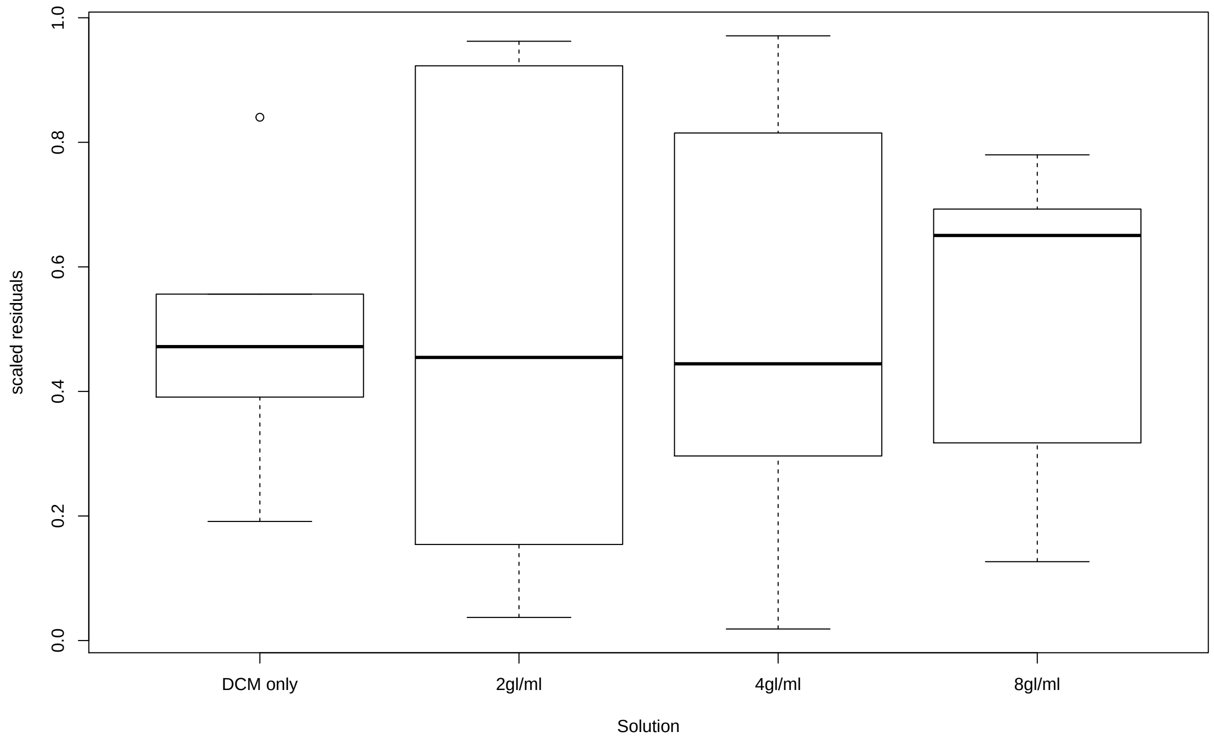

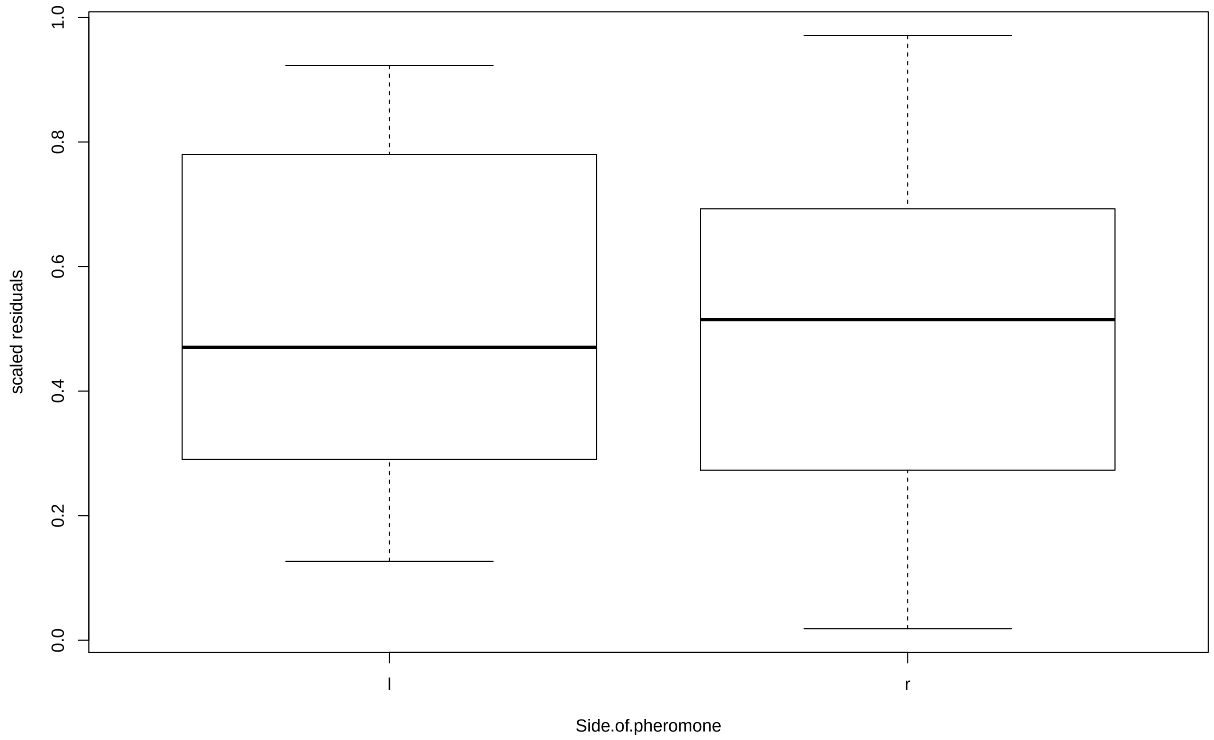

We can see more variation in the pheromone treatments.

```
Anova(m)
```

```
## Analysis of Deviance Table (Type II Wald chisquare tests)
##
## Response: ratio_pheromone
##                              Chisq Df Pr(>Chisq)
## Solution                   75.7904  3  2.453e-16 ***
## Side.of.pheromone           1.1102  1     0.2920
## Solution:Side.of.pheromone  3.7620  3     0.2883
## ---
## Signif. codes:  0 '***' 0.001 '**' 0.01 '*' 0.05 '.' 0.1 ' ' 1
```

No effect of the side the pheromone was applied. The solutions differ significantly, however.

We now take a look at the model results.

```
emmip(m, ~ Solution, CIs = T, plotit = F) %>%
  ggplot(aes(x=Solution, y=yvar))+
  geom_point()+
  geom_errorbar(aes(ymax=UCL, ymin=LCL, width=.1))+
  ylab("Predicted proportion of ants choosing pheromone")+
  coord_cartesian(ylim = c(0,1))+
  geom_hline(yintercept = .5, linetype = "dashed")
```

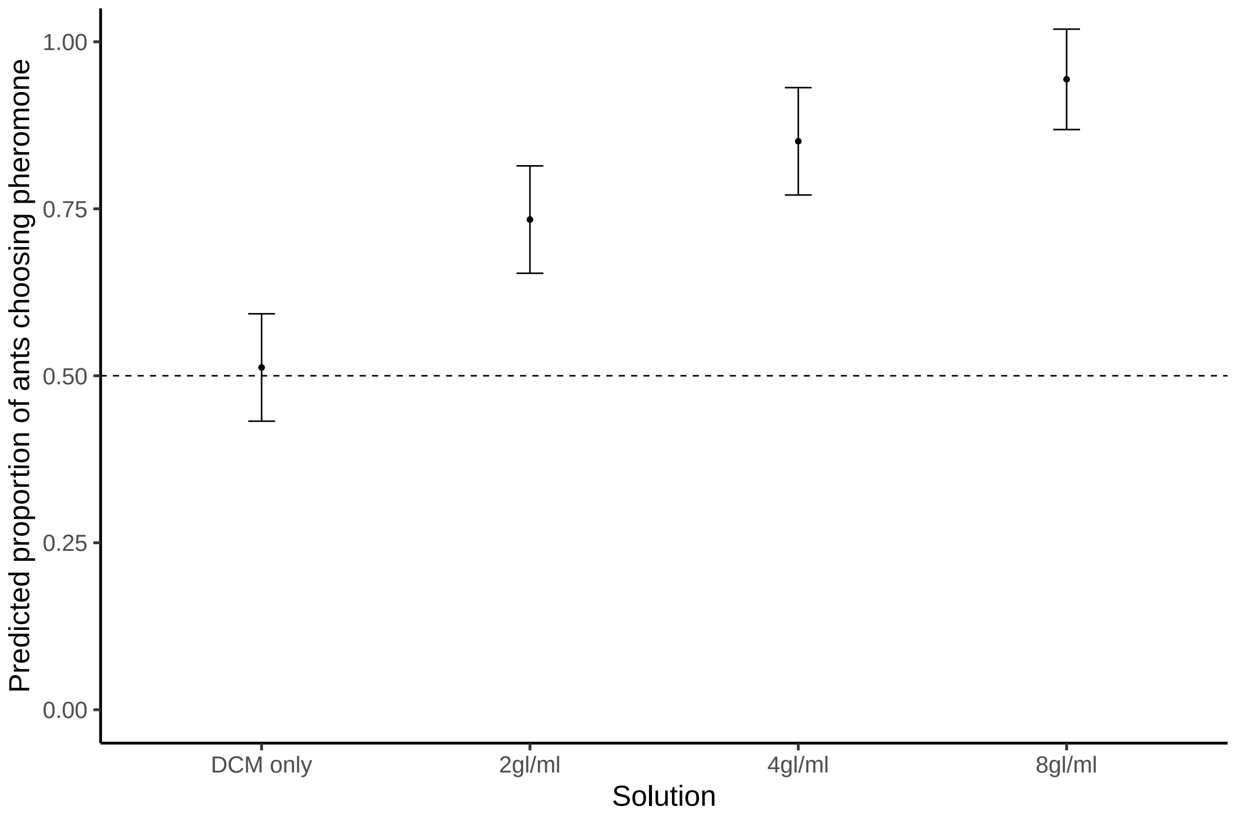

We see an increase of ants choosing the pheromone the higher the concentration. Which of the concentrations were significantly different from chance (50%)?

```
emm <- emmeans(m, ~Solution)
#test against chance level (50%)
test(emm, null=0.5, adjust = "tukey")
```

```
##  Solution emmean     SE df null t.ratio p.value
##  DCM only  0.512 0.0377 15  0.5   0.329  0.9959
##  2gl/ml    0.734 0.0377 15  0.5   6.202  0.0001
##  4gl/ml    0.851 0.0377 15  0.5   9.309  <.0001
##  8gl/ml    0.944 0.0353 15  0.5  12.581  <.0001
##
## Results are averaged over the levels of: Side.of.pheromone
## P value adjustment: sidak method for 4 tests
```

All of the solutions were chosen significantly more often than chance. Did they also differ from each other?

```
#pairwise comparisons
contrast(emm, method = "pairwise", adjust = "mvt")
```

```
##  contrast          estimate     SE df t.ratio p.value
##  DCM only - 2gl/ml  -0.2215 0.0533 15 -4.153  0.0041
##  DCM only - 4gl/ml  -0.3386 0.0533 15 -6.350  0.0001
##  DCM only - 8gl/ml  -0.4314 0.0516 15 -8.354  <.0001
##  2gl/ml - 4gl/ml    -0.1172 0.0533 15 -2.197  0.1688
##  2gl/ml - 8gl/ml    -0.2099 0.0516 15 -4.066  0.0049
##  4gl/ml - 8gl/ml    -0.0928 0.0516 15 -1.796  0.3129
##
## Results are averaged over the levels of: Side.of.pheromone
```

```
## P value adjustment: mvt method for 6 tests
```

```
confint(contrast(emm, method = "pairwise"))
```

```
##  contrast           estimate     SE df lower.CL upper.CL
##  DCM only - 2gl/ml   -0.2215 0.0533 15   -0.375  -0.0678
##  DCM only - 4gl/ml   -0.3386 0.0533 15   -0.492  -0.1849
##  DCM only - 8gl/ml   -0.4314 0.0516 15   -0.580  -0.2826
##  2gl/ml - 4gl/ml     -0.1172 0.0533 15   -0.271   0.0365
##  2gl/ml - 8gl/ml     -0.2099 0.0516 15   -0.359  -0.0611
##  4gl/ml - 8gl/ml     -0.0928 0.0516 15   -0.242   0.0561
##
## Results are averaged over the levels of: Side.of.pheromone
## Confidence level used: 0.95
## Conf-level adjustment: tukey method for comparing a family of 4 estimates
```

Only the lowest and highest concentration differ significantly.

Experiment 1 - Food acceptance after four days of starvation

Load data

```
exp1 <- read.xlsx("ESM2_raw_data.xlsx", sheetIndex = 2)
exp1$Solution<-factor(
  exp1$Solution,
  levels = c("DCM only", "2gl/ml", "4gl/ml", "8gl/ml"))
```

Sample size

```
exp1 %>%
  group_by(Solution) %>%
  tally() %>%
  adorn_totals("row") %>%
  create_table()
```

Solution	n
DCM only	24
2gl/ml	22
4gl/ml	22
8gl/ml	23
Total	91

Number of colonies tested:

```
length(levels(exp1$Colony))
```

```
## [1] 6
```

Analysis

Food acceptance

Food acceptance is defined as: 1 = drinking for at least 3 seconds after first touching food 0 = move away from food within first 3 seconds

```
exp1 %>%
  mutate(Food.acceptance = ifelse(Duration.of.first.drinking >= 3, 1, 0)) %>%
  {.->>exp1} %>%
  group_by(Solution) %>%
  summarise(percent_accepting_food = mean(Food.acceptance)*100,
            n = length(Food.acceptance),
            not_accepting = n - sum(Food.acceptance)) %>%
  create_table(column_names = c("Solution", "% accepting food",
                                "Total ants", "Not accepting"),
               digits = 1)
```

Solution	% accepting food	Total ants	Not accepting
DCM only	100.0	24	0
2gl/ml	100.0	22	0
4gl/ml	95.5	22	1
8gl/ml	100.0	23	0

We see that only one ant did not accept the food immediately. This parameter is unsuitable for testing.

Pheromone depositions

Ants often do not deposit on the first way back from food. How many deposited?

```
exp1 %>%
  mutate(deposited_pheromone = ifelse(Pheromone.depositions > 0,
                                      1,
                                      0)) %>%
  {.->>exp1} %>%
  group_by(Solution) %>%
  summarise(n = length(deposited_pheromone),
            percent_depositing_pheromone = mean(deposited_pheromone)*100,
            `depositing ants` = sum(deposited_pheromone)) %>%
  create_table(column_names = c("Solution", "Total ants",
                                "Percent depositing pheromone",
                                "Number of depositing ants"))
```

Solution	Total ants	Percent depositing pheromone	Number of depositing ants
DCM only	24	16.67	4
2gl/ml	22	4.55	1
4gl/ml	22	18.18	4
8gl/ml	23	4.35	1

Not suitable for analysis.

Duration of first drinking

Time in seconds until ant disrupted drinking

```
mfirst<- glmmTMB(Duration.of.first.drinking ~ Solution + (1|Colony),
                 family = "gaussian",
                 data = exp1)
mfirstres <- simulateResiduals(mfirst)
checkmodel(mfirstres)
```

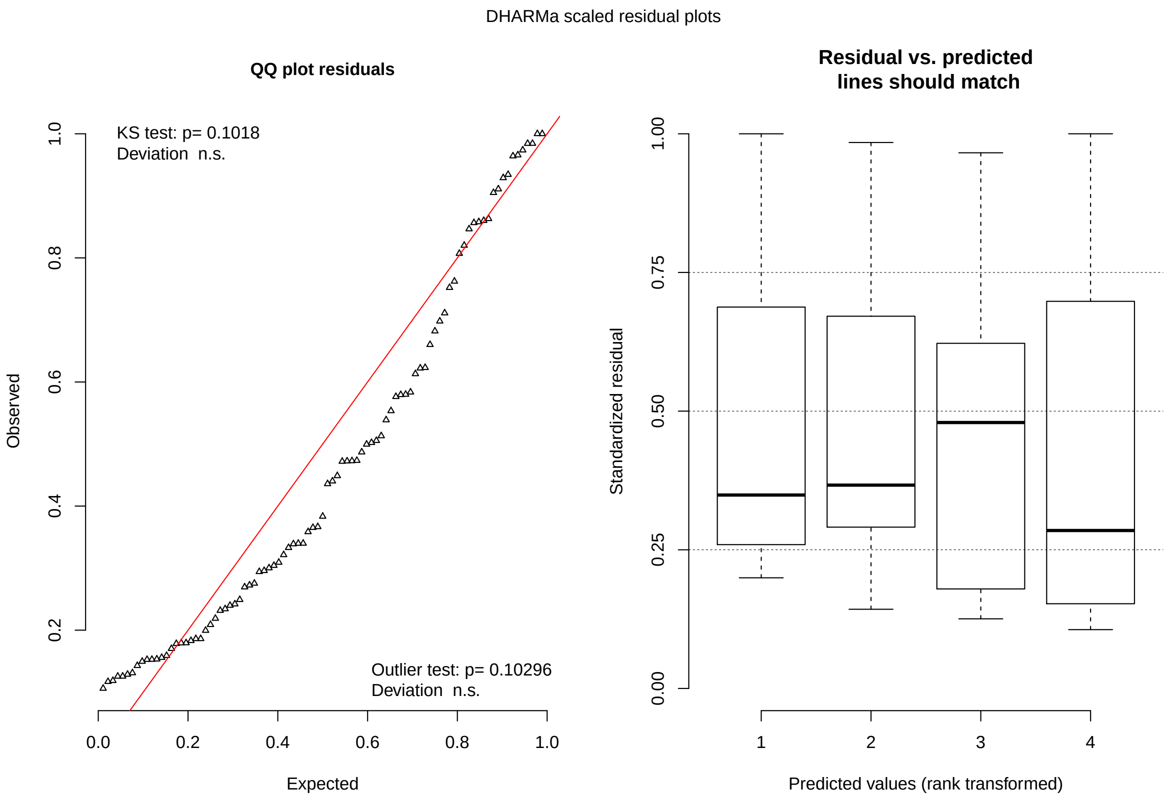
DHARMa scaled residual plots
QQ plot residuals
KS test: p= 0.1018
Deviation n.s.
Outlier test: p= 0.10296
Deviation n.s.
Observed
Expected
0.0
0.2
0.4
0.6
0.8
1.0
Residual vs. predicted
lines should match
Standardized residual
0.00
0.25
0.50
0.75
1.00
1
2
3
4
Predicted values (rank transformed)

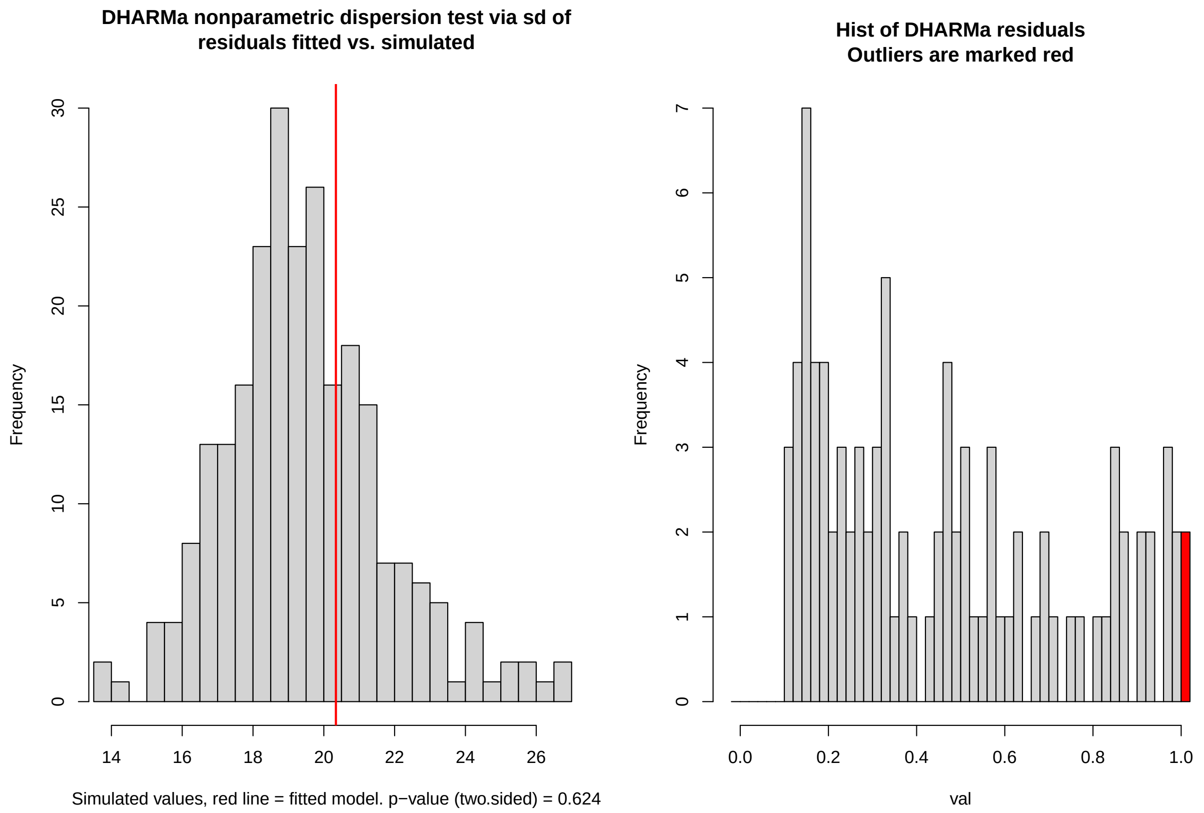


```
## $uniformity
##
##  One-sample Kolmogorov-Smirnov test
##
## data:  simulationOutput$scaledResiduals
## D = 0.12791, p-value = 0.1018
## alternative hypothesis: two-sided
##
##
## $dispersion
##
##  DHARMa nonparametric dispersion test via sd of residuals fitted vs.
##  simulated
##
## data:  simulationOutput
## ratioObsSim = 1.0444, p-value = 0.624
## alternative hypothesis: two.sided
##
##
## $outliers
##
##  DHARMa outlier test based on exact binomial test
##
## data:  simulationOutput
## outLow = 0.0000000, outHigh = 2.0000000, nobs = 91.0000000, freqH0 =
## 0.0039841, p-value = 0.103
## alternative hypothesis: two.sided
```

Not ideal residual spread (a lot of low values), we now take the log of drinking time.

```
mfirst<- glmmTMB(log(Duration.of.first.drinking) ~ Solution + (1|Colony),
                 family = "gaussian",
                 data = exp1)
mfirstres <- simulateResiduals(mfirst)
checkmodel(mfirstres)
```

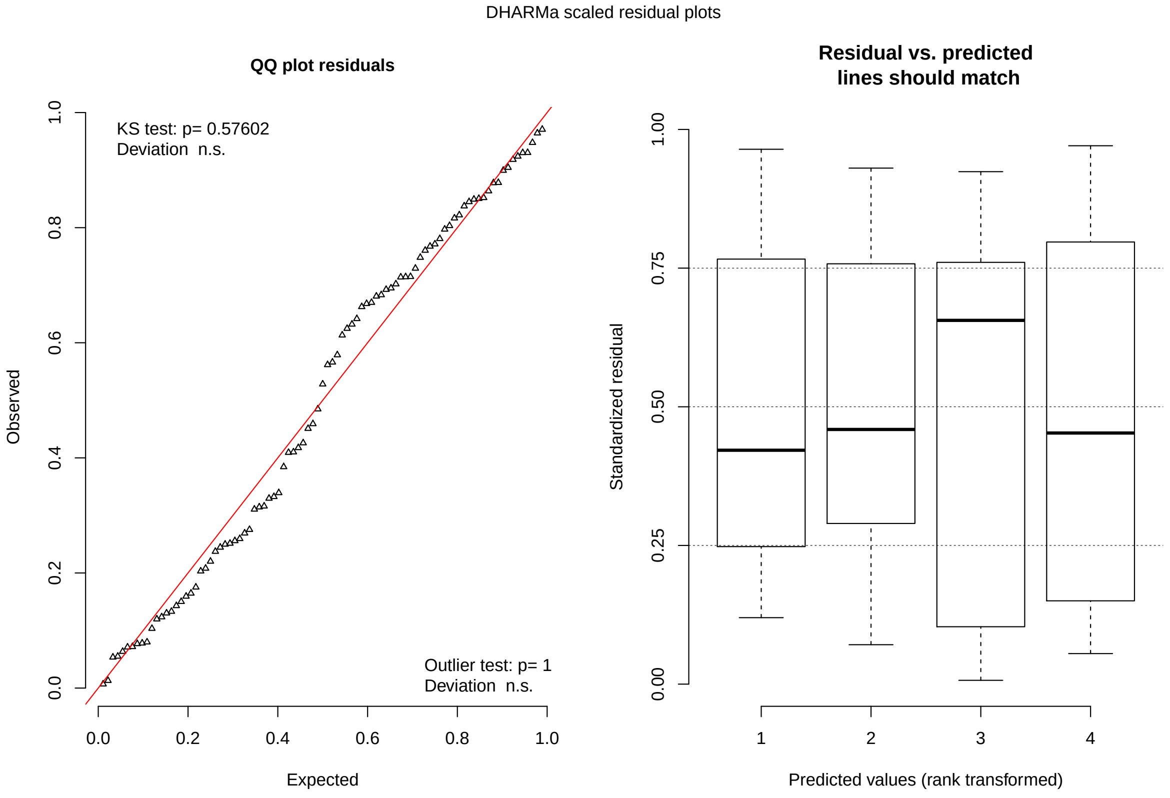

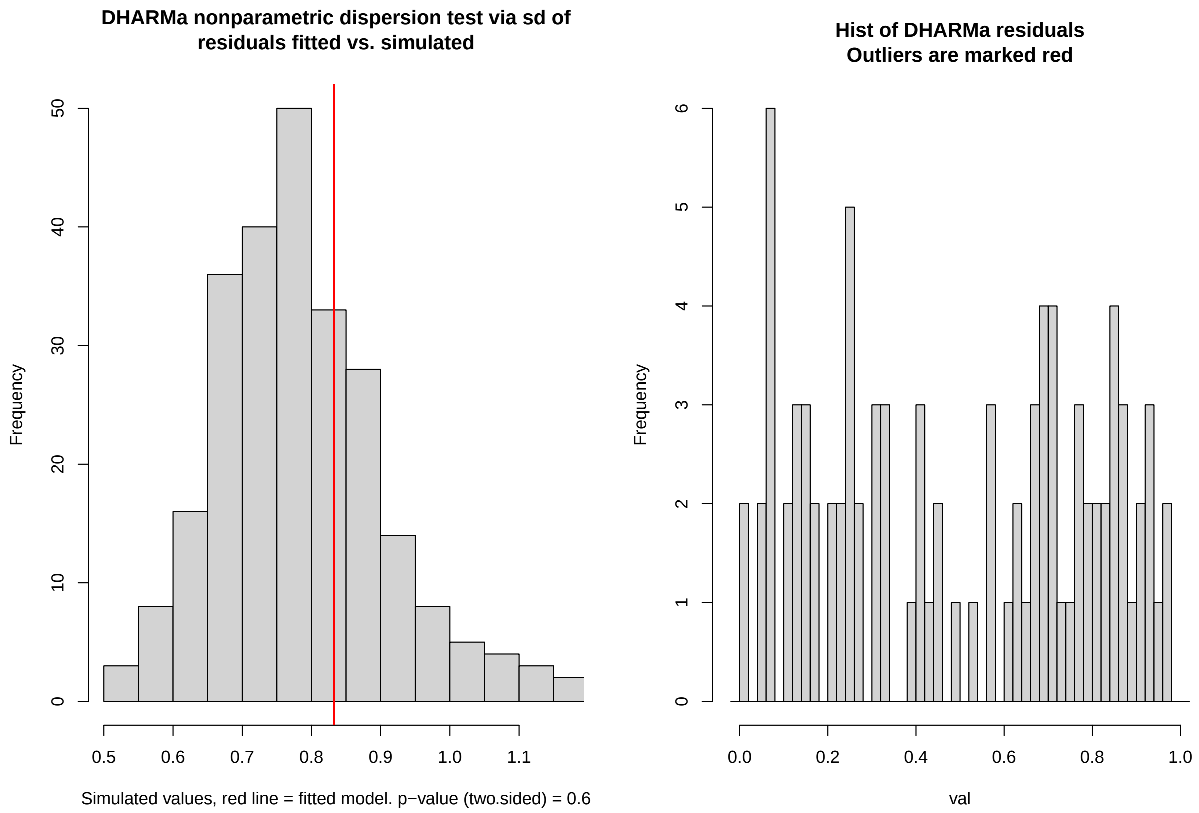


```
## $uniformity
##
##  One-sample Kolmogorov-Smirnov test
##
## data:  simulationOutput$scaledResiduals
## D = 0.080072, p-value = 0.576
## alternative hypothesis: two-sided
##
##
## $dispersion
##
##  DHARMa nonparametric dispersion test via sd of residuals fitted vs.
##  simulated
##
## data:  simulationOutput
## ratioObsSim = 1.0625, p-value = 0.6
## alternative hypothesis: two.sided
##
##
## $outliers
##
##  DHARMa outlier test based on exact binomial test
##
## data:  simulationOutput
## outLow = 0.0000000, outHigh = 0.0000000, nobs = 91.0000000, freqH0 =
## 0.0039841, p-value = 1
## alternative hypothesis: two.sided
```

```r
plot_predictors(exp1, mfirstres, "Solution")
```

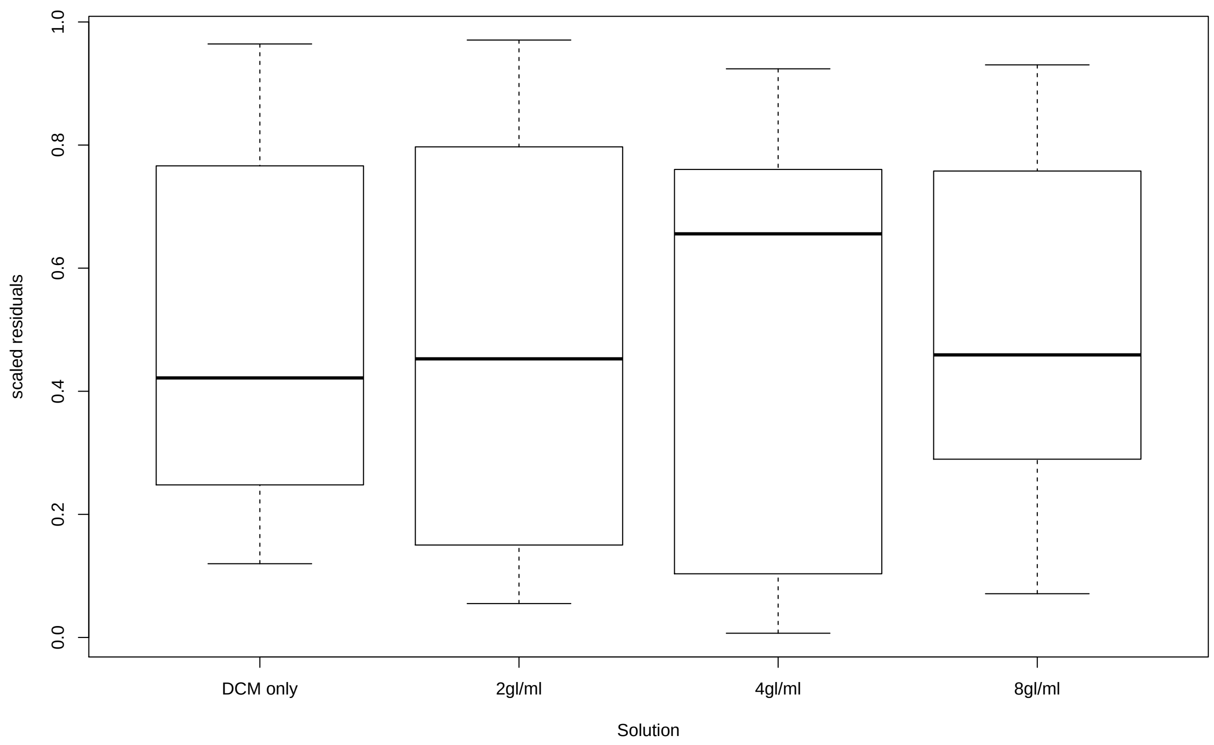

This is better.

Results

```r
Anova(mfirst)
```

```
## Analysis of Deviance Table (Type II Wald chisquare tests)
##
## Response: log(Duration.of.first.drinking)
##           Chisq Df Pr(>Chisq)
## Solution 0.3953  3     0.9412
```

```r
a<-
emmip(mfirst, ~Solution,
      response=T,
      CIs = T,
      type="response")+
  ylab("Predicted duration of first drinking [sec]")+
  xlab("Solution")
a # save for later
```

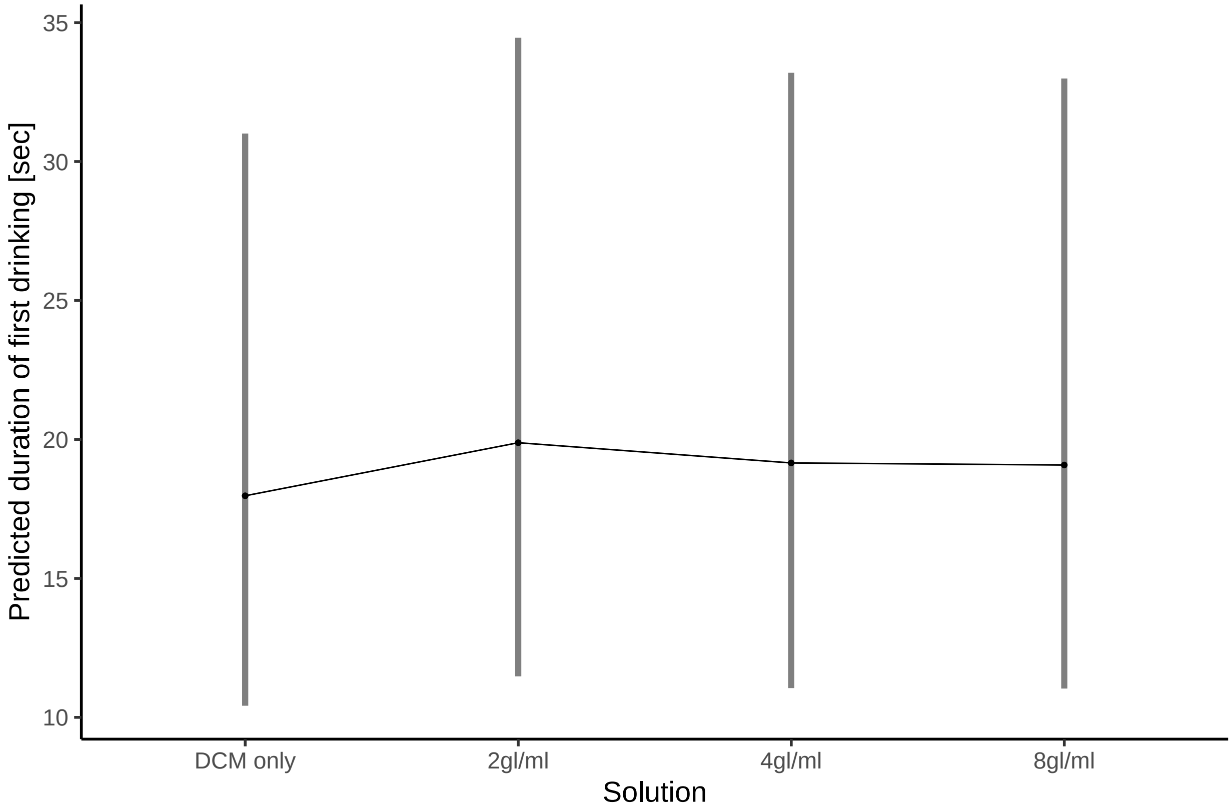

We see large variation, but no systematic effect of pheromone presence.

Total drinking time

```
mtotal<- glmmTMB(log(Time.drinking) ~ Solution + (1|Colony),
                 family = "gaussian",
                 data = exp1)
mtotalres <- simulateResiduals(mtotal)
checkmodel(mtotalres)
```

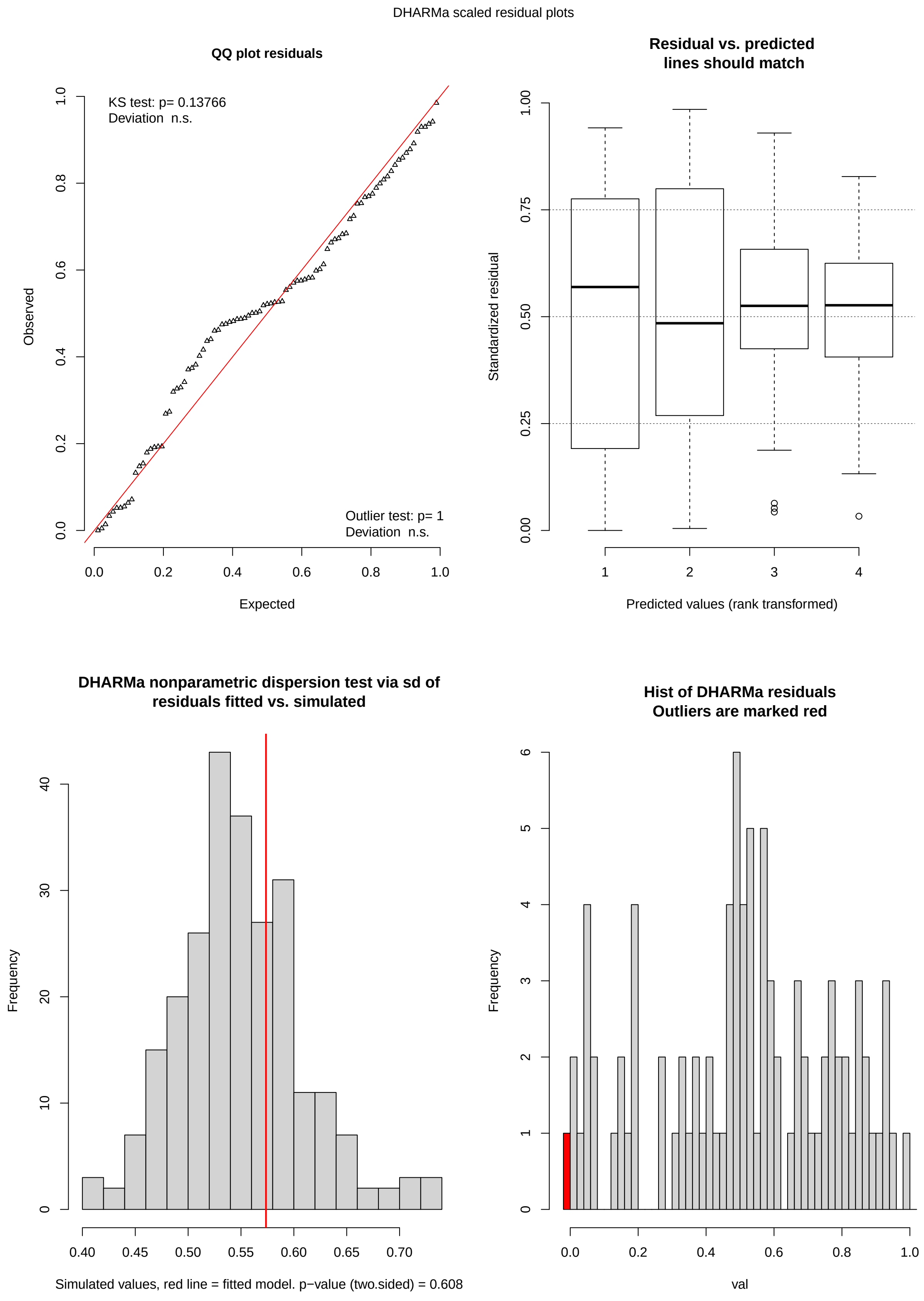
DHARMa scaled residual plots
QQ plot residuals
KS test: p= 0.13766
Deviation n.s.
Outlier test: p= 1
Deviation n.s.
Observed
Expected
Residual vs. predicted
lines should match
Standardized residual
Predicted values (rank transformed)
DHARMa nonparametric dispersion test via sd of
residuals fitted vs. simulated
Frequency
Simulated values, red line = fitted model. p−value (two.sided) = 0.608
Hist of DHARMa residuals
Outliers are marked red
Frequency
val

```
## $uniformity
##
##  One-sample Kolmogorov-Smirnov test
##
## data:  simulationOutput$scaledResiduals
## D = 0.11939, p-value = 0.1377
## alternative hypothesis: two-sided
##
##
## $dispersion
##
##  DHARMa nonparametric dispersion test via sd of residuals fitted vs.
##  simulated
##
## data:  simulationOutput
## ratioObsSim = 1.0435, p-value = 0.608
## alternative hypothesis: two.sided
##
##
## $outliers
##
##  DHARMa outlier test based on exact binomial test
##
## data:  simulationOutput
## outLow = 1.0000000, outHigh = 0.0000000, nobs = 91.0000000, freqH0 =
## 0.0039841, p-value = 1
## alternative hypothesis: two.sided
```

```r
plot_predictors(exp1, mtotalres, "Solution")
```

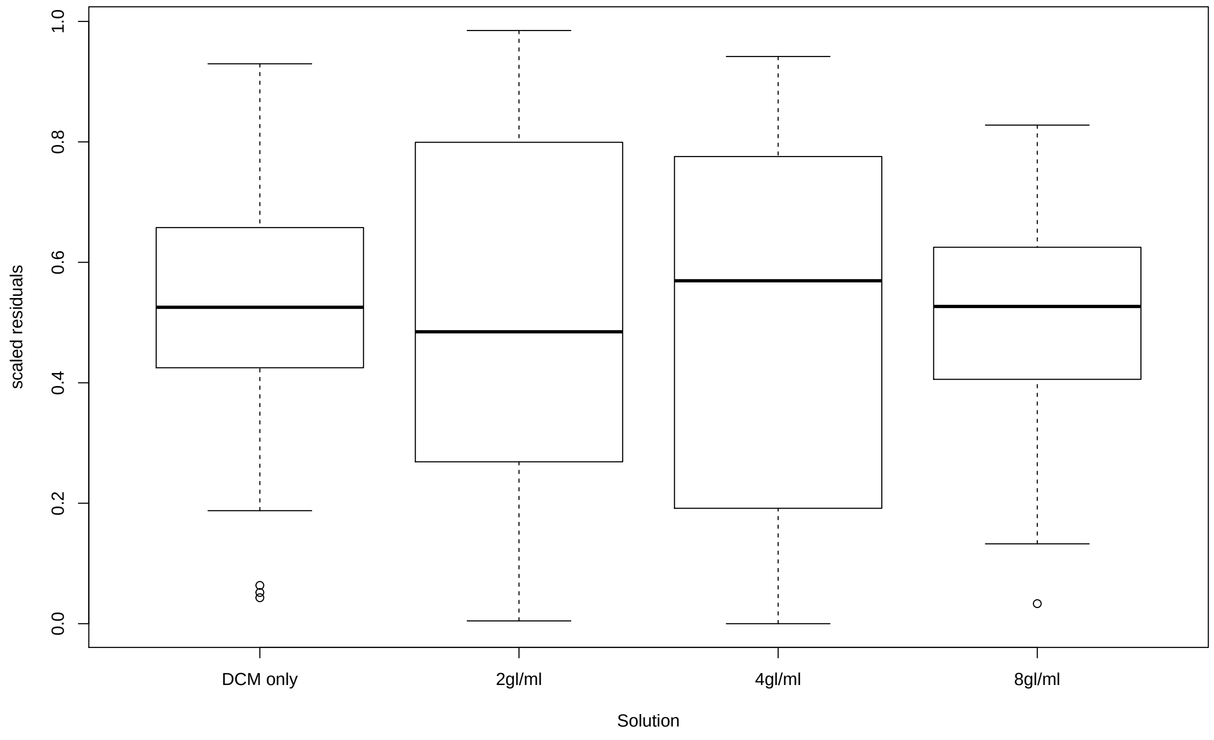

Results

```r
Anova(mtotal)
```

```
## Analysis of Deviance Table (Type II Wald chisquare tests)
##
## Response: log(Time.drinking)
##           Chisq Df Pr(>Chisq)
## Solution 3.0807  3     0.3794
```

```r
emmip(mtotal, ~Solution,
      response=T,
      CIs = T,
      type="response")+
  ylab("Predicted total drinking time [sec]")+
  xlab("Solution")
```

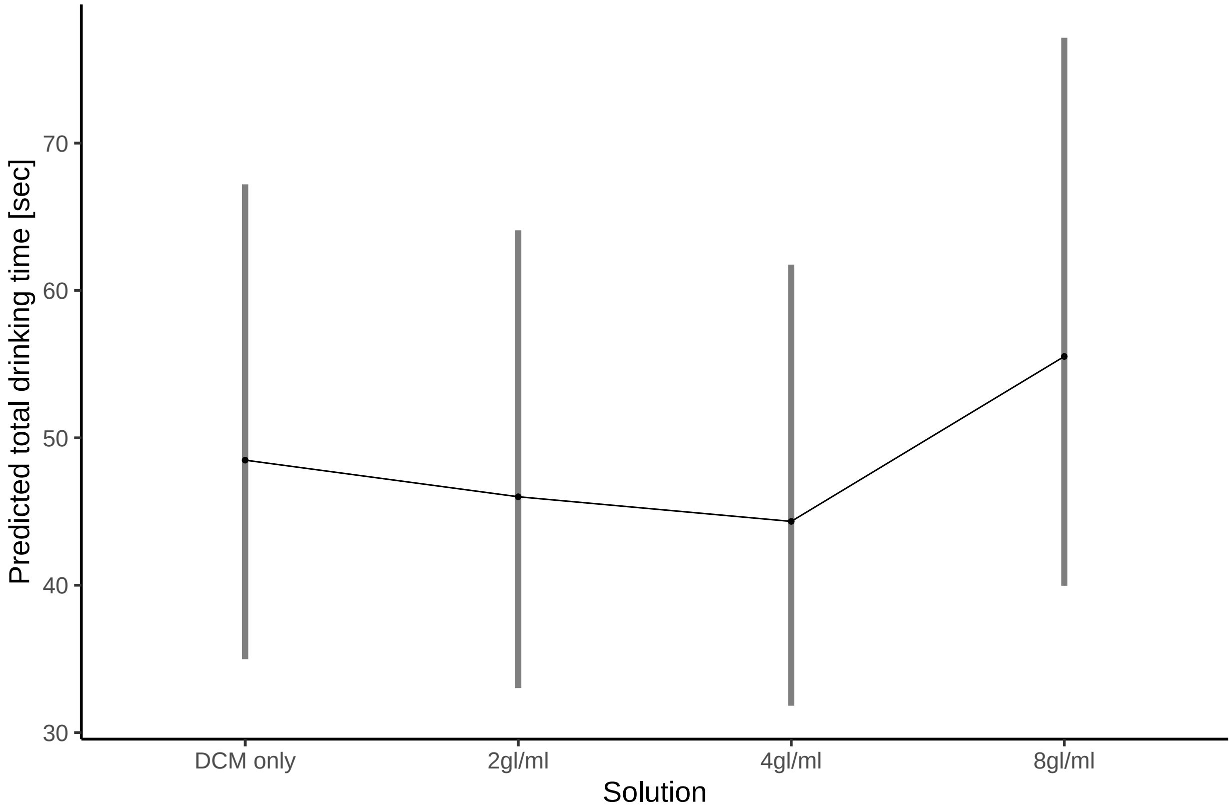

Interruptions

```
minter<- glmmTMB(Drinking.interruptions ~ Solution + (1|Colony),
                 family = "poisson",
                 data = exp1)
minterres <- simulateResiduals(minter)
checkmodel(minterres)
```

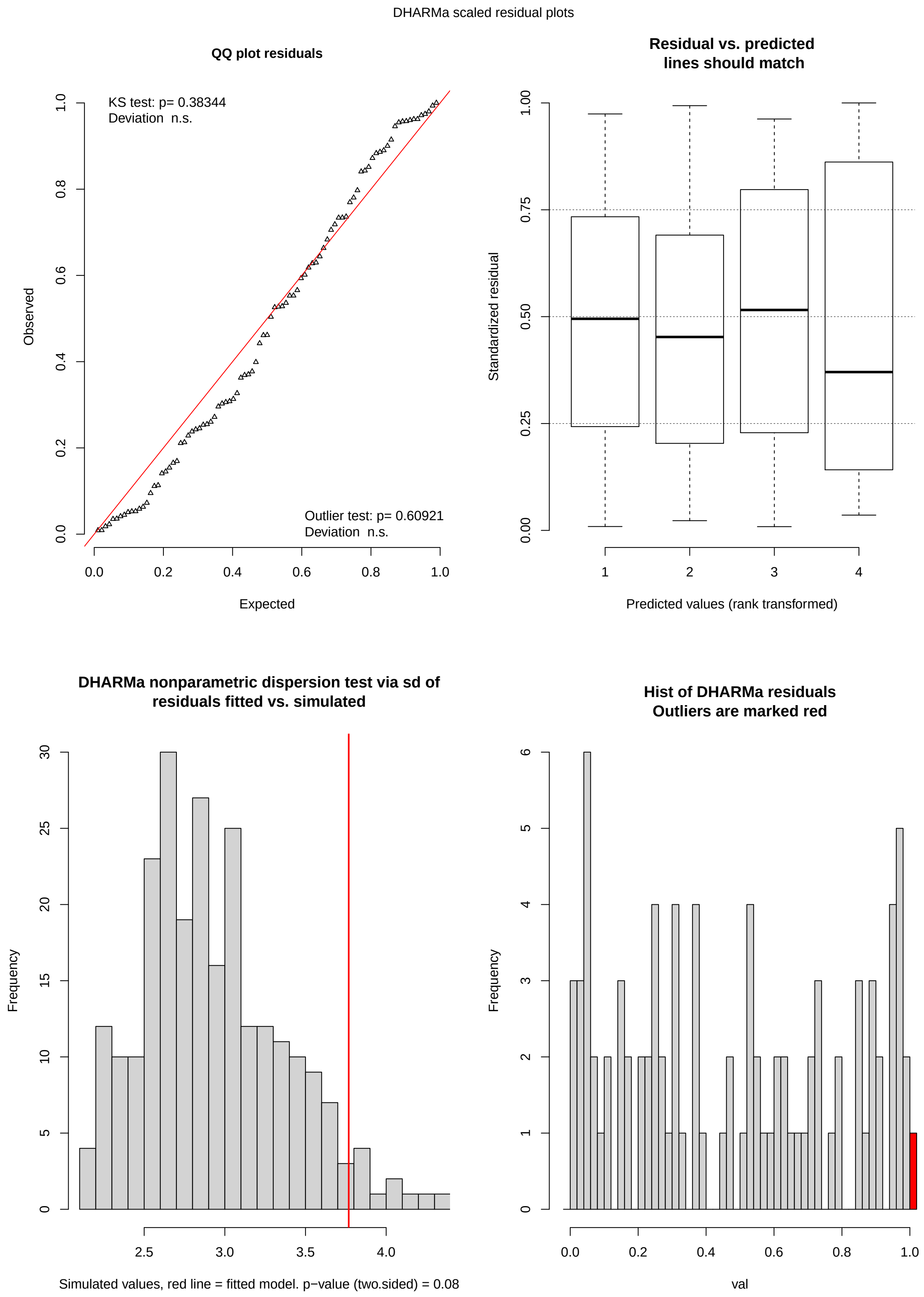
DHARMa scaled residual plots
QQ plot residuals
KS test: p= 0.38344
Deviation n.s.
Outlier test: p= 0.60921
Deviation n.s.
Observed
Expected
Residual vs. predicted
lines should match
Standardized residual
Predicted values (rank transformed)
DHARMa nonparametric dispersion test via sd of
residuals fitted vs. simulated
Frequency
Simulated values, red line = fitted model. p−value (two.sided) = 0.08
Hist of DHARMa residuals
Outliers are marked red
Frequency
val

```
## $uniformity
##
##  One-sample Kolmogorov-Smirnov test
##
## data:  simulationOutput$scaledResiduals
## D = 0.093271, p-value = 0.3834
## alternative hypothesis: two-sided
##
##
## $dispersion
##
##  DHARMa nonparametric dispersion test via sd of residuals fitted vs.
##  simulated
##
## data:  simulationOutput
## ratioObsSim = 1.2867, p-value = 0.08
## alternative hypothesis: two.sided
##
##
## $outliers
##
##  DHARMa outlier test based on exact binomial test
##
## data:  simulationOutput
## outLow = 0.0000000, outHigh = 1.0000000, nobs = 91.0000000, freqH0 =
## 0.0039841, p-value = 0.6092
## alternative hypothesis: two.sided
```

```r
plot_predictors(exp1, minterres, "Solution")
```

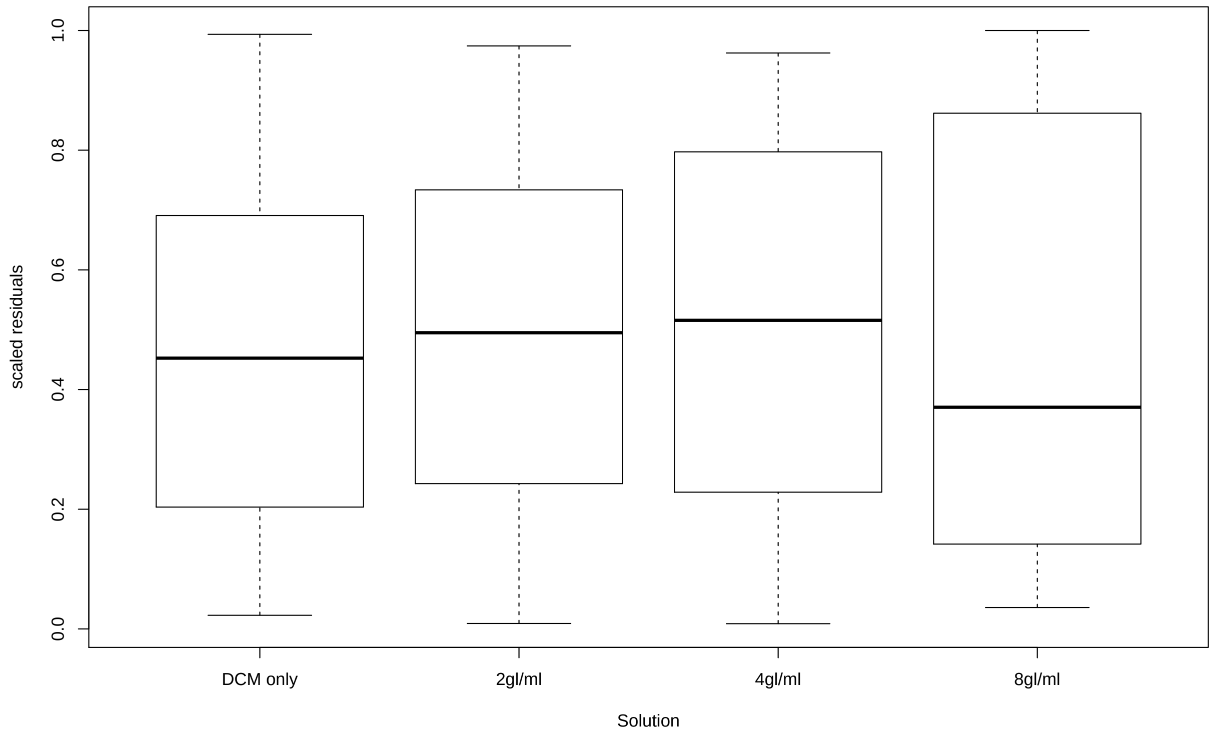

Results

```
Anova(minter)
```

```
## Analysis of Deviance Table (Type II Wald chisquare tests)
##
## Response: Drinking.interruptions
##           Chisq Df Pr(>Chisq)
## Solution 14.727  3   0.002065 **
## ---
## Signif. codes:  0 '***' 0.001 '**' 0.01 '*' 0.05 '.' 0.1 ' ' 1
```

```
b<-
emmip(minter, ~Solution, response=T, CIs = T, type="response")+
  ylab("Predicted number of drinking interruptions")+
  xlab("Solution")
b
```

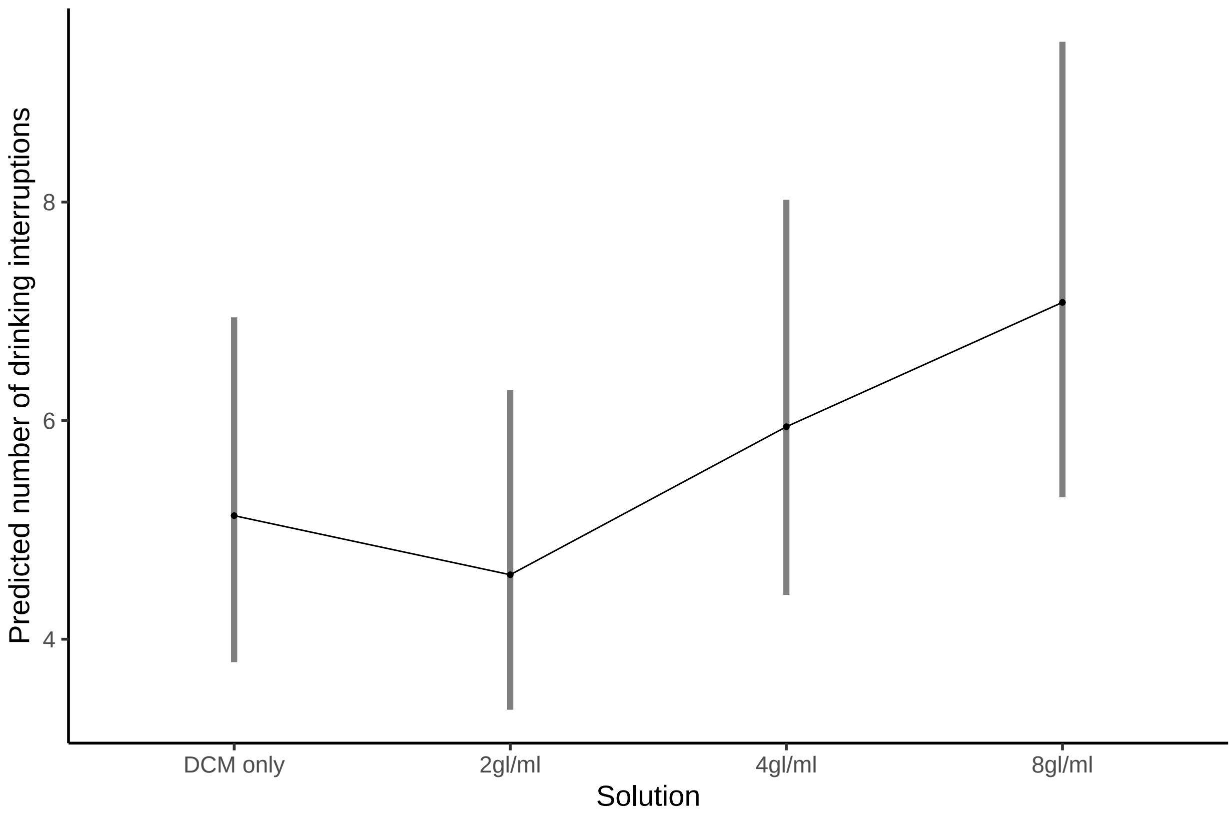


```r
emm <- emmeans(minter, ~Solution)
merge(
contrast(emm, method = "trt.vs.ctrl", type="response", adjust="mvt"),
confint(contrast(emm, method = "trt.vs.ctrl", type="response", adjust="mvt"))[c(1,5,6)],
by = "contrast")
```

```
##             contrast     ratio        SE df    t.ratio    p.value lower.CL
## 1 2gl/ml / DCM only 0.8946415 0.1170682 86 -0.8508068 0.72584764 0.654576
## 2 4gl/ml / DCM only 1.1586045 0.1415209 86  1.2052319 0.48265190 0.865516
## 3 8gl/ml / DCM only 1.3803409 0.1606472 86  2.7695836 0.01857837 1.045453
##   upper.CL
## 1 1.222751
## 2 1.550941
## 3 1.822502
```

U-turns to food

```r
exp1 %>%
  mutate(turned = ifelse(U.turns.to.food > 0, "yes", "no")) %>%
  group_by(turned, Solution) %>%
  tally %>%
  create_table()
```

turned	Solution	n
no	DCM only	11
no	2gl/ml	5
no	4gl/ml	4
no	8gl/ml	5

turned	Solution	n
yes	DCM only	13
yes	2gl/ml	17
yes	4gl/ml	18
yes	8gl/ml	18

```
mturns<- glmmTMB(U.turns.to.food ~ Solution + (1|Colony),
                 family = "poisson",
                 data = exp1)
mturnsres <- simulateResiduals(mturns)
checkmodel(mturnsres)
```

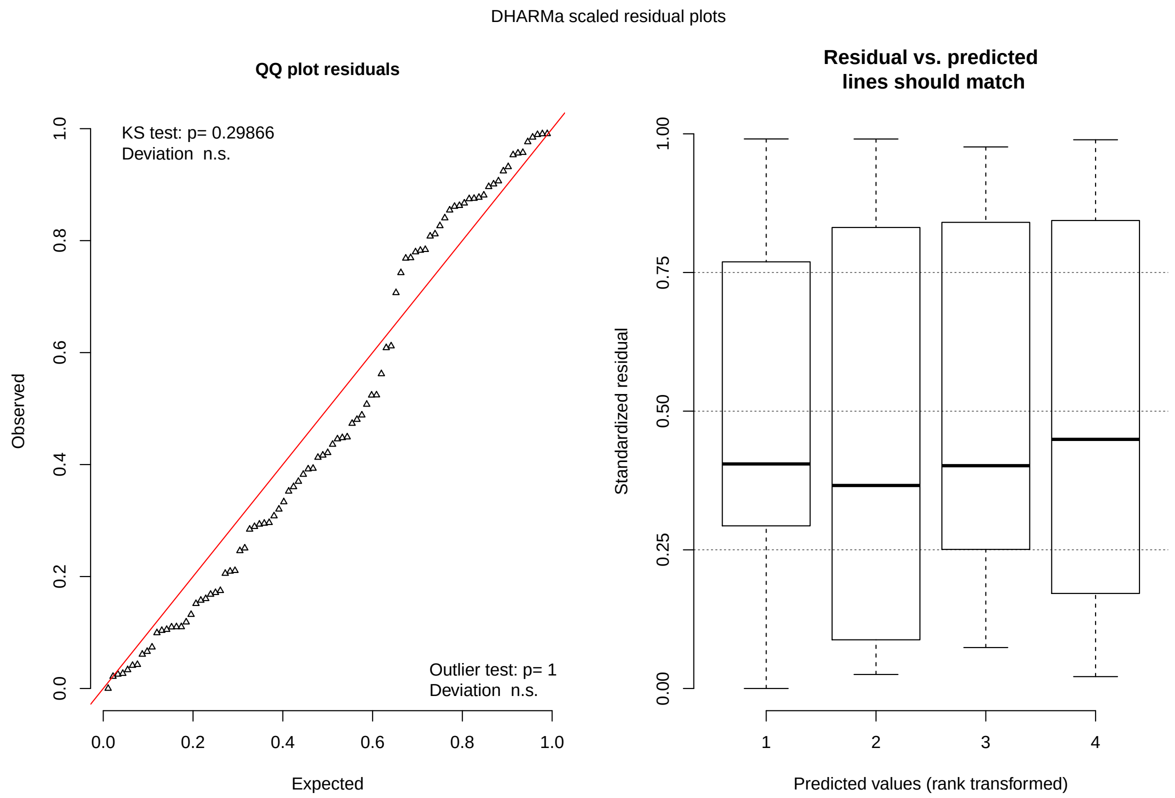

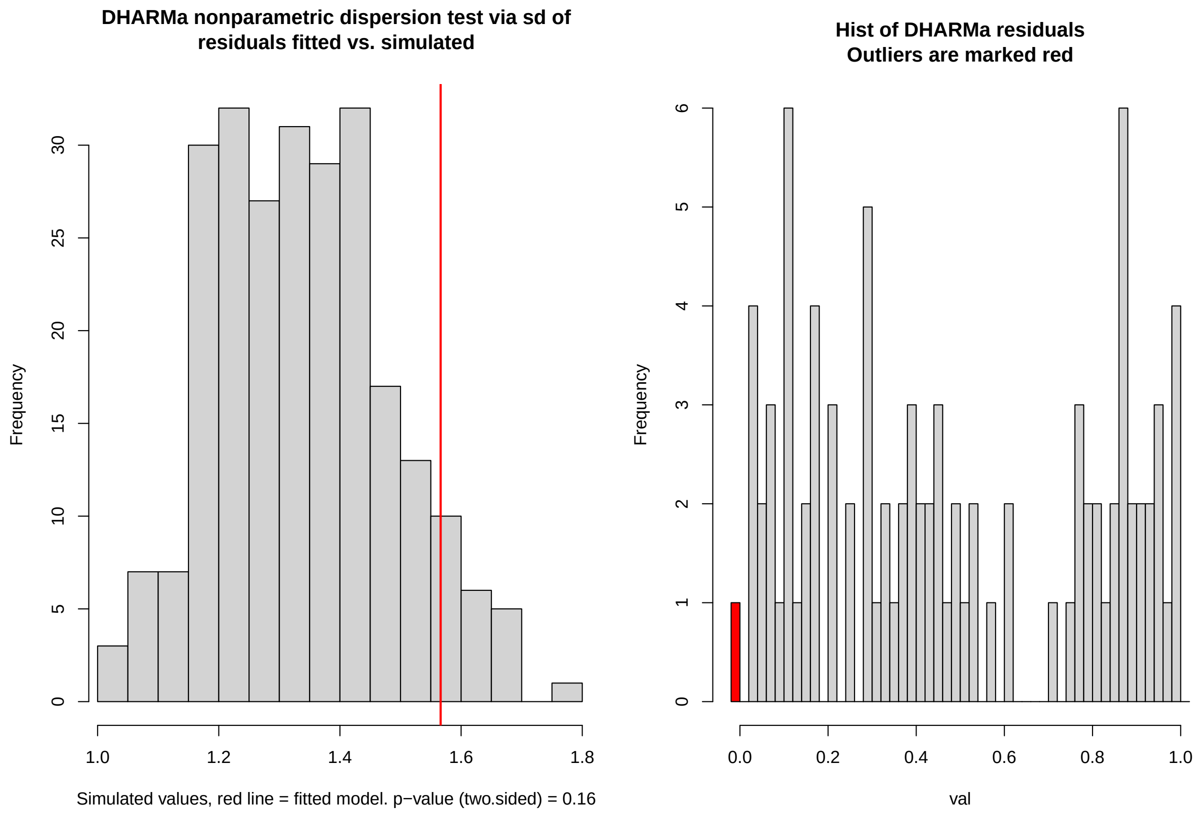


```
## $uniformity
##
##  One-sample Kolmogorov-Smirnov test
##
## data:  simulationOutput$scaledResiduals
## D = 0.10032, p-value = 0.2987
## alternative hypothesis: two-sided
##
##
## $dispersion
##
##  DHARMa nonparametric dispersion test via sd of residuals fitted vs.
##  simulated
##
## data:  simulationOutput
## ratioObsSim = 1.1714, p-value = 0.16
## alternative hypothesis: two.sided
##
##
## $outliers
##
##  DHARMa outlier test based on exact binomial test
##
## data:  simulationOutput
## outLow = 1.0000000, outHigh = 0.0000000, nobs = 91.0000000, freqH0 =
## 0.0039841, p-value = 1
## alternative hypothesis: two.sided
```

```r
plot_predictors(exp1, mturnsres, "Solution")
```

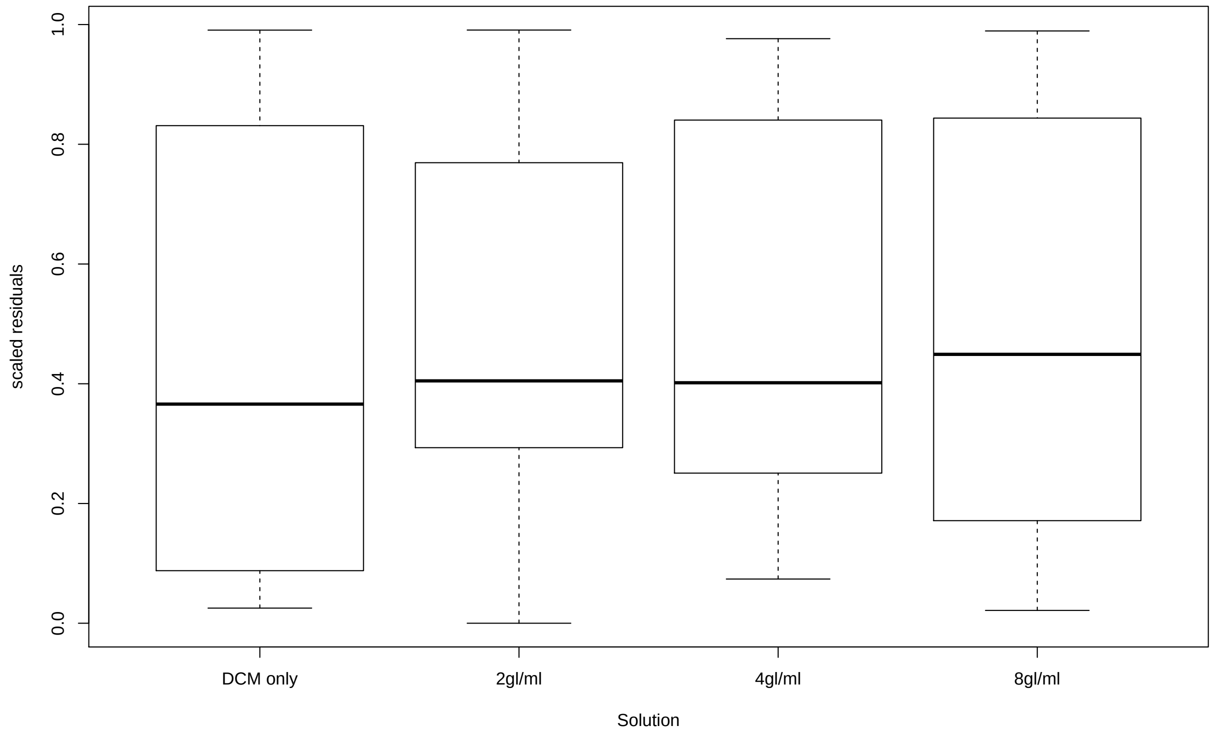

Results

```r
Anova(mturns)
```

```
## Analysis of Deviance Table (Type II Wald chisquare tests)
##
## Response: U.turns.to.food
##           Chisq Df Pr(>Chisq)
## Solution 4.1791  3     0.2428
```

```r
emmip(mturns, ~Solution, response=T, CIs = T, type="response")+
  ylab("Predicted number of u-turns to food")+
  xlab("Solution")
```

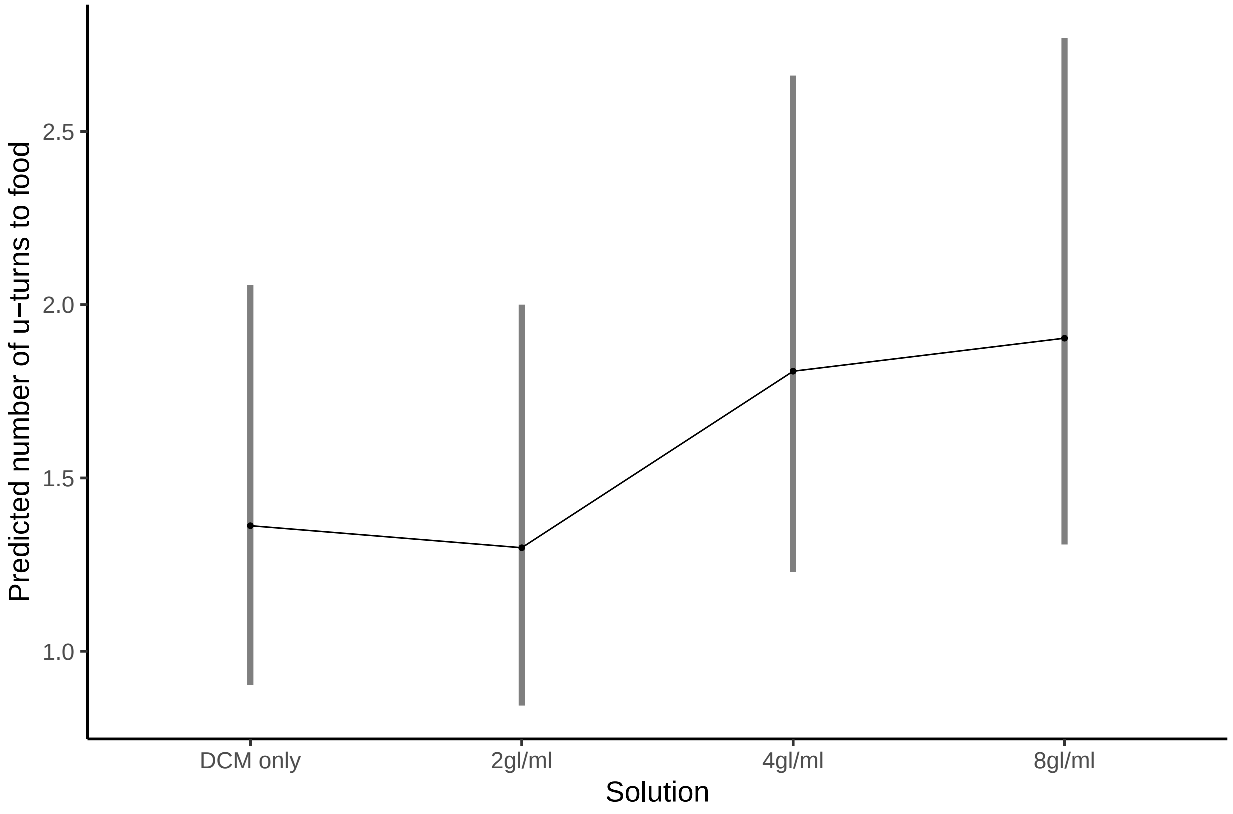

U-turns to nest

```
exp1 %>%
  mutate(turned = ifelse(U.turns.to.nest > 0, "yes", "no")) %>%
  group_by(turned, Solution) %>%
  tally %>%
  create_table()
```

turned	Solution	n
no	DCM only	18
no	2gl/ml	18
no	4gl/ml	18
no	8gl/ml	21
yes	DCM only	6
yes	2gl/ml	4
yes	4gl/ml	4
yes	8gl/ml	2

```
mturnsnest<- glmmTMB(U.turns.to.nest ~ Solution + (1|Colony),
                    family = "poisson",
                    data = exp1)
mturnsnestres <- simulateResiduals(mturnsnest)
checkmodel(mturnsnestres)
```

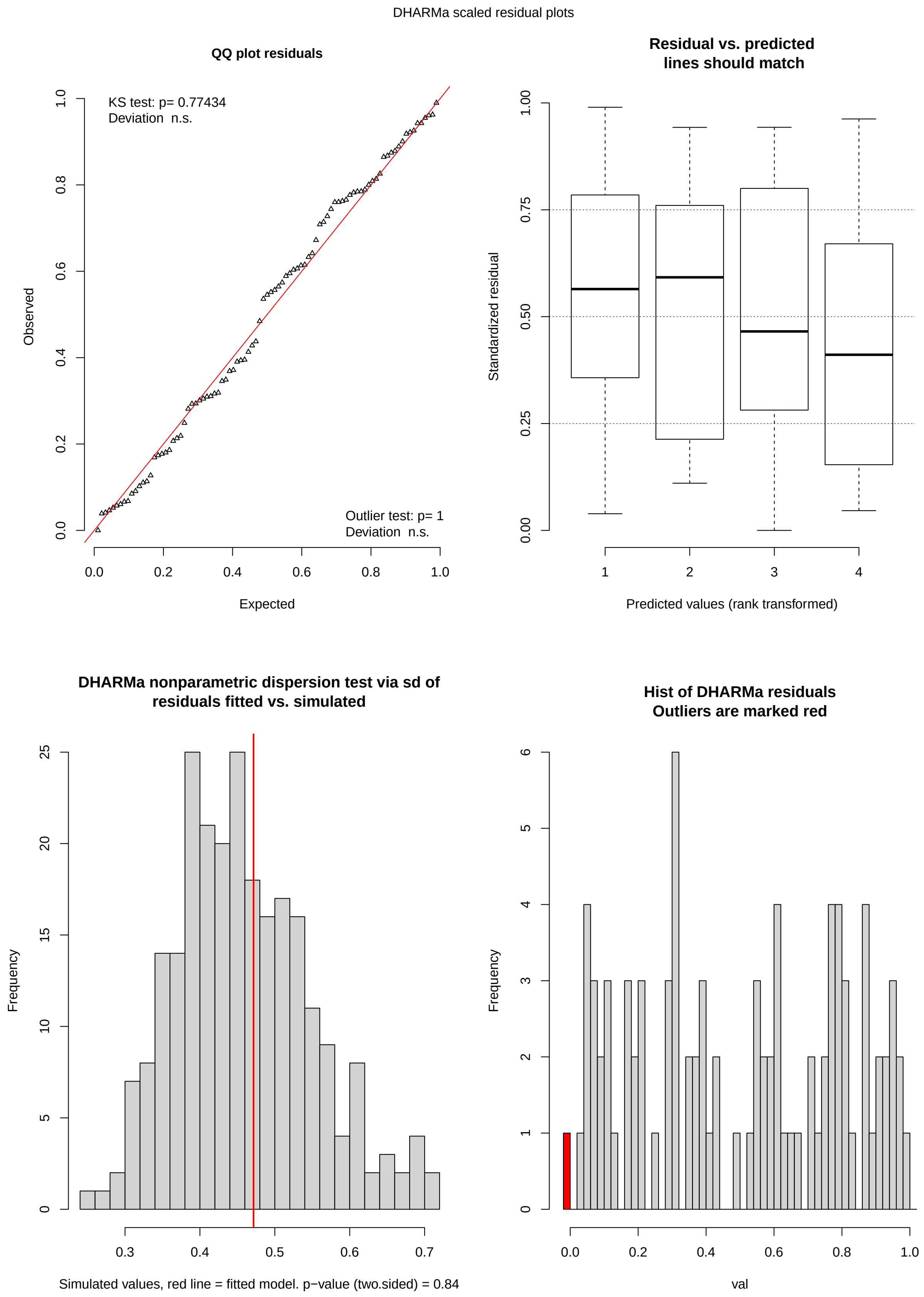
DHARMa scaled residual plots
QQ plot residuals
KS test: p= 0.77434
Deviation n.s.
Outlier test: p= 1
Deviation n.s.
Observed
Expected
Residual vs. predicted
lines should match
Standardized residual
Predicted values (rank transformed)
DHARMa nonparametric dispersion test via sd of
residuals fitted vs. simulated
Frequency
Simulated values, red line = fitted model. p−value (two.sided) = 0.84
Hist of DHARMa residuals
Outliers are marked red
Frequency
val

```
## $uniformity
##
##  One-sample Kolmogorov-Smirnov test
##
## data:  simulationOutput$scaledResiduals
## D = 0.067593, p-value = 0.7743
## alternative hypothesis: two-sided
##
##
## $dispersion
##
##  DHARMa nonparametric dispersion test via sd of residuals fitted vs.
##  simulated
##
## data:  simulationOutput
## ratioObsSim = 1.0281, p-value = 0.84
## alternative hypothesis: two.sided
##
##
## $outliers
##
##  DHARMa outlier test based on exact binomial test
##
## data:  simulationOutput
## outLow = 1.0000000, outHigh = 0.0000000, nobs = 91.0000000, freqH0 =
## 0.0039841, p-value = 1
## alternative hypothesis: two.sided
```

```
plot_predictors(exp1, mturnsnestres, "Solution")
```

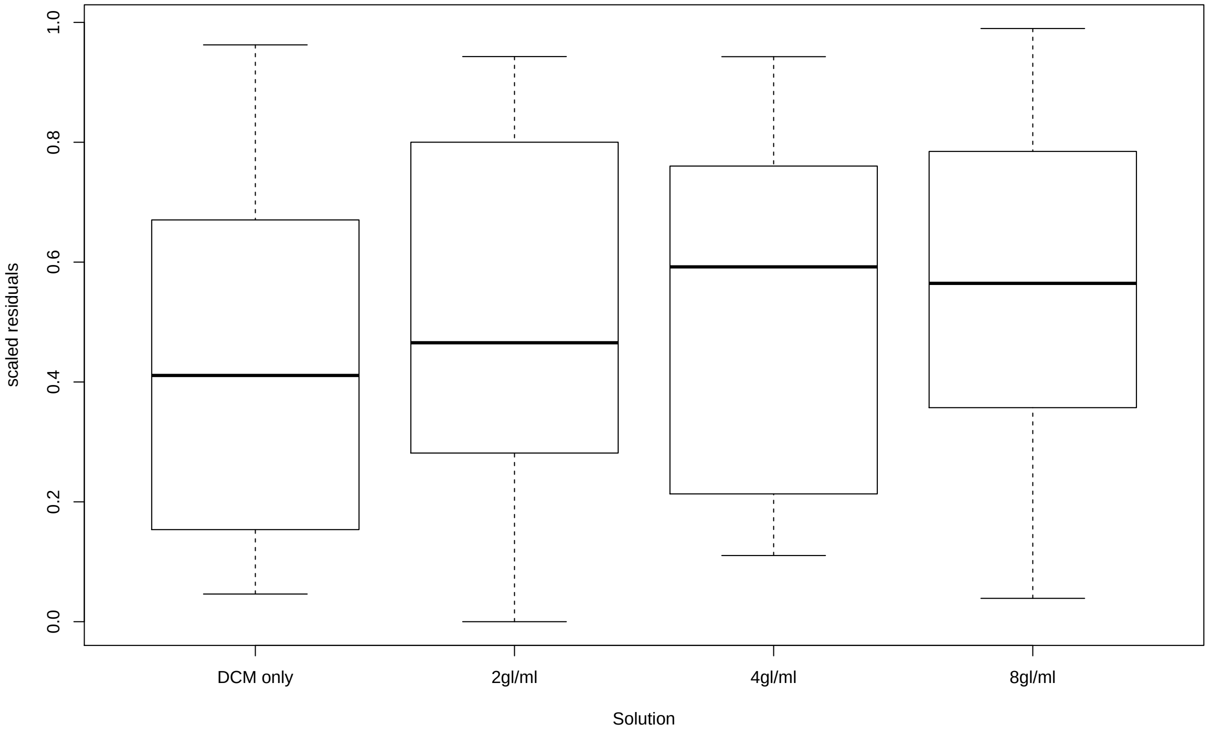

Results

```
Anova(mturnsnest)
```

```
## Analysis of Deviance Table (Type II Wald chisquare tests)
##
## Response: U.turns.to.nest
##           Chisq Df Pr(>Chisq)
## Solution 4.5154  3     0.2109
```

```
emmip(mturnsnest, ~Solution, response=T, CIs = T, type="response")+
  ylab("Predicted number of u-turns to nest")+
  xlab("Solution")
```

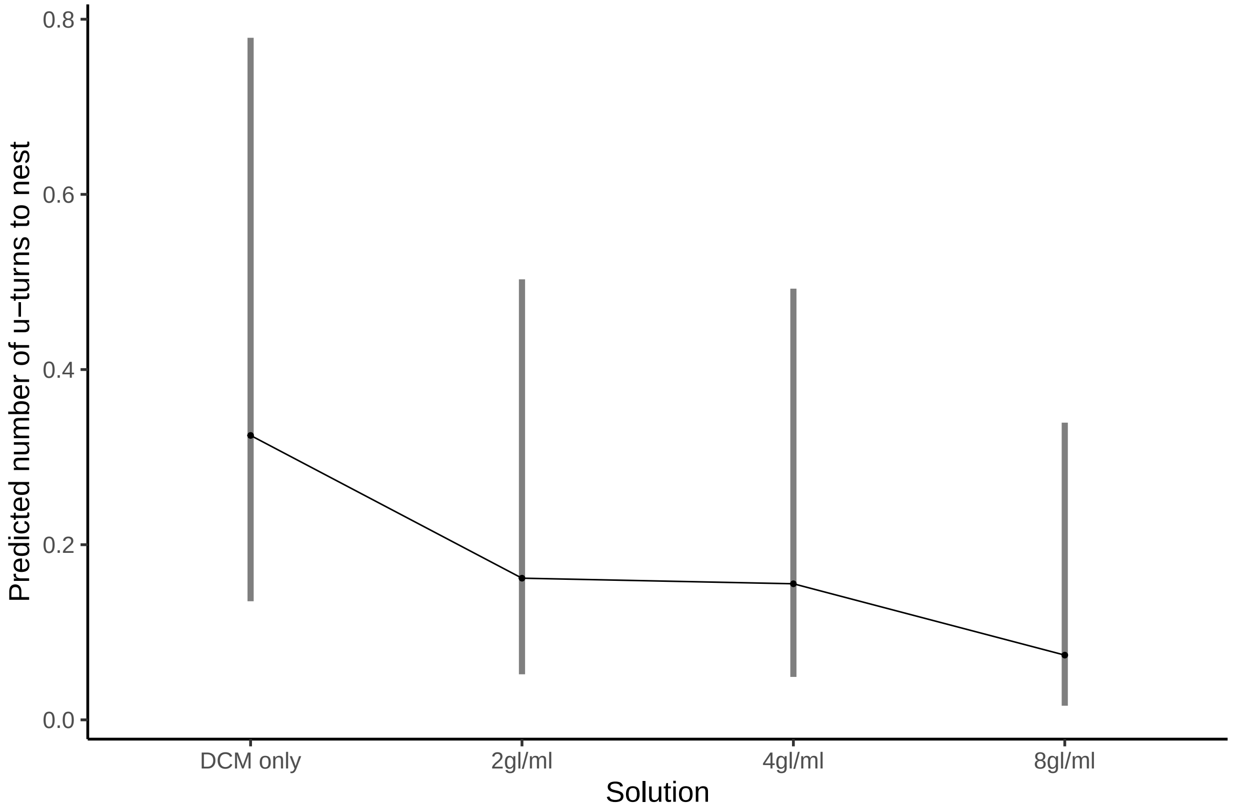

Duration to food

```
m1tofood<-glmmTMB(log(Time.spent.to.food) ~ Solution + (1|Colony),
                  data = exp1,
                  family = "gaussian")

m1tofoodres<-simulateResiduals(m1tofood)
checkmodel(m1tofoodres)
```

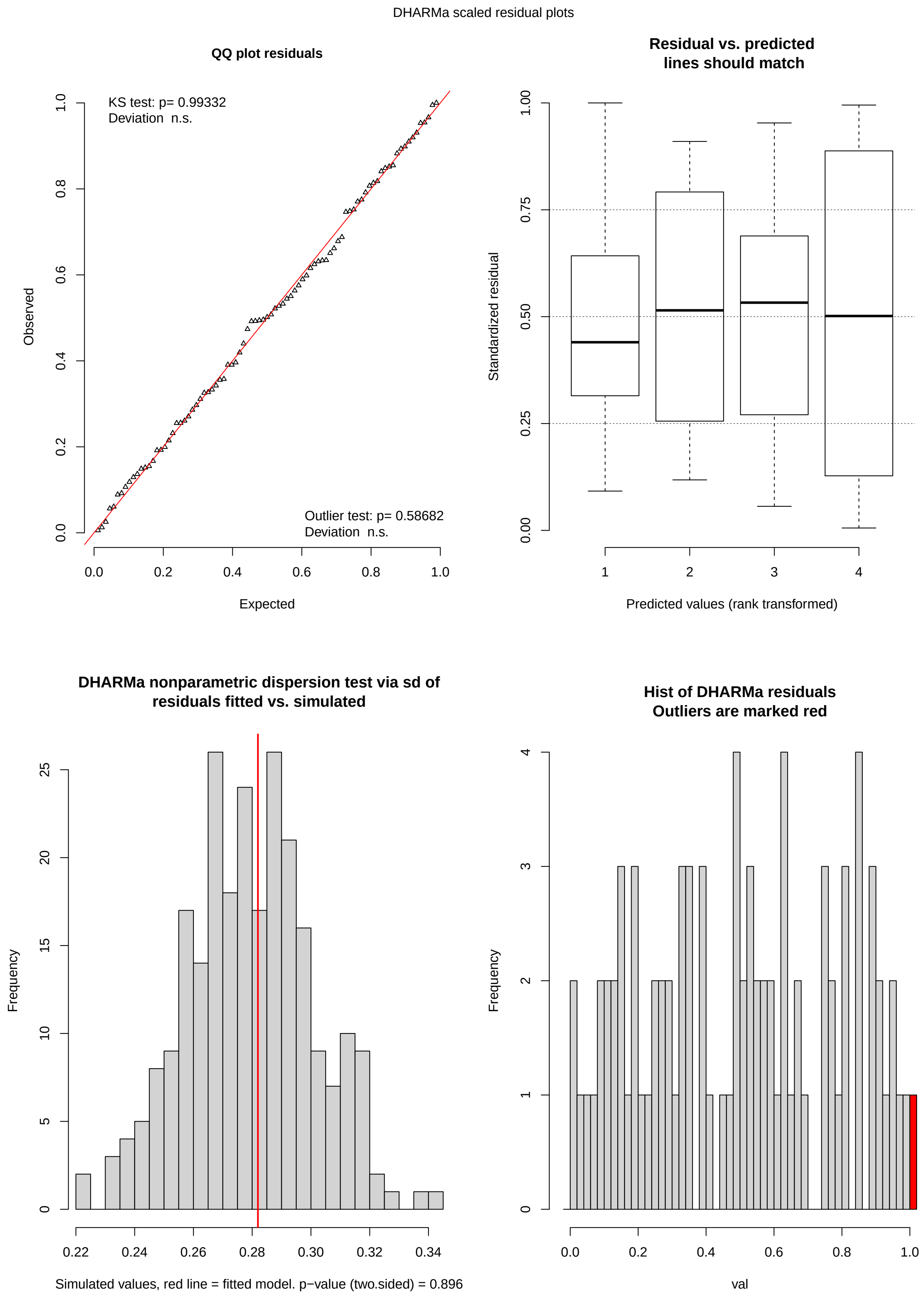
DHARMa scaled residual plots
QQ plot residuals
KS test: p= 0.99332
Deviation n.s.
Outlier test: p= 0.58682
Deviation n.s.
Observed
Expected
Residual vs. predicted
lines should match
Standardized residual
Predicted values (rank transformed)
DHARMa nonparametric dispersion test via sd of
residuals fitted vs. simulated
Frequency
Simulated values, red line = fitted model. p−value (two.sided) = 0.896
Hist of DHARMa residuals
Outliers are marked red
Frequency
val

```
## $uniformity
##
##  One-sample Kolmogorov-Smirnov test
##
## data:  simulationOutput$scaledResiduals
## D = 0.044006, p-value = 0.9933
## alternative hypothesis: two-sided
##
##
## $dispersion
##
##  DHARMa nonparametric dispersion test via sd of residuals fitted vs.
##  simulated
##
## data:  simulationOutput
## ratioObsSim = 1.0092, p-value = 0.896
## alternative hypothesis: two.sided
##
##
## $outliers
##
##  DHARMa outlier test based on exact binomial test
##
## data:  simulationOutput
## outLow = 0.0000000, outHigh = 1.0000000, nobs = 87.0000000, freqH0 =
## 0.0039841, p-value = 0.5868
## alternative hypothesis: two.sided
```

```
plot_predictors(na.omit(exp1), m1tofoodres, "Solution") #4 ants have NAs
```

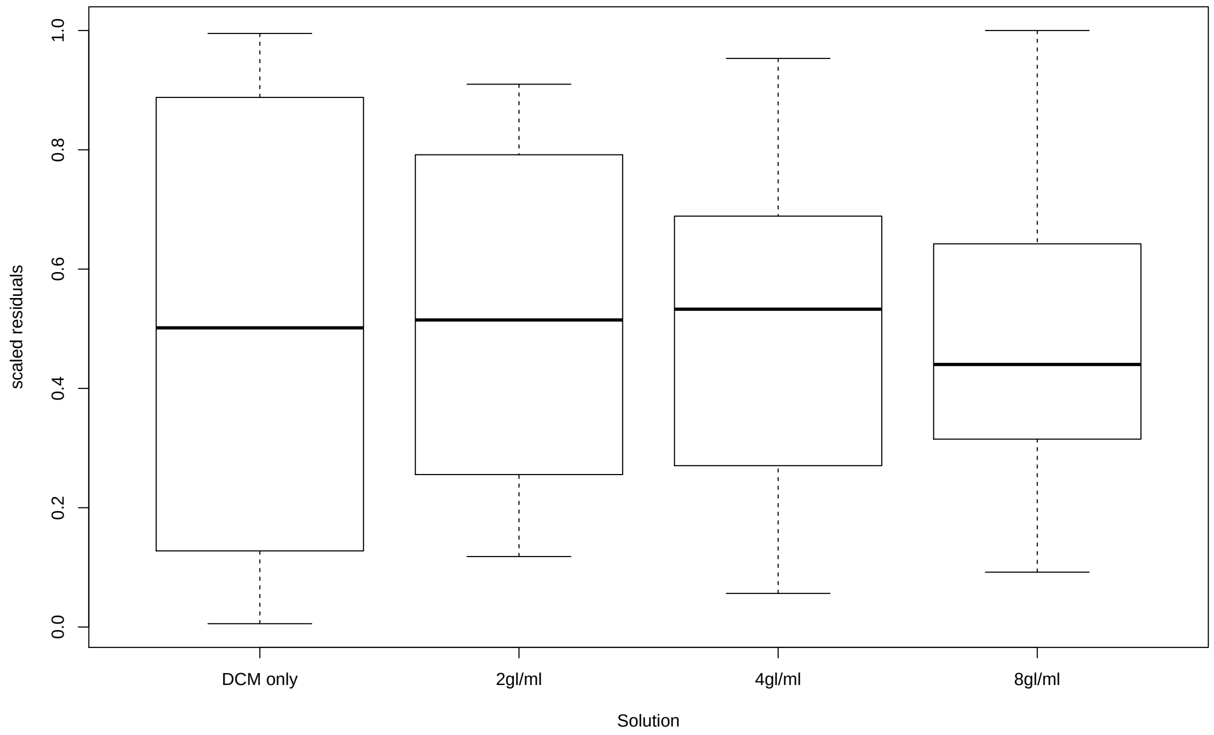

Results

```
Anova(m1tofood)
```

```
## Analysis of Deviance Table (Type II Wald chisquare tests)
##
## Response: log(Time.spent.to.food)
##           Chisq Df Pr(>Chisq)
## Solution 32.342  3  4.432e-07 ***
## ---
## Signif. codes:  0 '***' 0.001 '**' 0.01 '*' 0.05 '.' 0.1 ' ' 1
```

```
c<-
emmip(m1tofood, ~Solution,
      response=T,
      CIs = T,
      type="response")+
  ylab("Predicted time to food [sec]")+
  xlab("Solution")
c
```

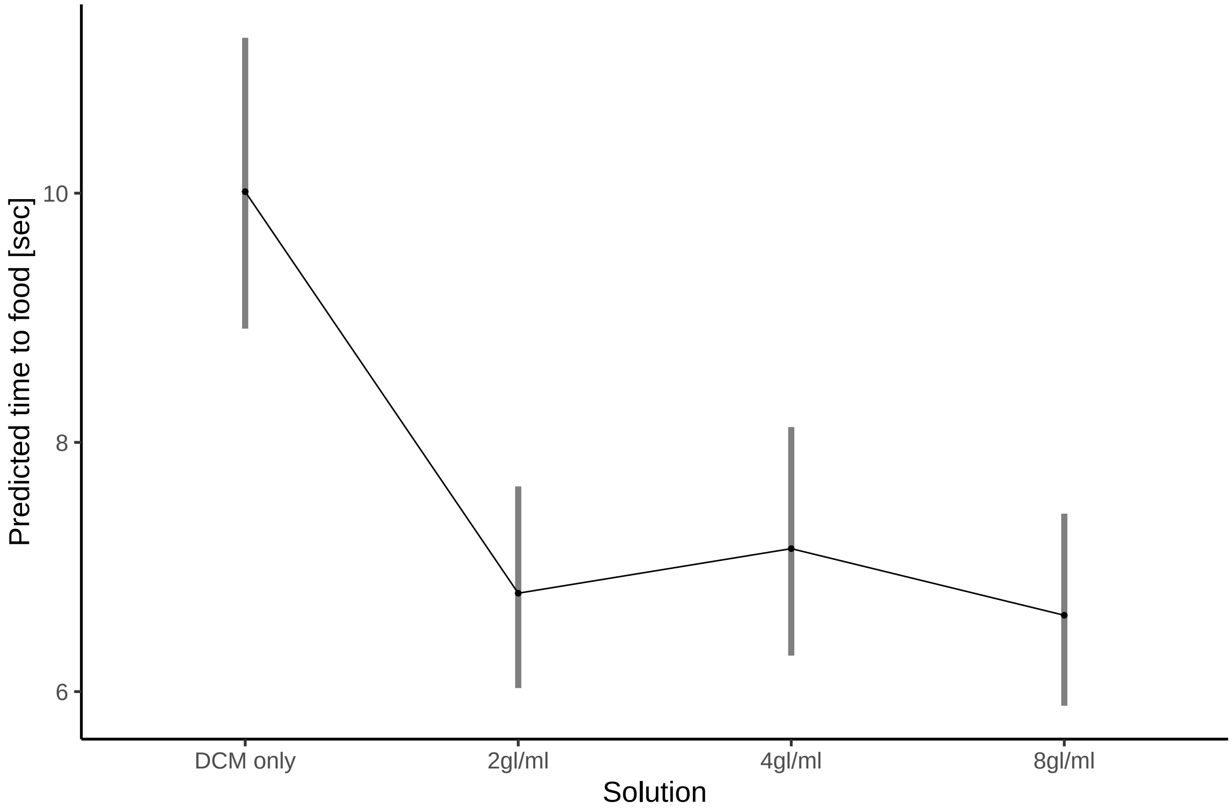


```
emm <- emmeans(m1tofood, ~Solution)
merge(
contrast(emm, method = "trt.vs.ctrl", type="response", adjust="mvt"),
confint(contrast(emm, method = "trt.vs.ctrl", type="response", adjust="mvt"))[c(1,5,6)],
by = "contrast")
```

```
##            contrast     ratio         SE df   t.ratio      p.value  lower.CL
## 1 2gl/ml / DCM only 0.6781313 0.05669051 81 -4.646208 3.777105e-05 0.5549688
## 2 4gl/ml / DCM only 0.7138624 0.06203889 81 -3.878503 5.792118e-04 0.5795956
## 3 8gl/ml / DCM only 0.6604531 0.05459572 81 -5.018255 7.737299e-06 0.5417130
##    upper.CL
## 1 0.8286270
## 2 0.8792331
## 3 0.8052201
```

Total time on setup

```
mtotalsetup<- glmmTMB(log(Total.time.on.setup) ~ Solution + (1|Colony),
                      family = "gaussian", data = exp1)
mtotalsetupres <- simulateResiduals(mtotalsetup)
checkmodel(mtotalsetupres)
```

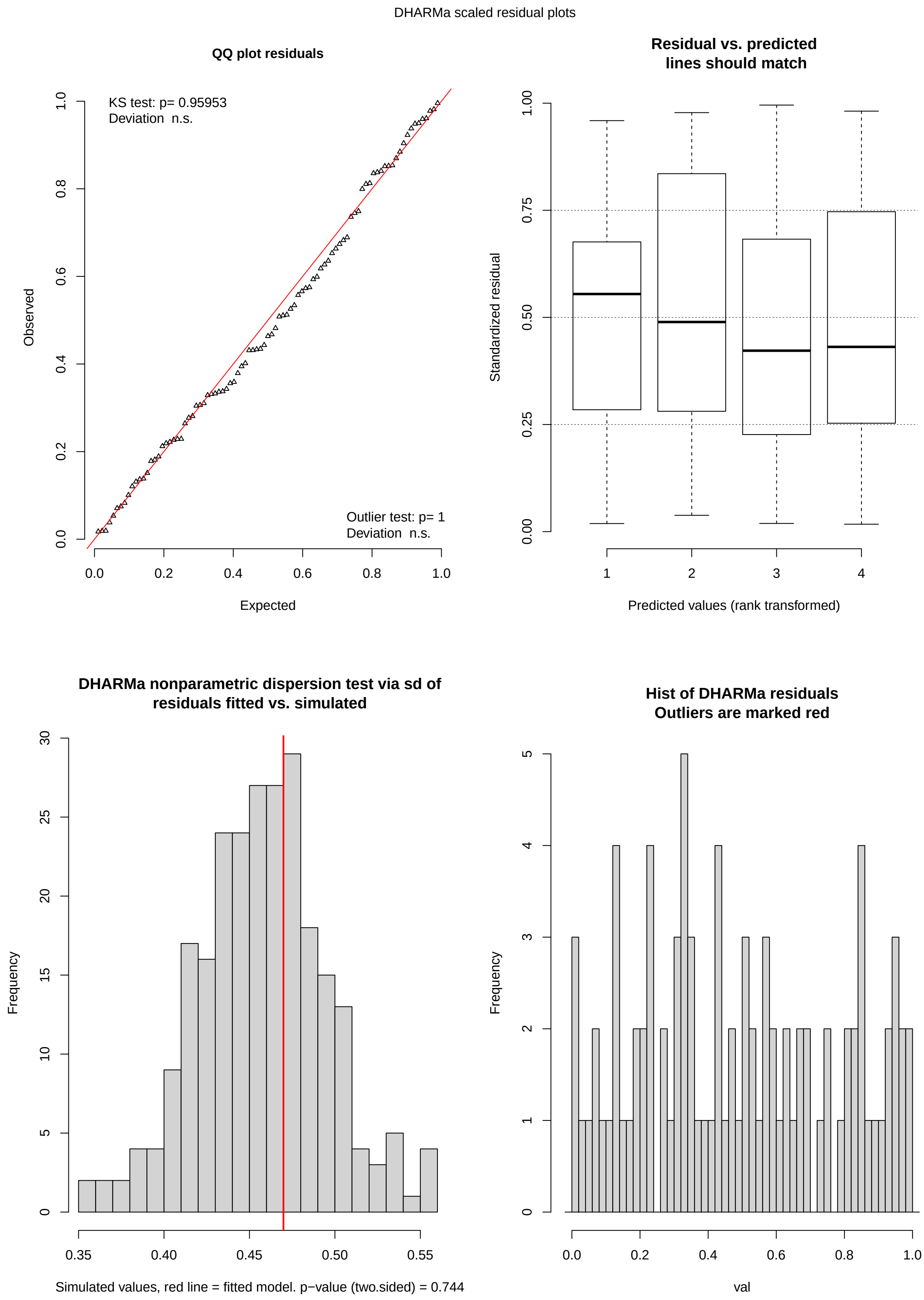
DHARMa scaled residual plots
QQ plot residuals
KS test: p= 0.95953
Deviation n.s.
Outlier test: p= 1
Deviation n.s.
Observed
Expected
Residual vs. predicted
lines should match
Standardized residual
Predicted values (rank transformed)
DHARMa nonparametric dispersion test via sd of
residuals fitted vs. simulated
Frequency
Simulated values, red line = fitted model. p−value (two.sided) = 0.744
Hist of DHARMa residuals
Outliers are marked red
Frequency
val

```
## $uniformity
##
##  One-sample Kolmogorov-Smirnov test
##
## data:  simulationOutput$scaledResiduals
## D = 0.051426, p-value = 0.9595
## alternative hypothesis: two-sided
##
##
## $dispersion
##
##  DHARMa nonparametric dispersion test via sd of residuals fitted vs.
##  simulated
##
## data:  simulationOutput
## ratioObsSim = 1.0281, p-value = 0.744
## alternative hypothesis: two.sided
##
##
## $outliers
##
##  DHARMa outlier test based on exact binomial test
##
## data:  simulationOutput
## outLow = 0.0000000, outHigh = 0.0000000, nobs = 91.0000000, freqH0 =
## 0.0039841, p-value = 1
## alternative hypothesis: two.sided
```

```
plot_predictors(exp1, mtotalsetupres, "Solution")
```

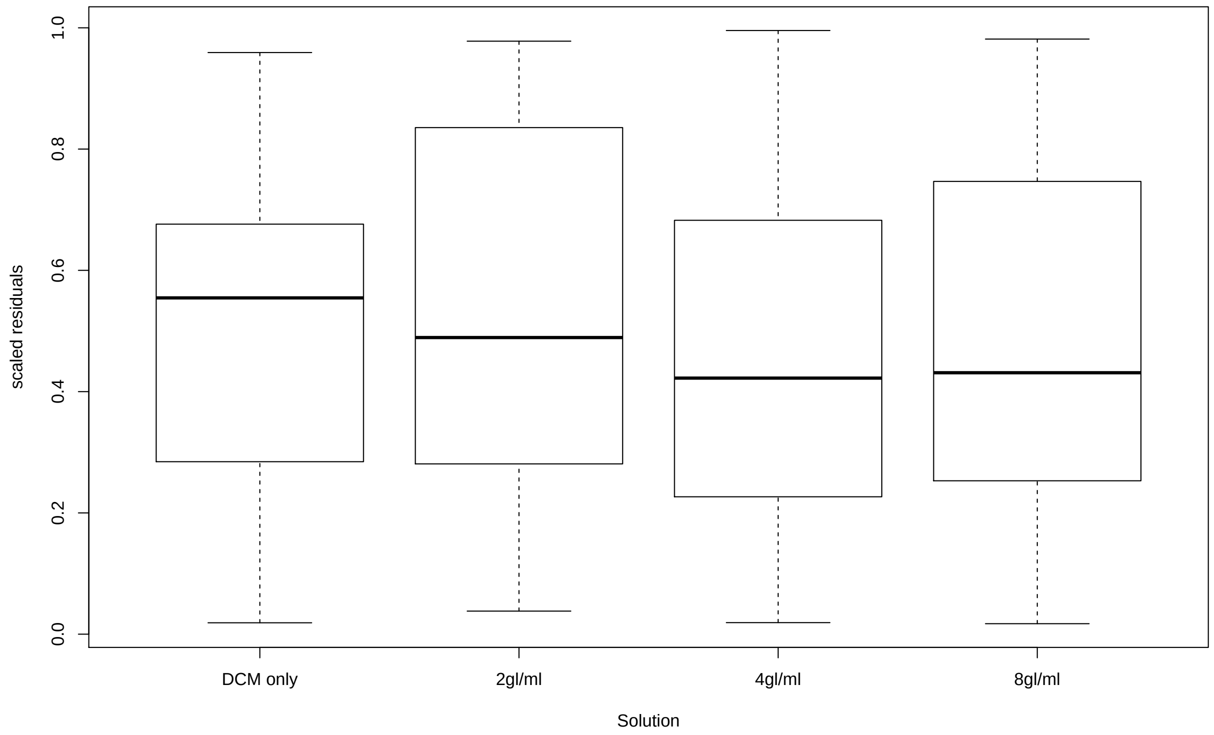

Results

```r
Anova(mtotalsetup)
```

```
## Analysis of Deviance Table (Type II Wald chisquare tests)
##
## Response: log(Total.time.on.setup)
##           Chisq Df Pr(>Chisq)
## Solution 5.2353  3     0.1554
```

```r
emmip(mtotalsetup, ~Solution,
      response=T,
      CIs = T,
      type="response")+
  ylab("Predicted total time on setup [sec]")+
  xlab("Solution")
```

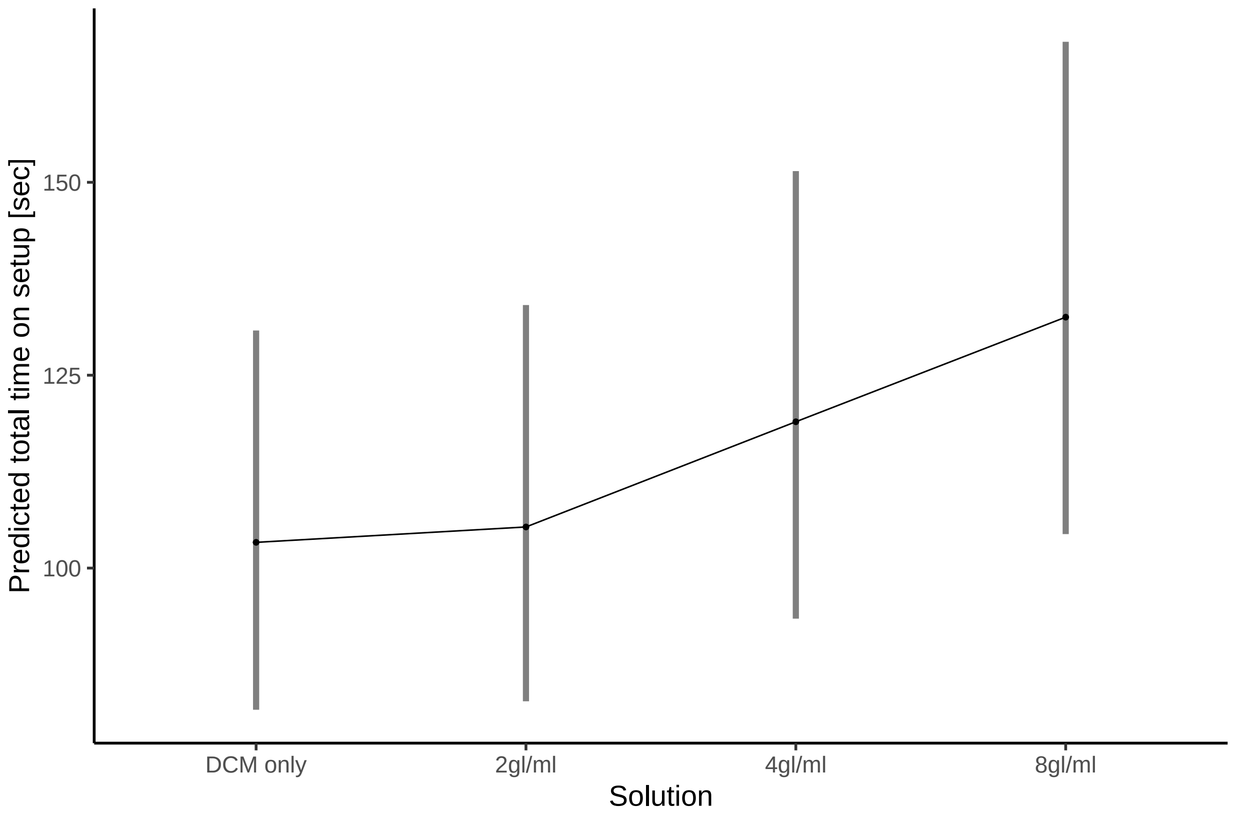

Panel figure

```
variables <-
  c("Duration.of.first.drinking", "Time.drinking", "Drinking.interruptions",
    "U.turns.to.nest", "Total.time.on.setup")
xlabs <- c("Duration first drinking [sec]", "Total time drinking [sec]",
           "Number of drinking interruptions", "U-turns to nest",
           "Total time on setup [sec]")

figs <- list()

for (plot in 1:length(variables)){
  figs[[plot]] <-
  ggplot(exp1, aes(x = Solution, y = .data[[variables[plot]]]))+
    geom_boxplot(outlier.size = 1) +
    scale_x_discrete(labels = c("DCM", "2gl/ml", "4gl/ml", "8gl/ml"))+
    ylab(paste0("\n", xlabs[plot]))+
    xlab(NULL)+
    theme_classic(8)
}

grid<-
plot_grid(nrow = 3, labels = "AUTO",
          figs[[1]]+coord_cartesian(ylim = c(0,210)),
          figs[[2]]+coord_cartesian(ylim = c(0,210)),
          figs[[3]]+coord_cartesian(ylim = c(0,20)),
          figs[[4]]+coord_cartesian(ylim = c(0,3)),
```

```
            figs[[5]])
grid
```

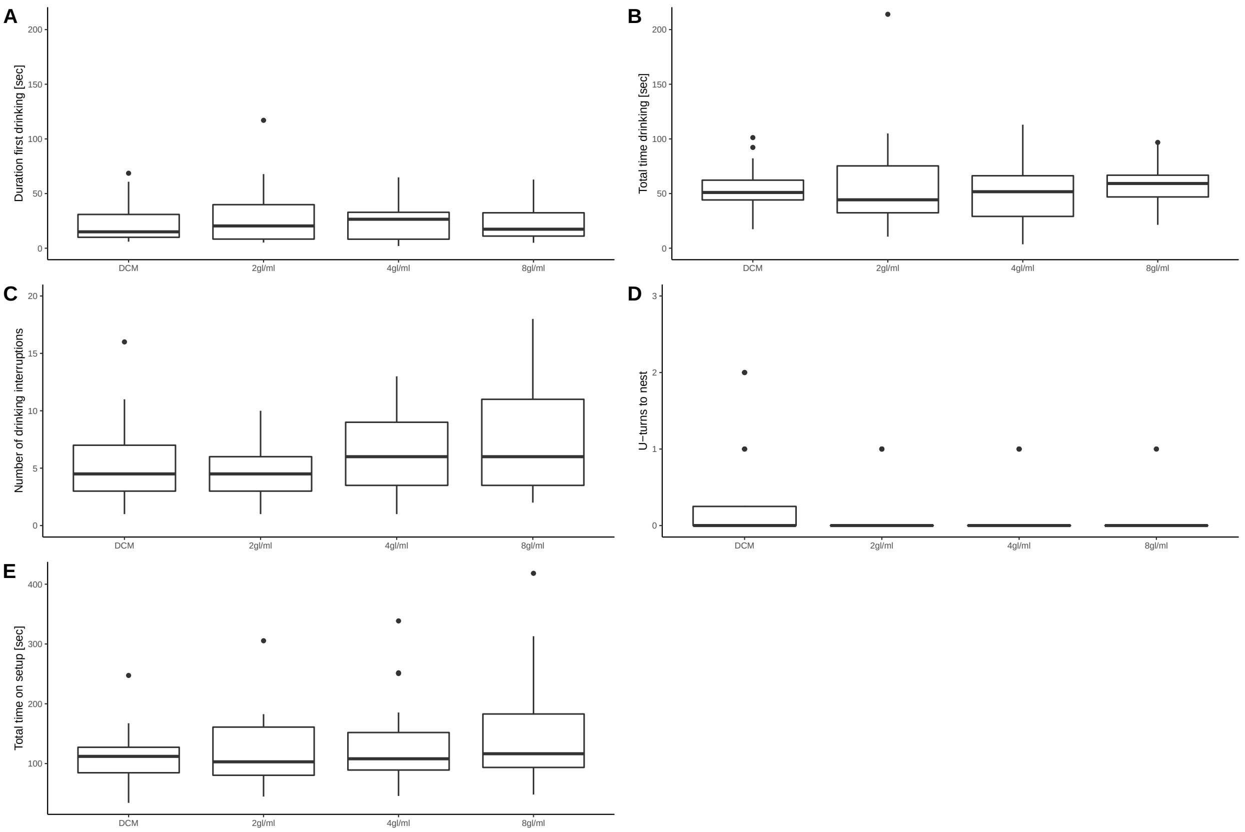

Experiment 2 - Food acceptance after two days of starvation

Load data

```
exp2 <- read.xlsx("ESM2_raw_data.xlsx", sheetIndex = 3)
exp2$Solution<-factor(
  exp2$Solution,
  levels = c("DCM", "2gl/ml", "4gl/ml", "8gl/ml"))
exp2 %>%
  filter(!is.na(Time.drinking)) %>%
{.->>exp2}
```

Sample size

```
exp2 %>%
  group_by(Solution) %>%
  tally() %>%
  adorn_totals("row") %>%
  create_table()
```

Solution	n
DCM	14
2gl/ml	16

Solution	n
8gl/ml	16
Total	46

Number of colonies tested:

```
length(levels(exp1$Colony))
```

```
## [1] 6
```

Analysis

Food acceptance

```
exp2 %>%
  mutate(Food.acceptance = ifelse(Duration.of.first.drinking >= 3,
                                  1,
                                  0)) %>%
  {.->>exp2} %>%
  group_by(Solution) %>%
  summarise(percent_accepting_food = mean(Food.acceptance)*100,
            n = length(Food.acceptance),
            not_accepting = n - sum(Food.acceptance)) %>%
  create_table(
    column_names = c("Solution", "% accepting food",
                     "Total ants", "Not accepting"),
    digits = 1)
```

Solution	% accepting food	Total ants	Not accepting
DCM	85.7	14	2
2gl/ml	100.0	16	0
8gl/ml	93.8	16	1

The majority of ants still accepts the food.

Duration of first drinking

One ant did not drink at all and is not used for the following analysis.

```
exp2 %>%
  filter(Duration.of.first.drinking > 0) %>%
  {.->>exp2}
```

Updated table:

```
exp2 %>%
  group_by(Solution) %>%
  summarise(percent_accepting_food = mean(Food.acceptance)*100,
            n = length(Food.acceptance),
            not_accepting = n - sum(Food.acceptance)) %>%
  create_table(
    column_names = c("Solution", "% accepting food",
                     "Total ants", "Not accepting"),
               digits = 1)
```

Solution	% accepting food	Total ants	Not accepting
DCM	92.3	13	1
2gl/ml	100.0	16	0
8gl/ml	93.8	16	1

```
m2first<- glmmTMB(log(Duration.of.first.drinking) ~ Solution + (1|Colony),
                  family = "gaussian",
                  data = exp2)
m2firstres <- simulateResiduals(m2first)
checkmodel(m2firstres)
```

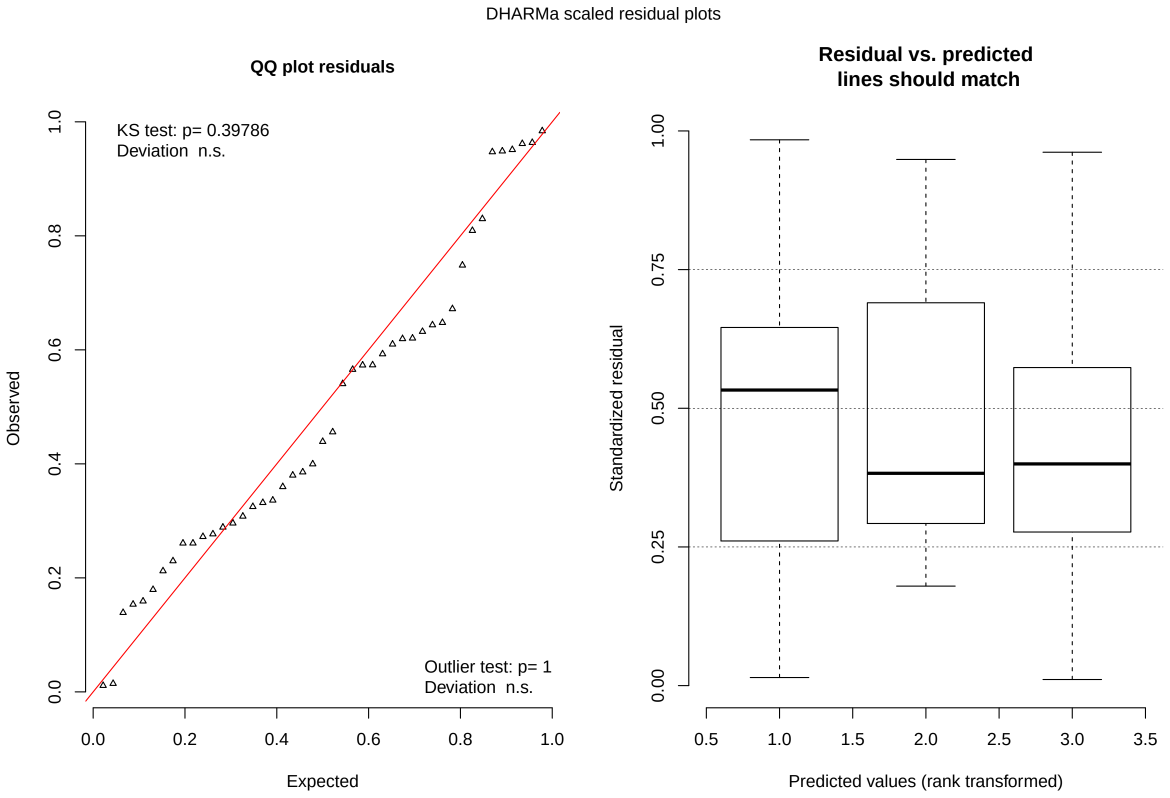

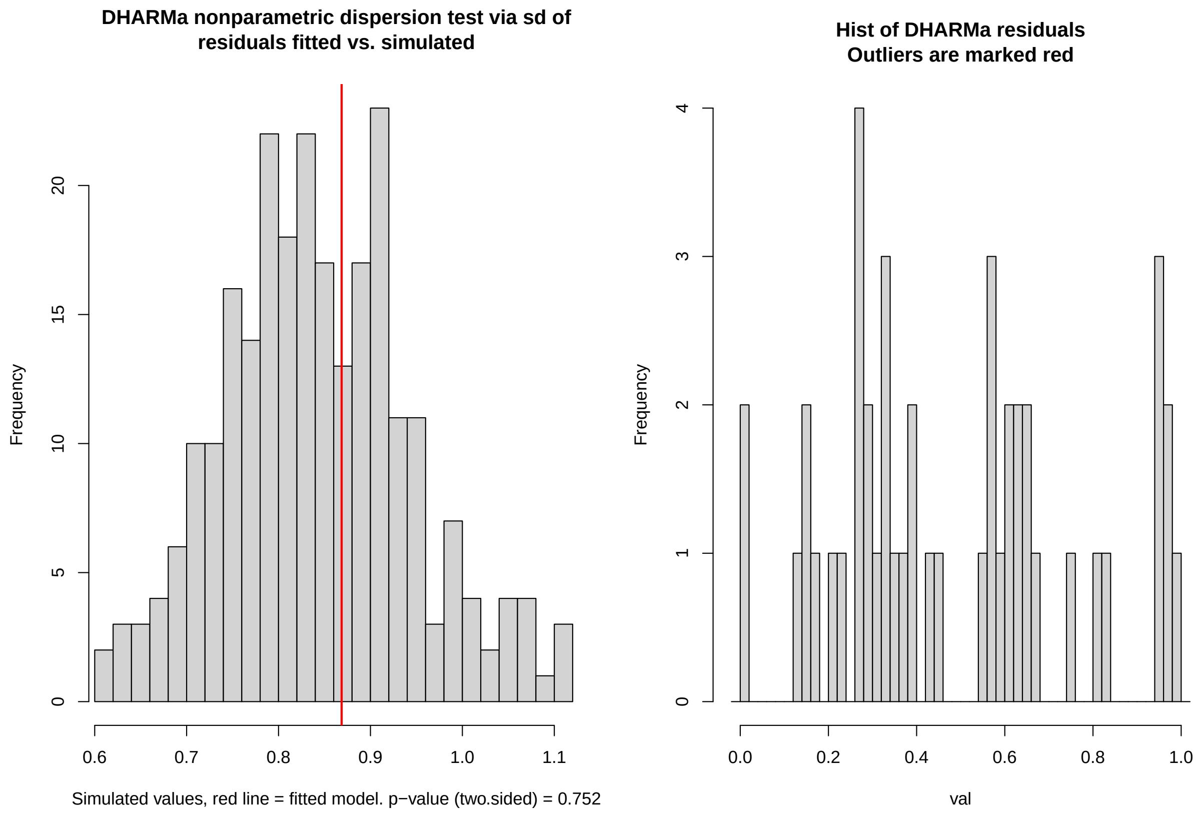


```
## $uniformity
##
##  One-sample Kolmogorov-Smirnov test
##
## data:  simulationOutput$scaledResiduals
## D = 0.13005, p-value = 0.3979
## alternative hypothesis: two-sided
##
##
## $dispersion
##
##  DHARMa nonparametric dispersion test via sd of residuals fitted vs.
##  simulated
##
## data:  simulationOutput
## ratioObsSim = 1.0307, p-value = 0.752
## alternative hypothesis: two.sided
##
##
## $outliers
##
##  DHARMa outlier test based on exact binomial test
##
## data:  simulationOutput
## outLow = 0.0000000, outHigh = 0.0000000, nobs = 45.0000000, freqH0 =
## 0.0039841, p-value = 1
## alternative hypothesis: two.sided
```

```r
plot_predictors(exp2, m2firstres, "Solution")
```

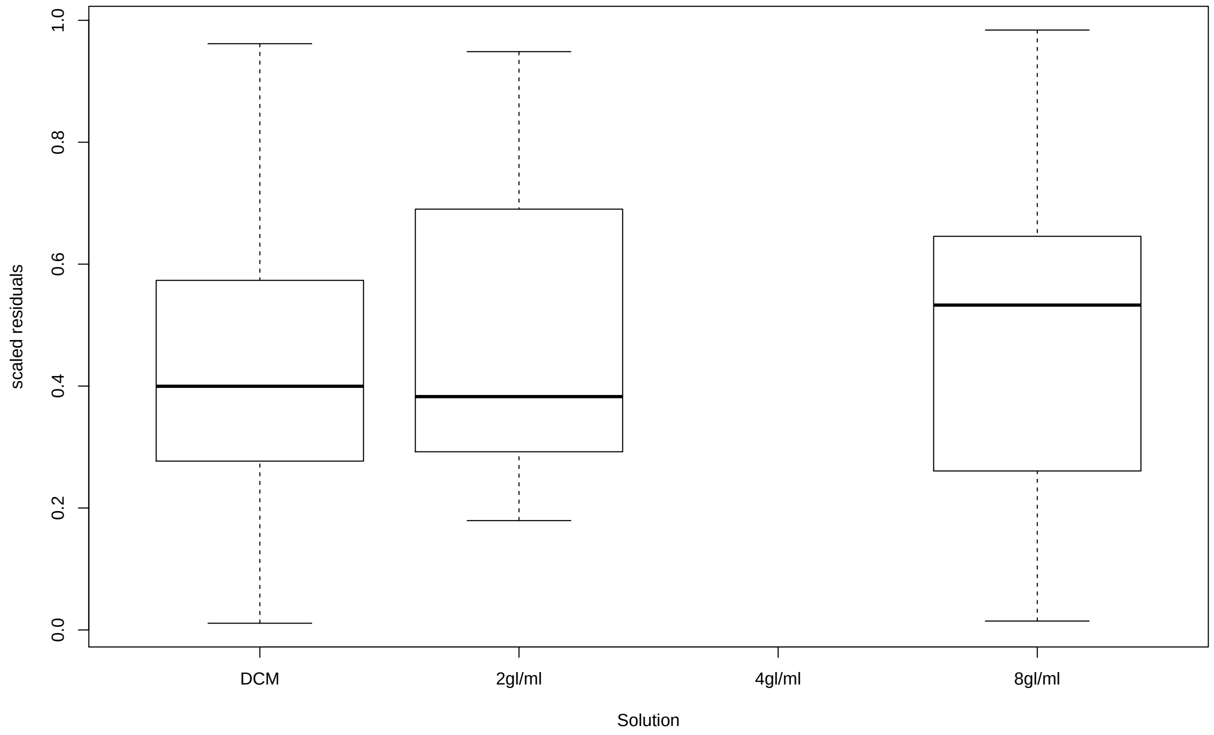

Results

```r
Anova(m2first)
```

```
## Analysis of Deviance Table (Type II Wald chisquare tests)
##
## Response: log(Duration.of.first.drinking)
##           Chisq Df Pr(>Chisq)
## Solution 1.2193  2     0.5435
```

```r
d<-
emmip(m2first, ~Solution,
      response=T,
      CIs = T,
      type="response")+
  ylab("Predicted duration of first drinking [sec]")+
  xlab("Solution")
d
```

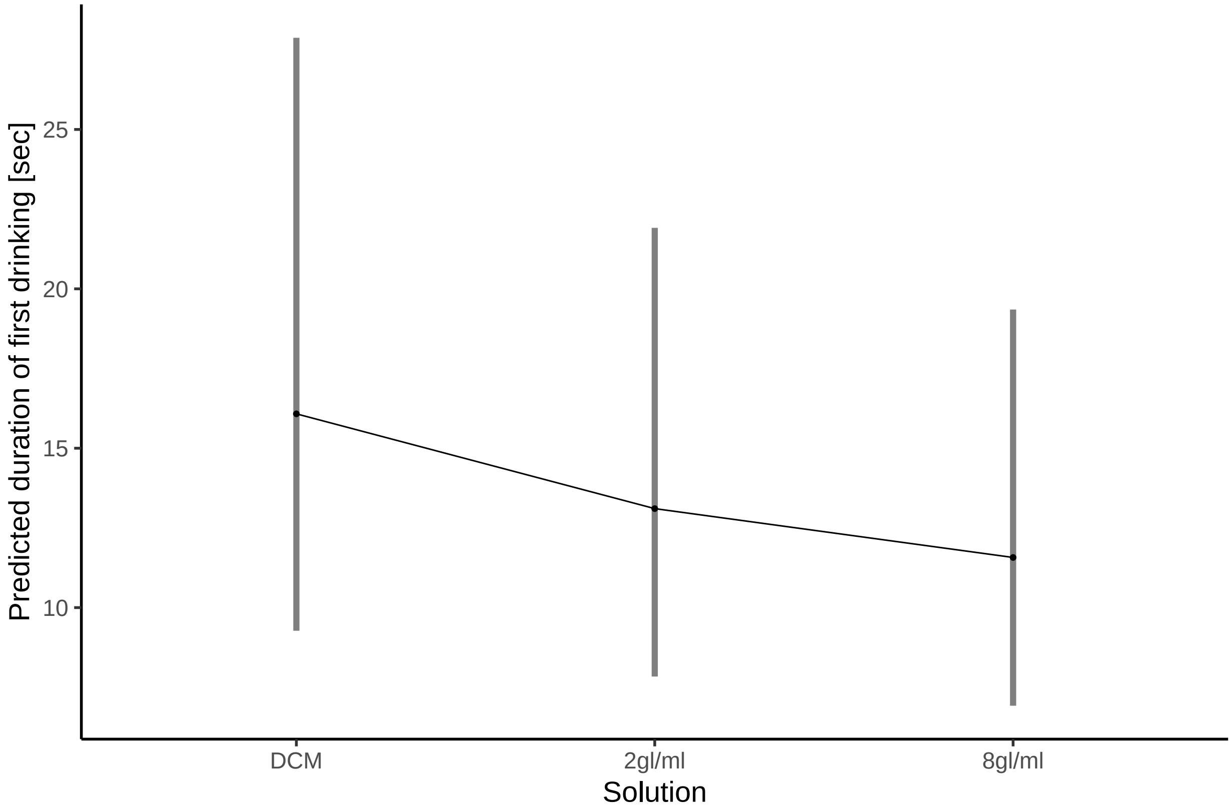

Total drinking time

```
m2total<- glmmTMB(log(Time.drinking) ~ Solution + (1|Colony),
                  family = "gaussian",
                  data = exp2)
m2totalres <- simulateResiduals(m2total)
plot(m2totalres)
```

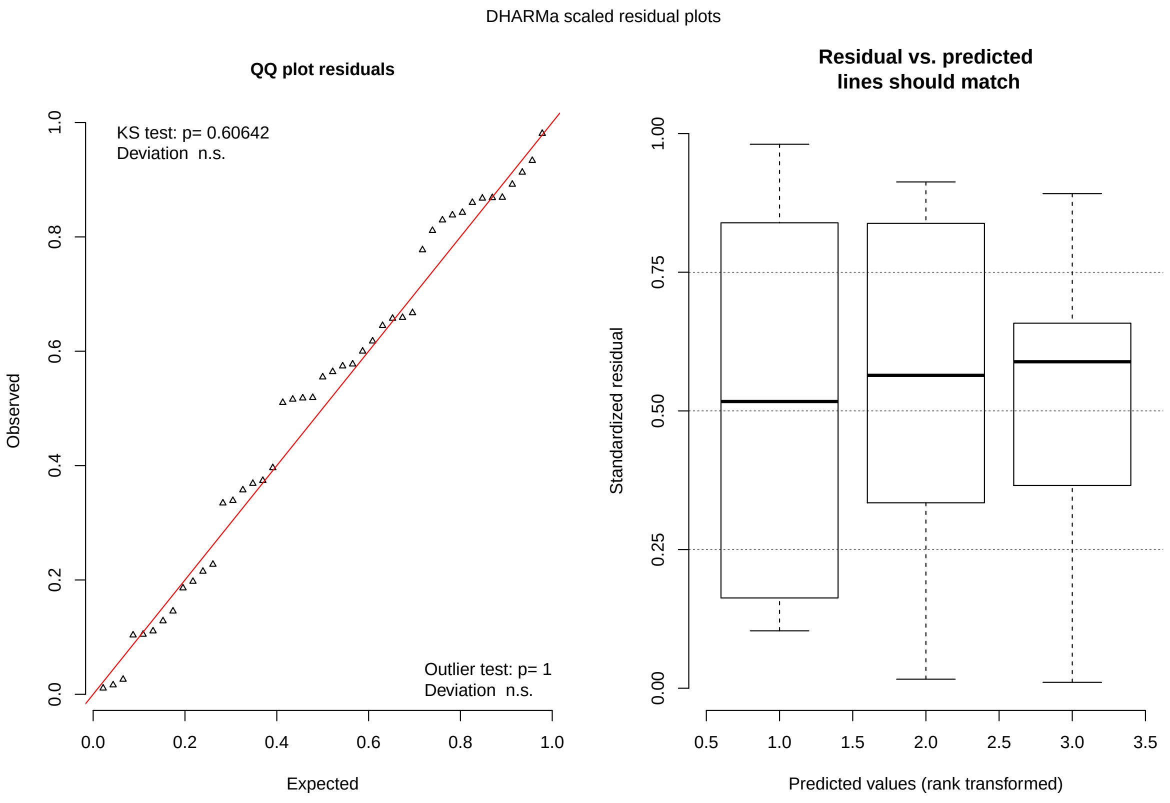


```
checkmodel(m2totalres)
```

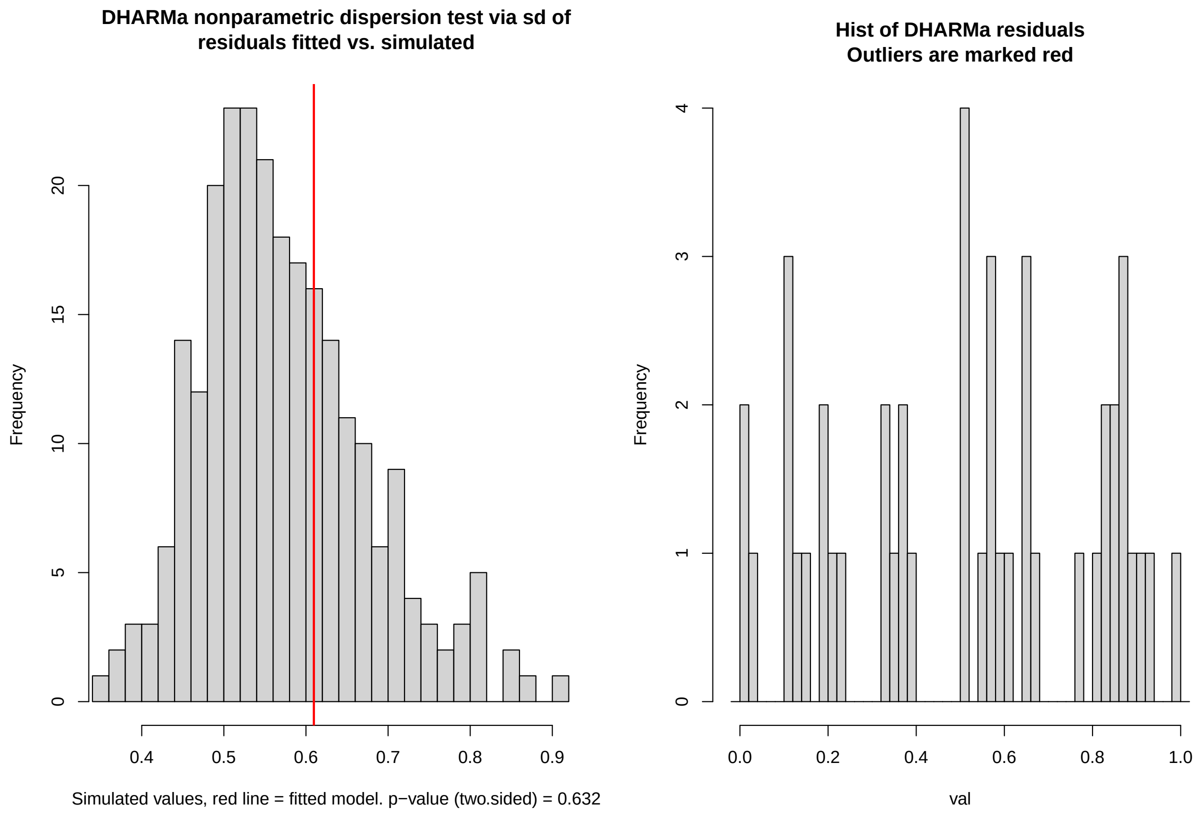


```
## $uniformity
##
##  One-sample Kolmogorov-Smirnov test
##
## data:  simulationOutput$scaledResiduals
## D = 0.11017, p-value = 0.6064
## alternative hypothesis: two-sided
##
##
## $dispersion
##
##  DHARMa nonparametric dispersion test via sd of residuals fitted vs.
##  simulated
##
## data:  simulationOutput
## ratioObsSim = 1.0651, p-value = 0.632
## alternative hypothesis: two.sided
##
##
## $outliers
##
##  DHARMa outlier test based on exact binomial test
##
## data:  simulationOutput
## outLow = 0.0000000, outHigh = 0.0000000, nobs = 45.0000000, freqH0 =
## 0.0039841, p-value = 1
## alternative hypothesis: two.sided
```

```r
plot_predictors(exp2, m2totalres, "Solution")
```

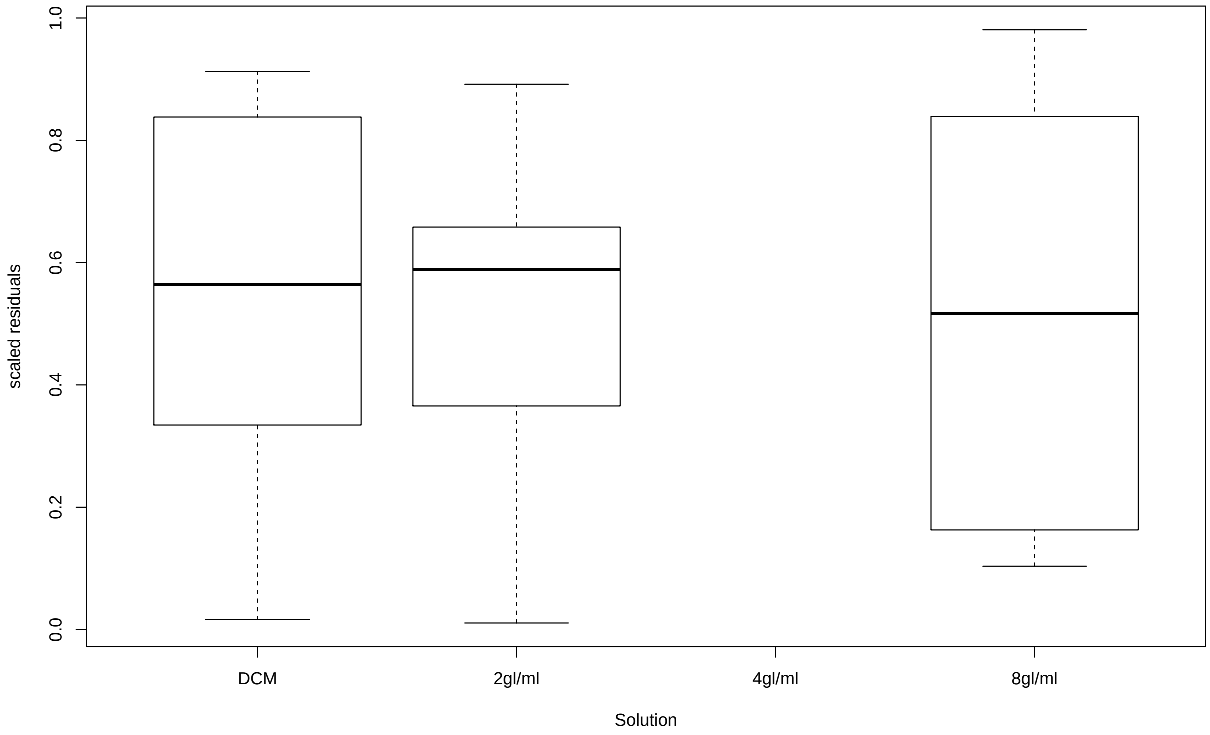

Results

```r
Anova(m2total)
```

```
## Analysis of Deviance Table (Type II Wald chisquare tests)
##
## Response: log(Time.drinking)
##           Chisq Df Pr(>Chisq)
## Solution 4.2232  2      0.121
```

```r
emmip(m2total, ~Solution, response=T, CIs = T, type="response")+
  ylab("Predicted total drinking time [sec]")+
  xlab("Solution")
```

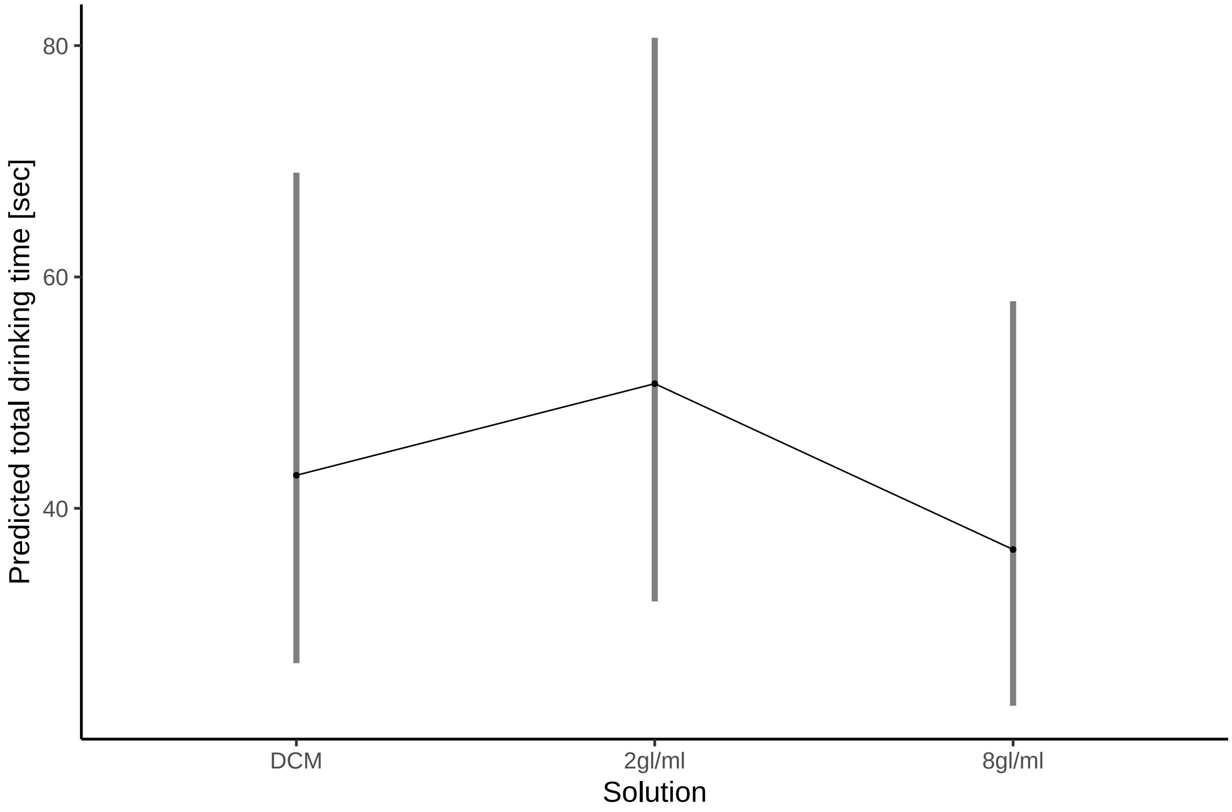

Interruptions

```
m2inter<-glmmTMB(Drinking.interruptions ~ Solution + (1|Colony),
                 data = exp2,
                 family = "poisson")
m2interres<-simulateResiduals(m2inter)
checkmodel(m2interres)
```

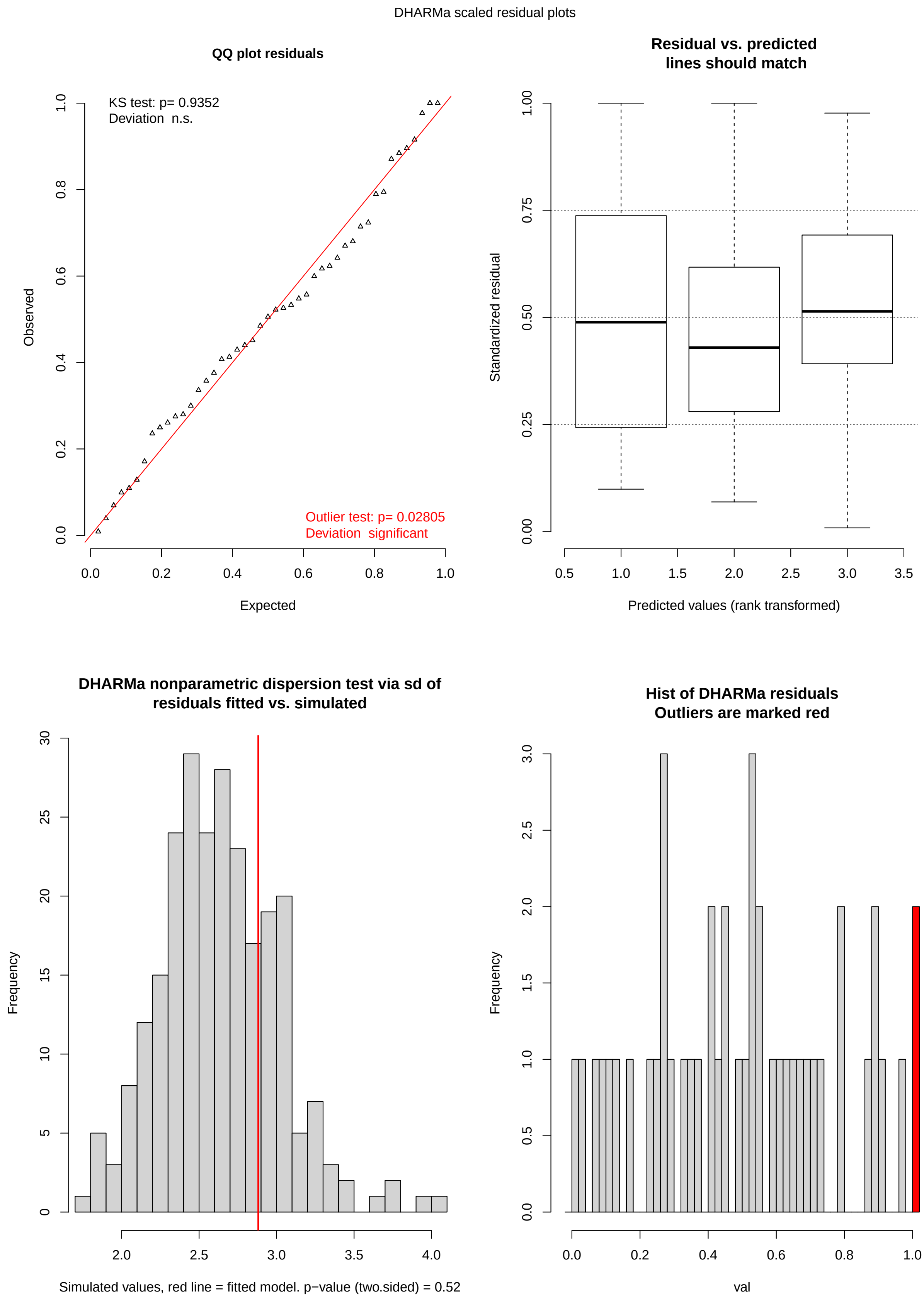
DHARMa scaled residual plots
QQ plot residuals
KS test: p= 0.9352
Deviation n.s.
Outlier test: p= 0.02805
Deviation significant
Observed
Expected
Residual vs. predicted
lines should match
Standardized residual
Predicted values (rank transformed)
DHARMa nonparametric dispersion test via sd of
residuals fitted vs. simulated
Frequency
Simulated values, red line = fitted model. p−value (two.sided) = 0.52
Hist of DHARMa residuals
Outliers are marked red
Frequency
val

```
## $uniformity
##
##  One-sample Kolmogorov-Smirnov test
##
## data:  simulationOutput$scaledResiduals
## D = 0.080063, p-value = 0.9352
## alternative hypothesis: two-sided
##
##
## $dispersion
##
##  DHARMa nonparametric dispersion test via sd of residuals fitted vs.
##  simulated
##
## data:  simulationOutput
## ratioObsSim = 1.0919, p-value = 0.52
## alternative hypothesis: two.sided
##
##
## $outliers
##
##  DHARMa outlier test based on exact binomial test
##
## data:  simulationOutput
## outLow = 0.0000000, outHigh = 2.0000000, nobs = 45.0000000, freqH0 =
## 0.0039841, p-value = 0.02805
## alternative hypothesis: two.sided
```

Model not fitting well. We will take negative binomial distribution instead.

```
m2inter<-glmmTMB(Drinking.interruptions ~ Solution + (1|Colony),
                 data = exp2,
                 family = "nbinom1")
m2interres<-simulateResiduals(m2inter)
checkmodel(m2interres)
```

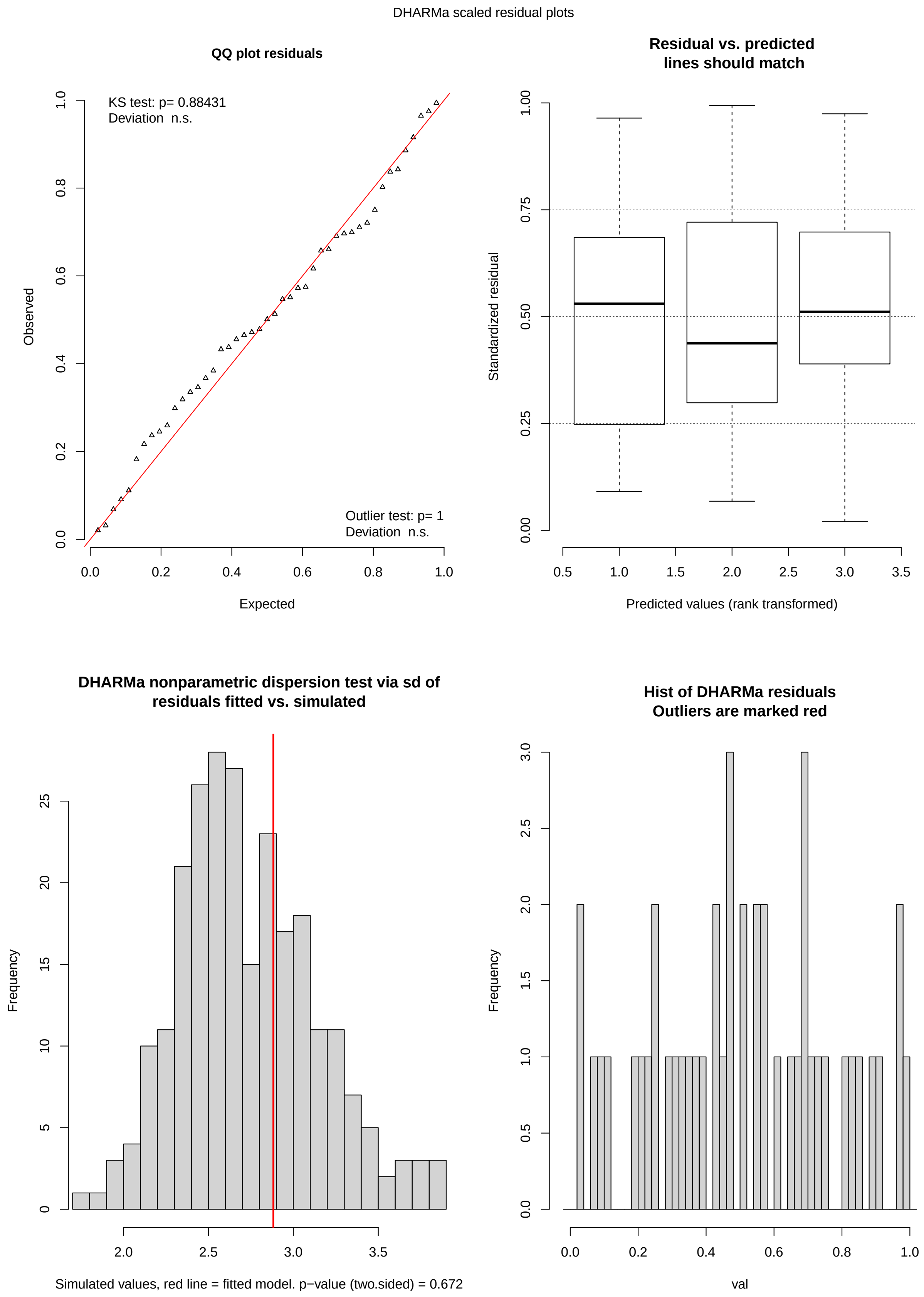
DHARMa scaled residual plots
QQ plot residuals
KS test: p= 0.88431
Deviation n.s.
Outlier test: p= 1
Deviation n.s.
Observed
Expected
Residual vs. predicted
lines should match
Standardized residual
Predicted values (rank transformed)
DHARMa nonparametric dispersion test via sd of
residuals fitted vs. simulated
Frequency
Simulated values, red line = fitted model. p−value (two.sided) = 0.672
Hist of DHARMa residuals
Outliers are marked red
Frequency
val

```
## $uniformity
##
##  One-sample Kolmogorov-Smirnov test
##
## data:  simulationOutput$scaledResiduals
## D = 0.083757, p-value = 0.8843
## alternative hypothesis: two-sided
##
##
## $dispersion
##
##  DHARMa nonparametric dispersion test via sd of residuals fitted vs.
##  simulated
##
## data:  simulationOutput
## ratioObsSim = 1.0541, p-value = 0.672
## alternative hypothesis: two.sided
##
##
## $outliers
##
##  DHARMa outlier test based on exact binomial test
##
## data:  simulationOutput
## outLow = 0.0000000, outHigh = 0.0000000, nobs = 45.0000000, freqH0 =
## 0.0039841, p-value = 1
## alternative hypothesis: two.sided
```

```
plot_predictors(exp2, m2interres, "Solution")
```

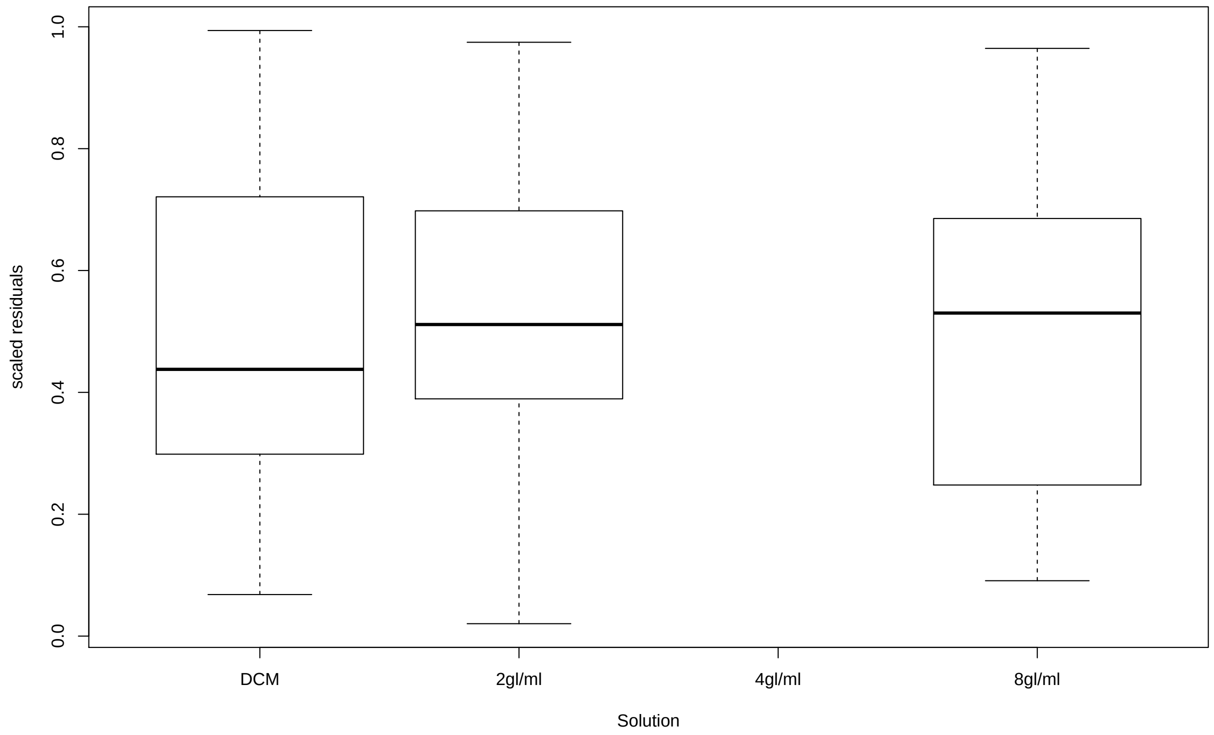

Better.

Results

```
Anova(m2inter)
```

```
## Analysis of Deviance Table (Type II Wald chisquare tests)
##
## Response: Drinking.interruptions
##           Chisq Df Pr(>Chisq)
## Solution 1.7908  2     0.4084
```

```
emmip(m2inter, ~Solution,
      response=T,
      CIs = T,
      type="response")+
  ylab("Predicted number of drinking interruptions")+
  xlab("Solution")
```

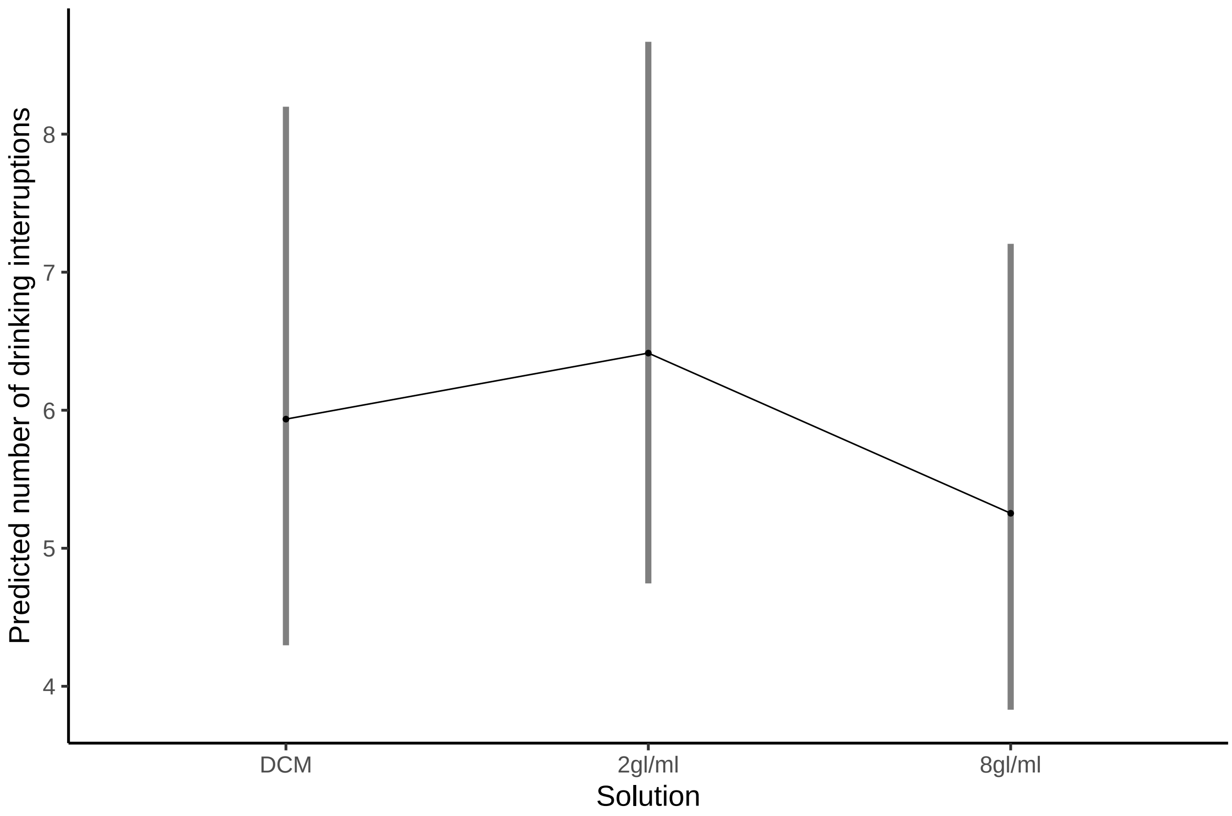

U-turns to food

```
exp2 %>%
  mutate(turned = ifelse(U.turns.to.food > 0, "yes", "no")) %>%
  group_by(turned, Solution) %>%
  tally %>%
  create_table()
```

turned	Solution	n
no	DCM	6
no	2gl/ml	7
no	8gl/ml	3
yes	DCM	7
yes	2gl/ml	9
yes	8gl/ml	13

```
m2turns<- glmmTMB(U.turns.to.food ~ Solution + (1|Colony),
                  family = "poisson",
                  data = exp2)
m2turnsres <- simulateResiduals(m2turns)
checkmodel(m2turnsres)
```

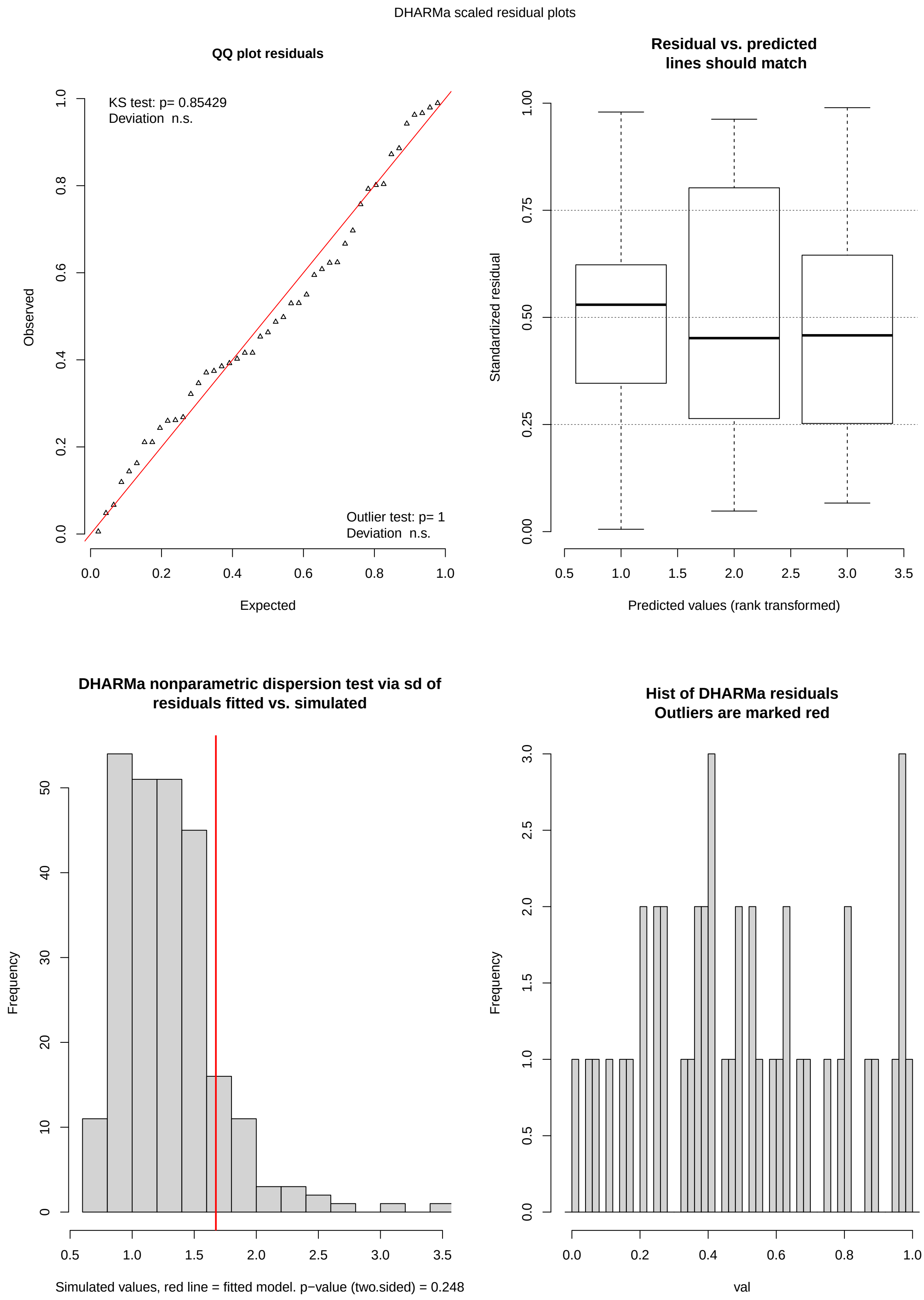
DHARMa scaled residual plots
QQ plot residuals
KS test: p= 0.85429
Deviation n.s.
Outlier test: p= 1
Deviation n.s.
Observed
Expected
Residual vs. predicted
lines should match
Standardized residual
Predicted values (rank transformed)
DHARMa nonparametric dispersion test via sd of
residuals fitted vs. simulated
Frequency
Simulated values, red line = fitted model. p−value (two.sided) = 0.248
Hist of DHARMa residuals
Outliers are marked red
Frequency
val

```
## $uniformity
##
##  One-sample Kolmogorov-Smirnov test
##
## data:  simulationOutput$scaledResiduals
## D = 0.087182, p-value = 0.8543
## alternative hypothesis: two-sided
##
##
## $dispersion
##
##  DHARMa nonparametric dispersion test via sd of residuals fitted vs.
##  simulated
##
## data:  simulationOutput
## ratioObsSim = 1.3049, p-value = 0.248
## alternative hypothesis: two.sided
##
##
## $outliers
##
##  DHARMa outlier test based on exact binomial test
##
## data:  simulationOutput
## outLow = 0.0000000, outHigh = 0.0000000, nobs = 45.0000000, freqH0 =
## 0.0039841, p-value = 1
## alternative hypothesis: two.sided
```

```
plot_predictors(exp2, m2turnsres, "Solution")
```

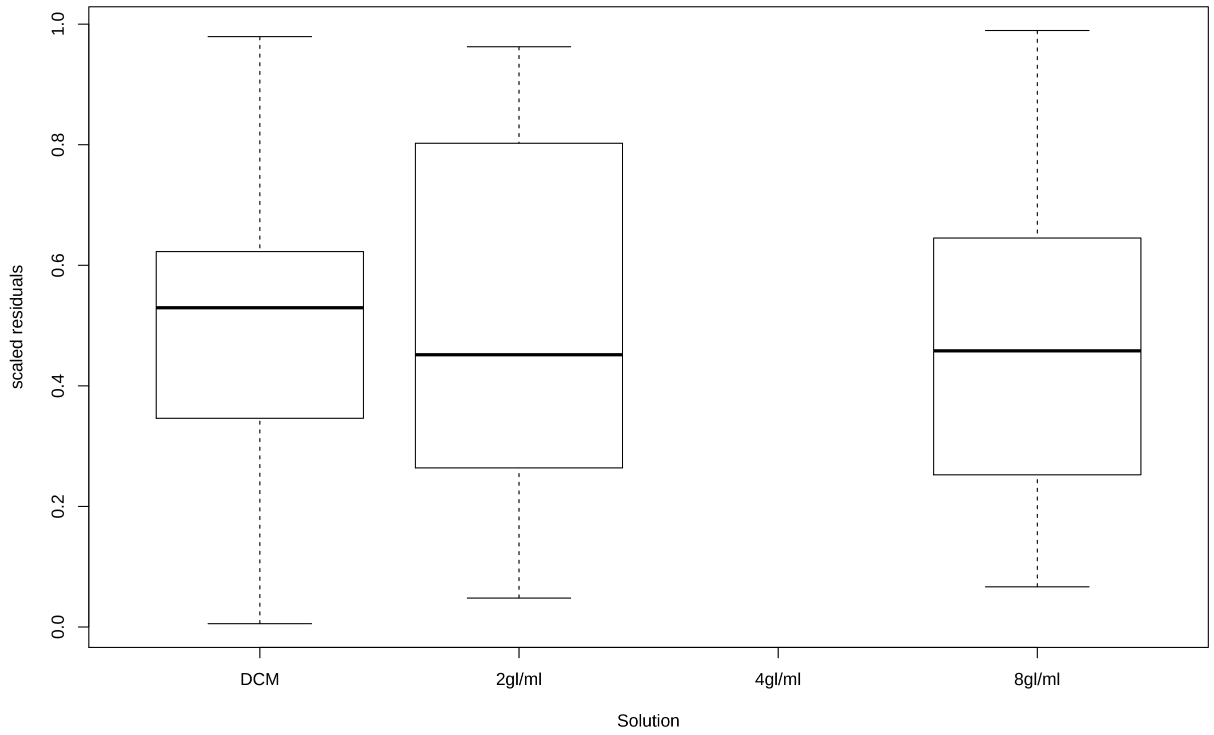

Results

```
Anova(m2turns)
```

```
## Analysis of Deviance Table (Type II Wald chisquare tests)
##
## Response: U.turns.to.food
##           Chisq Df Pr(>Chisq)
## Solution 6.6934  2     0.0352 *
## ---
## Signif. codes:  0 '***' 0.001 '**' 0.01 '*' 0.05 '.' 0.1 ' ' 1
```

```
e<-
emmip(m2turns, ~Solution, response=T, CIs = T, type="response")+
  ylab("Predicted number of u-turns to food")+
  xlab("Solution")
e
```

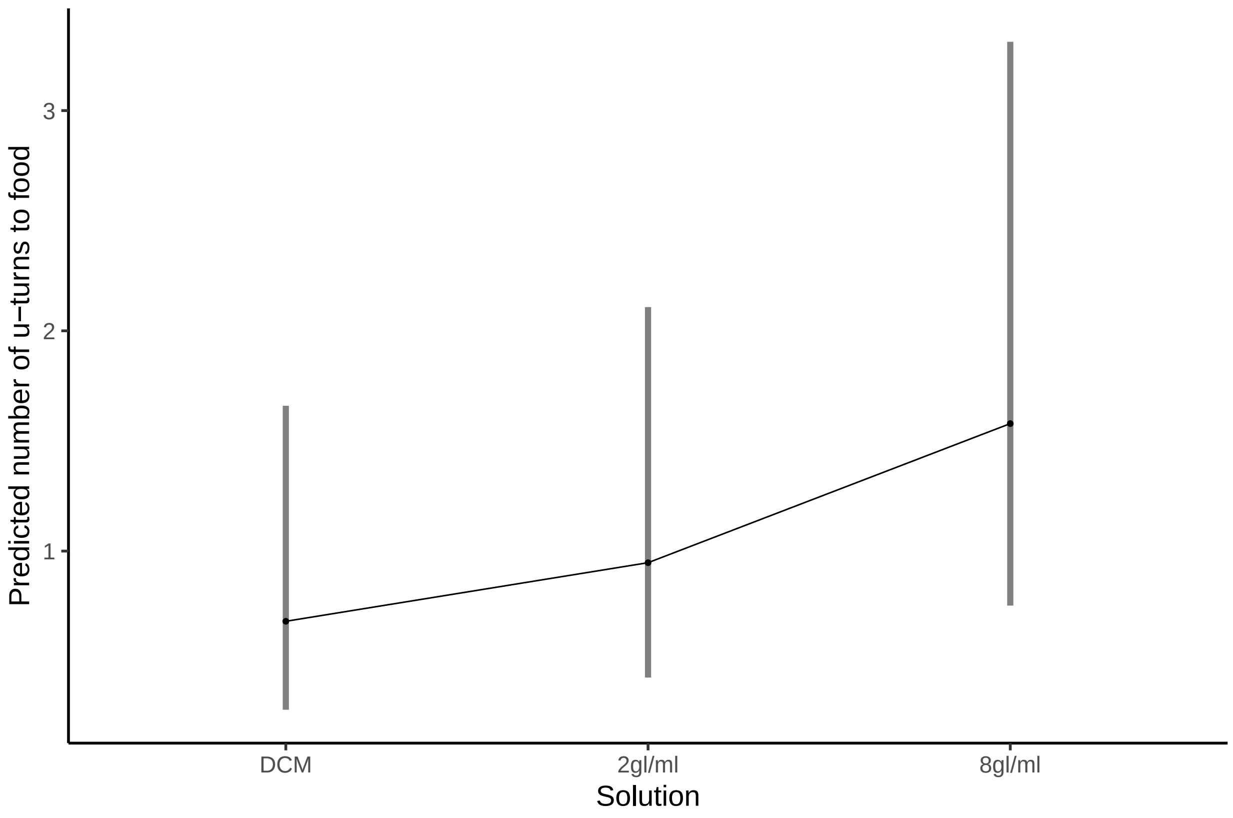


```
emm <- emmeans(m2turns, ~Solution)
merge(
contrast(emm, method = "trt.vs.ctrl", type="response", adjust="mvt"),
confint(contrast(emm, method = "trt.vs.ctrl", type="response", adjust="mvt"))[c(1,5,6)],
by = "contrast")
```

```
##       contrast    ratio        SE df   t.ratio    p.value  lower.CL upper.CL
## 1 2gl/ml / DCM 1.390597 0.5328155 41 0.8605728 0.56851111 0.5847493 3.306992
## 2 8gl/ml / DCM 2.317662 0.8180597 41 2.3814050 0.03805866 1.0434244 5.148008
```

U-turns to nest

```
exp2 %>%
  mutate(turned = ifelse(U.turns.to.nest > 0, "yes", "no")) %>%
  group_by(turned, Solution) %>%
  tally %>%
  create_table()
```

turned	Solution	n
no	DCM	9
no	2gl/ml	15
no	8gl/ml	13
yes	DCM	4
yes	2gl/ml	1
yes	8gl/ml	3

```
m2turnsnest<- glmmTMB(U.turns.to.nest ~ Solution + (1|Colony),
```

```
                   family = "poisson",
                   data = exp2)
m2turnsnestres <- simulateResiduals(m2turnsnest)
checkmodel(m2turnsnestres)
```

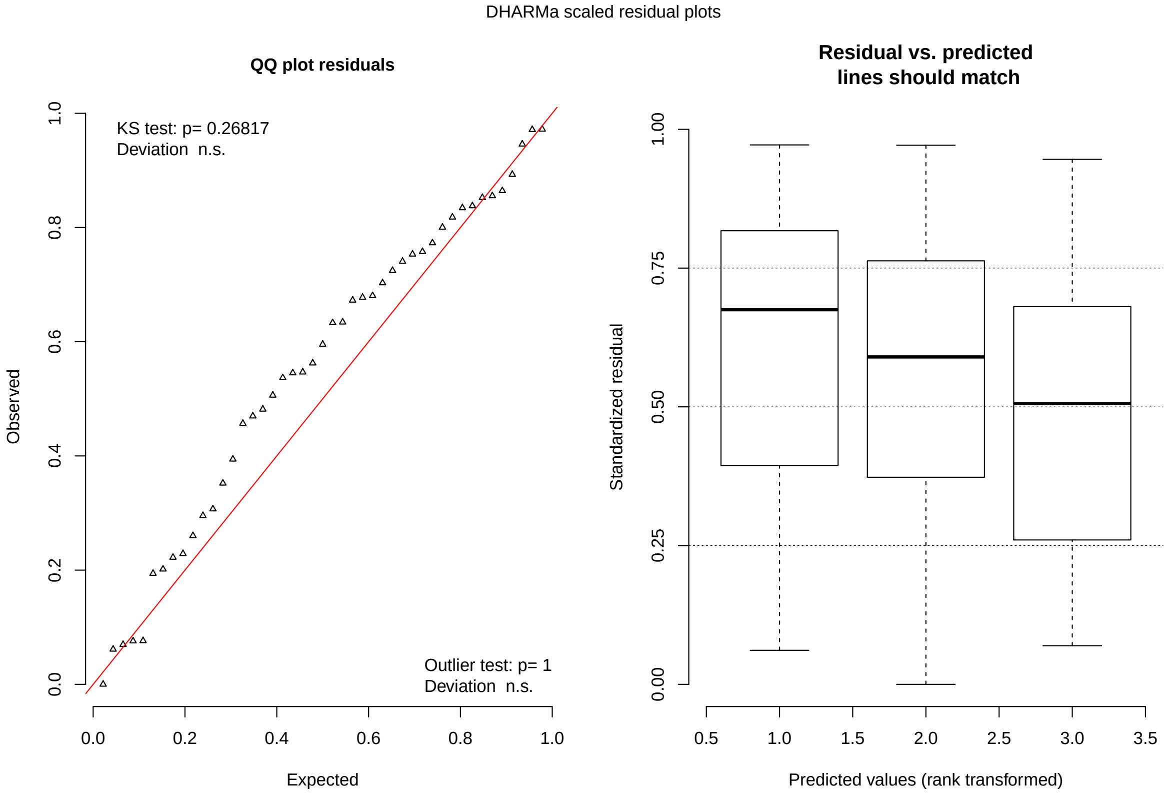

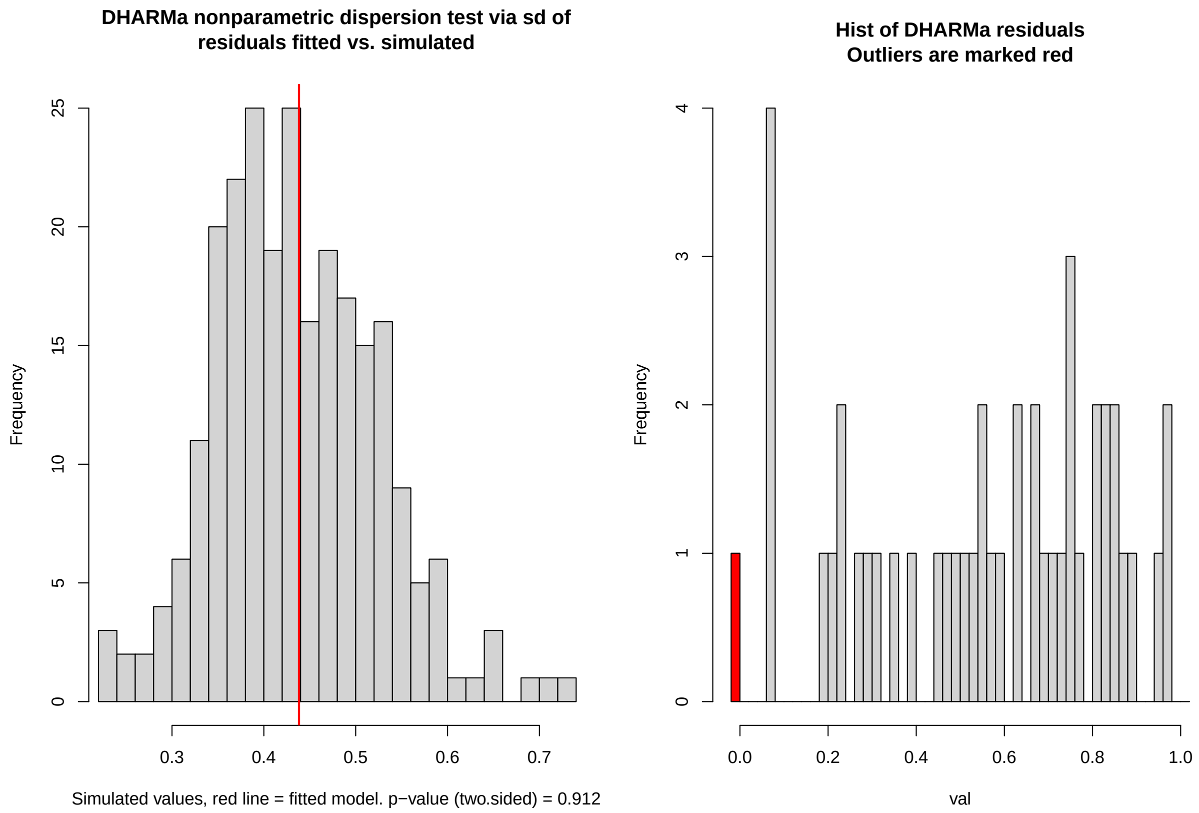


```
## $uniformity
##
##  One-sample Kolmogorov-Smirnov test
##
## data:  simulationOutput$scaledResiduals
## D = 0.14568, p-value = 0.2682
## alternative hypothesis: two-sided
##
##
## $dispersion
##
##  DHARMa nonparametric dispersion test via sd of residuals fitted vs.
##  simulated
##
## data:  simulationOutput
## ratioObsSim = 1.004, p-value = 0.912
## alternative hypothesis: two.sided
##
##
## $outliers
##
##  DHARMa outlier test based on exact binomial test
##
## data:  simulationOutput
## outLow = 1.0000000, outHigh = 0.0000000, nobs = 45.0000000, freqH0 =
## 0.0039841, p-value = 1
## alternative hypothesis: two.sided
```

```r
plot_predictors(exp2, m2turnsnestres, "Solution")
```

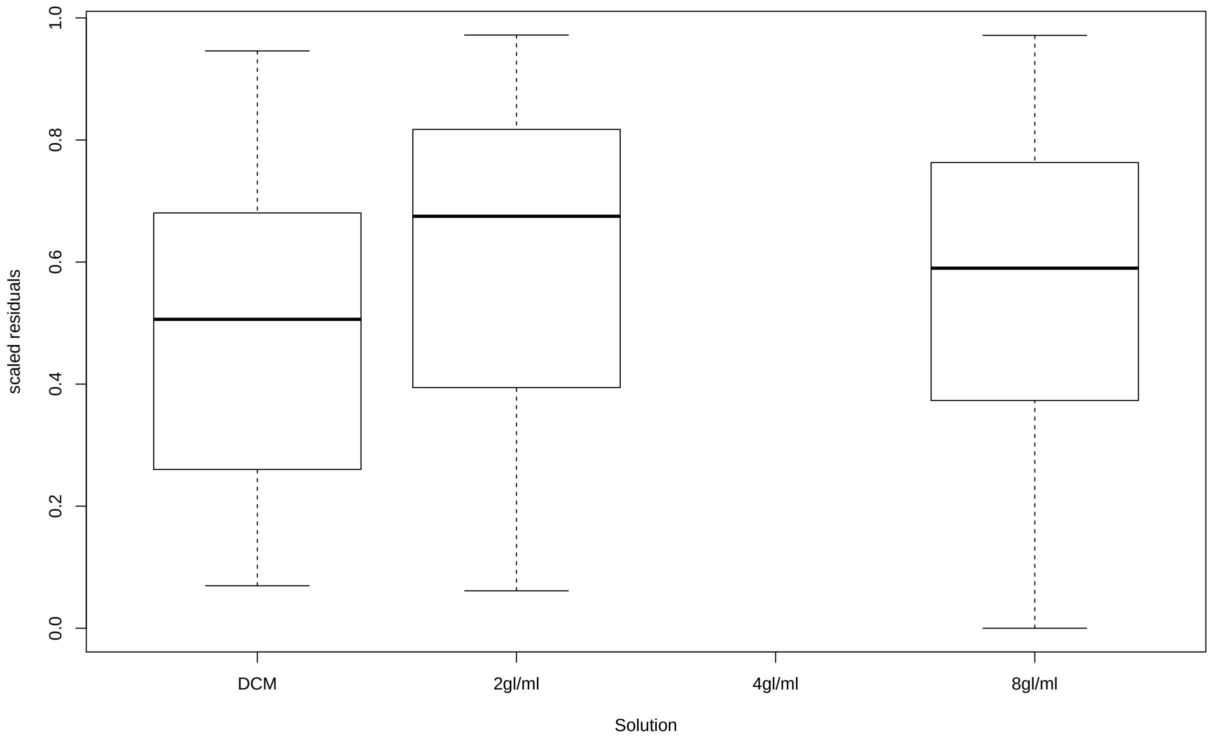

Results

```r
Anova(m2turnsnest)
```

```
## Analysis of Deviance Table (Type II Wald chisquare tests)
##
## Response: U.turns.to.nest
##          Chisq Df Pr(>Chisq)
## Solution 3.097  2     0.2126
```

```r
emmip(m2turnsnest, ~Solution, response=T, CIs = T, type="response")+
  ylab("Predicted number of u-turns to nest")+
  xlab("Solution")
```

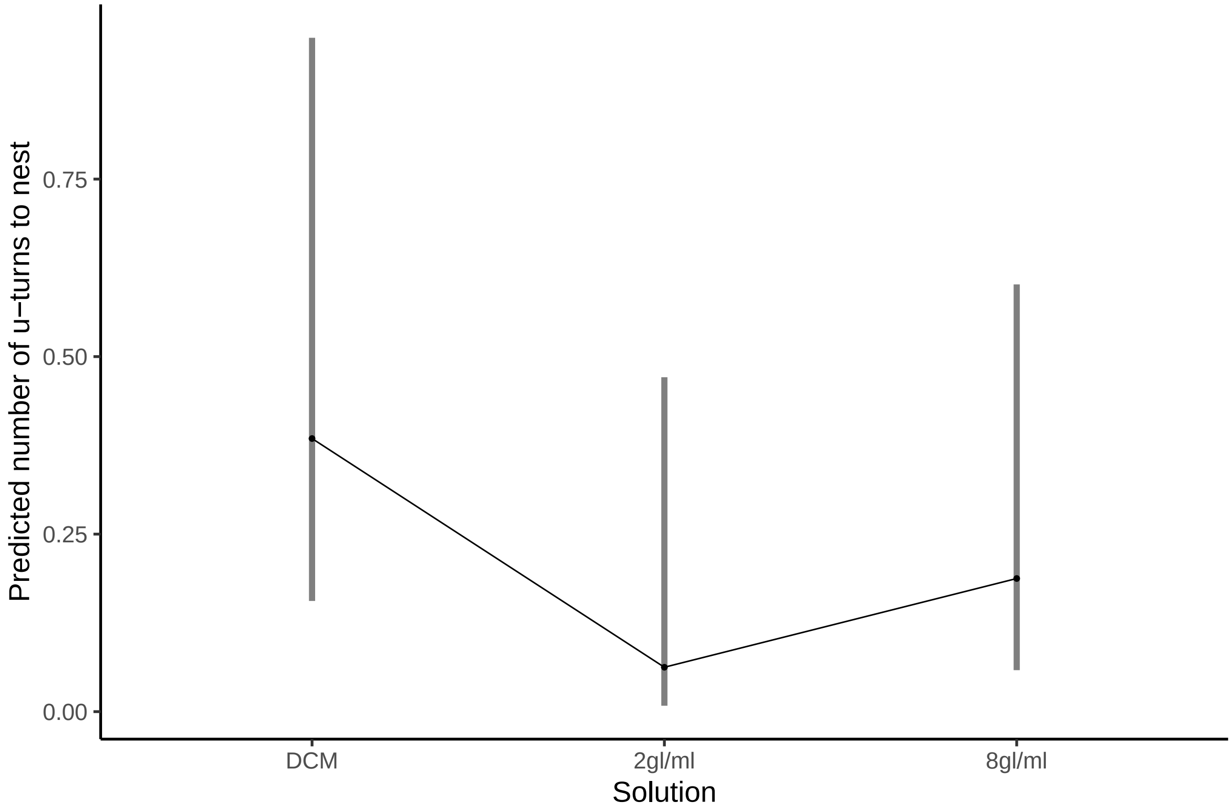

Duration to food

```
m2tofood<-glmmTMB(log(Time.spent.to.food) ~ Solution + (1|Colony),
                  data = exp2,
                  family = "gaussian")

m2tofoodres<-simulateResiduals(m2tofood)
checkmodel(m2tofoodres)
```

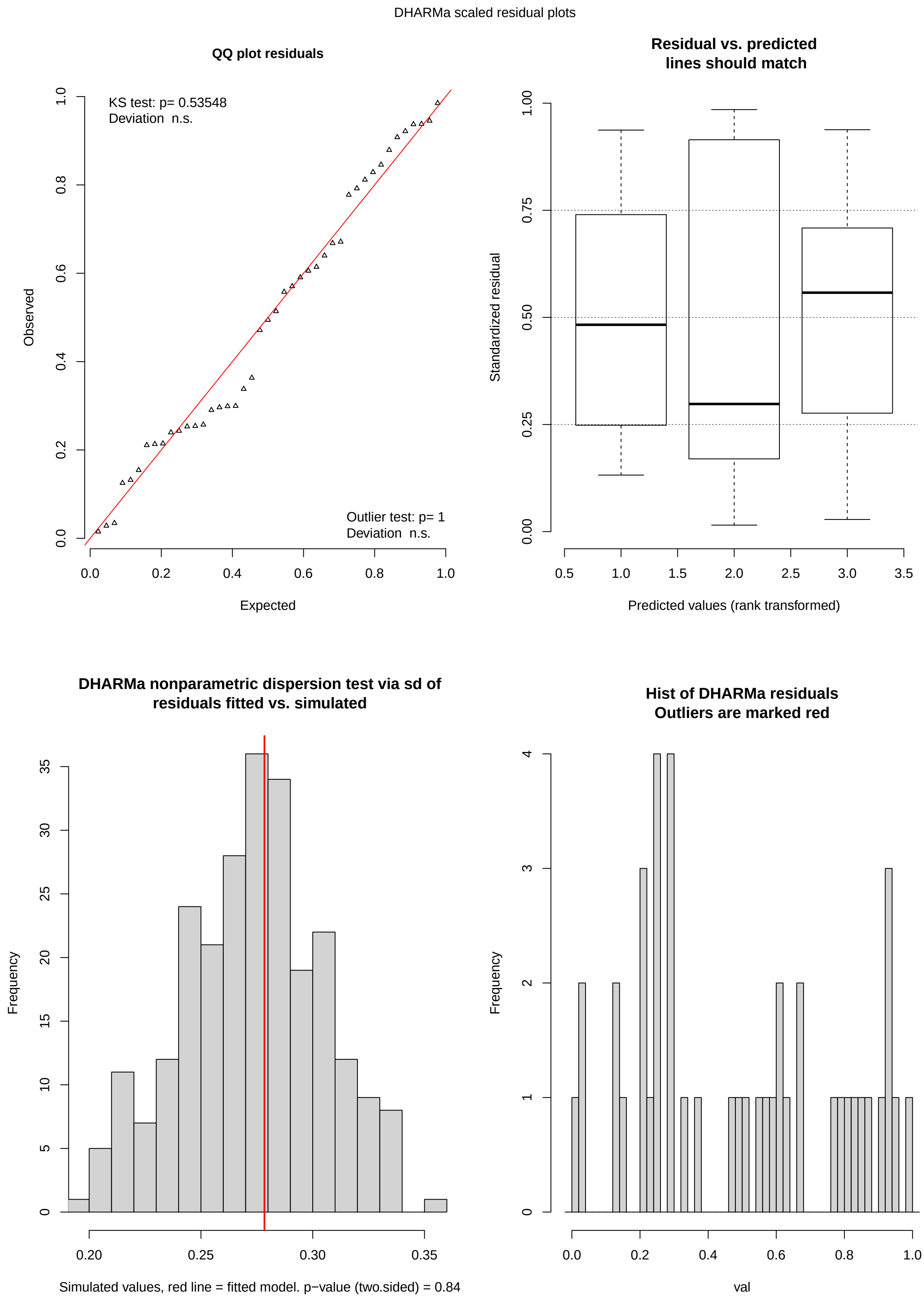

DHARMa scaled residual plots
QQ plot residuals
KS test: p= 0.53548
Deviation n.s.
Outlier test: p= 1
Deviation n.s.
Observed
Expected
Residual vs. predicted
lines should match
Standardized residual
Predicted values (rank transformed)
DHARMa nonparametric dispersion test via sd of
residuals fitted vs. simulated
Frequency
Simulated values, red line = fitted model. p−value (two.sided) = 0.84
Hist of DHARMa residuals
Outliers are marked red
Frequency
val

```
## $uniformity
##
##  One-sample Kolmogorov-Smirnov test
##
## data:  simulationOutput$scaledResiduals
## D = 0.11915, p-value = 0.5355
## alternative hypothesis: two-sided
##
##
## $dispersion
##
##  DHARMa nonparametric dispersion test via sd of residuals fitted vs.
##  simulated
##
## data:  simulationOutput
## ratioObsSim = 1.0191, p-value = 0.84
## alternative hypothesis: two.sided
##
##
## $outliers
##
##  DHARMa outlier test based on exact binomial test
##
## data:  simulationOutput
## outLow = 0.0000000, outHigh = 0.0000000, nobs = 43.0000000, freqH0 =
## 0.0039841, p-value = 1
## alternative hypothesis: two.sided
```

```
plot_predictors(na.omit(exp2), m2tofoodres, "Solution") #2 ants have NAs
```

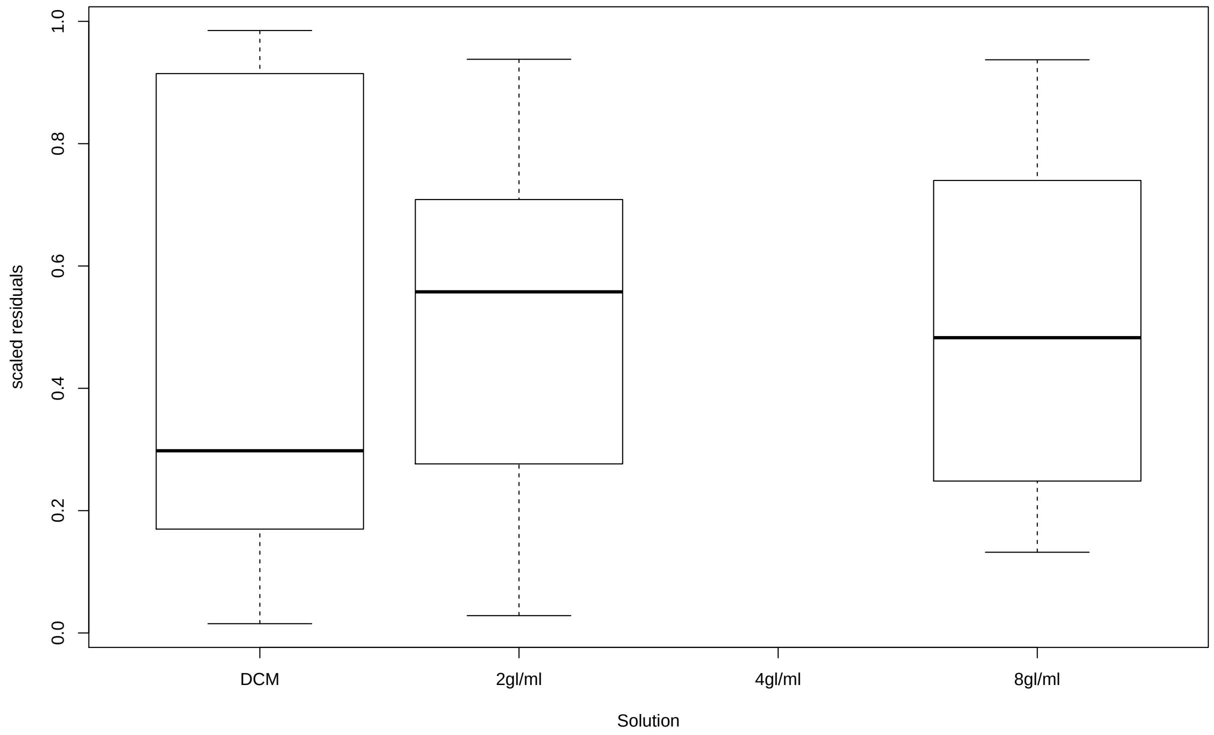

Results

```
Anova(m2tofood)
```

```
## Analysis of Deviance Table (Type II Wald chisquare tests)
##
## Response: log(Time.spent.to.food)
##           Chisq Df Pr(>Chisq)
## Solution 2.5558  2     0.2786
```

```
emmip(m2tofood, ~Solution,
      response=T,
      CIs = T,
      type="response")+
  ylab("Predicted time to food [sec]")+
  xlab("Solution")
```

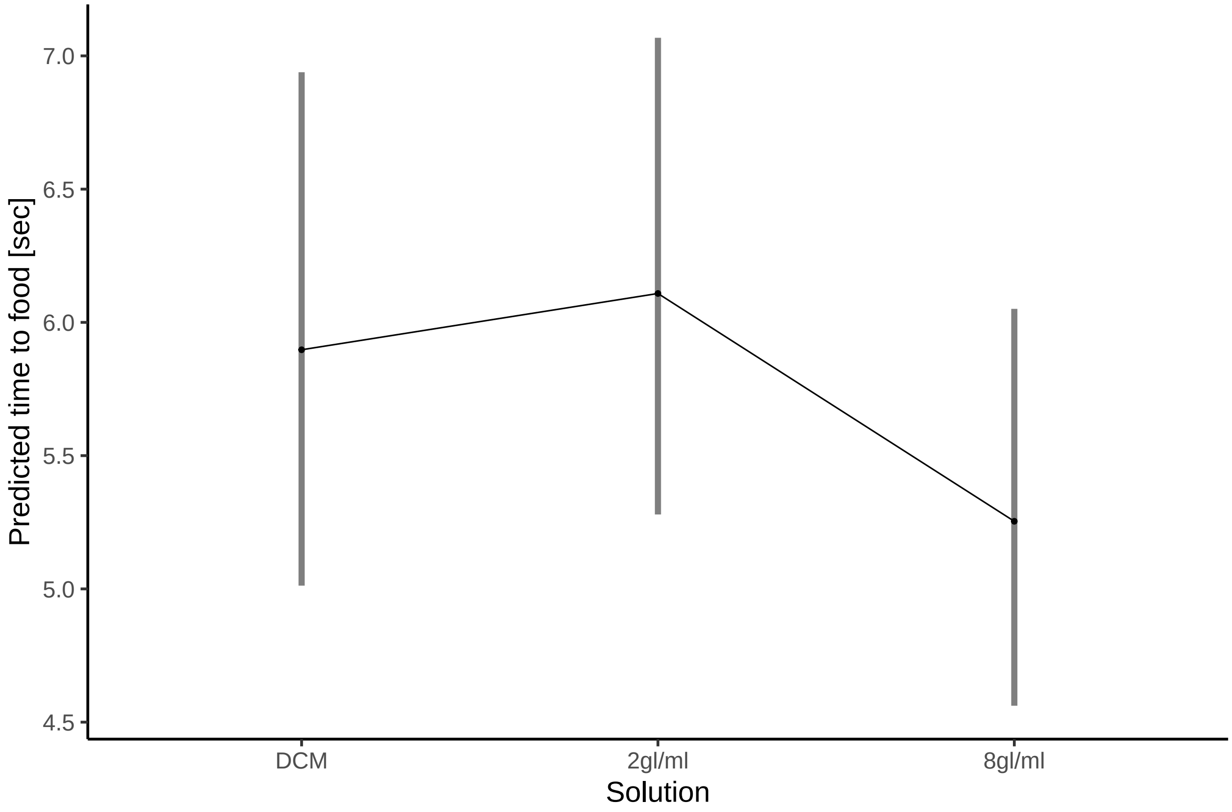


```
emmeans(m2tofood, trt.vs.ctrl  ~ Solution,
        type="response",
        adjust="mvt")
```

```
## $emmeans
##  Solution response    SE df lower.CL upper.CL
##  DCM          5.90 0.474 38     4.83     7.21
##  2gl/ml       6.11 0.440 38     5.10     7.31
##  8gl/ml       5.25 0.367 38     4.41     6.25
##
## Confidence level used: 0.95
## Conf-level adjustment: mvt method for 3 estimates
## Intervals are back-transformed from the log scale
##
## $contrasts
##  contrast     ratio     SE df t.ratio p.value
##  2gl/ml / DCM 1.036 0.1101 38   0.331  0.9201
##  8gl/ml / DCM 0.891 0.0932 38  -1.104  0.4344
##
## P value adjustment: mvt method for 2 tests
## Tests are performed on the log scale
```

Total time on setup

```
m2totalsetup<- glmmTMB(log(Total.time.on.setup) ~ Solution + (1|Colony),
                       family = "gaussian", data = exp2)
m2totalres <- simulateResiduals(m2totalsetup)
checkmodel(m2totalres)
```

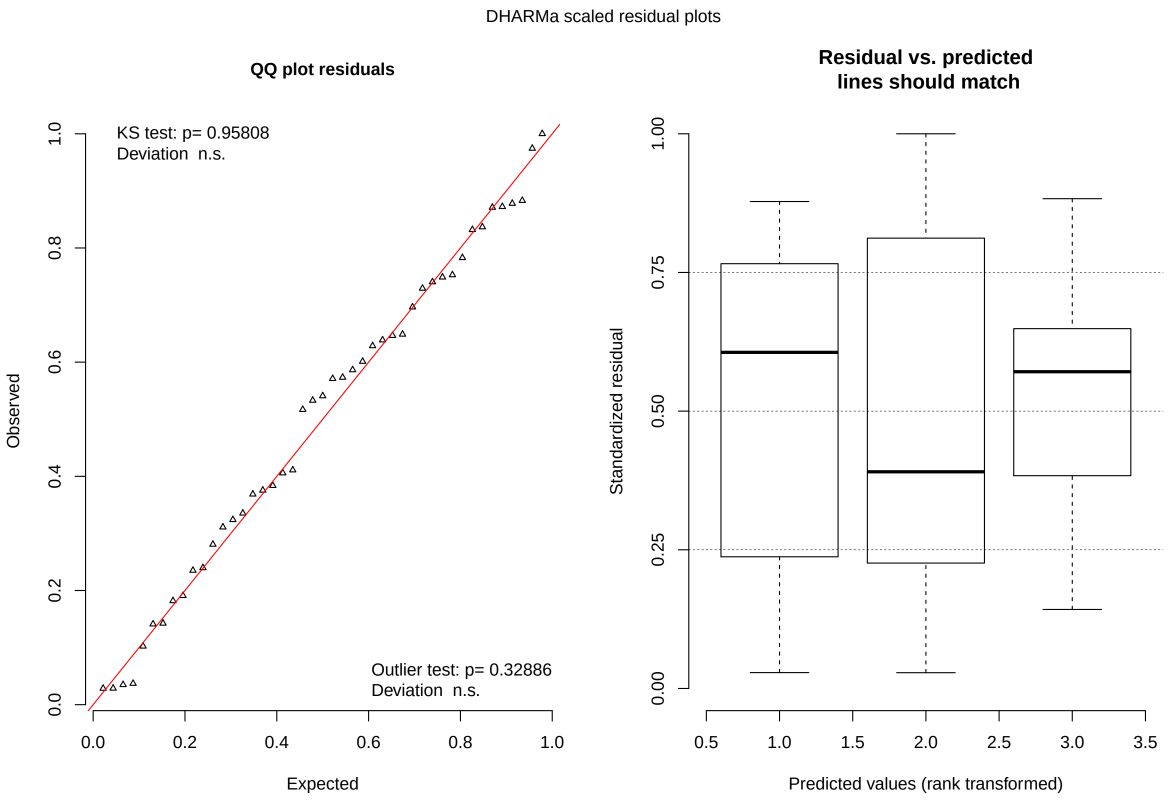
DHARMa scaled residual plots
QQ plot residuals
KS test: p= 0.95808
Deviation n.s.
Outlier test: p= 0.32886
Deviation n.s.
Observed
Expected
0.0
0.2
0.4
0.6
0.8
1.0
Residual vs. predicted
lines should match
Standardized residual
Predicted values (rank transformed)
0.00
0.25
0.50
0.75
1.00
0.5
1.0
1.5
2.0
2.5
3.0
3.5

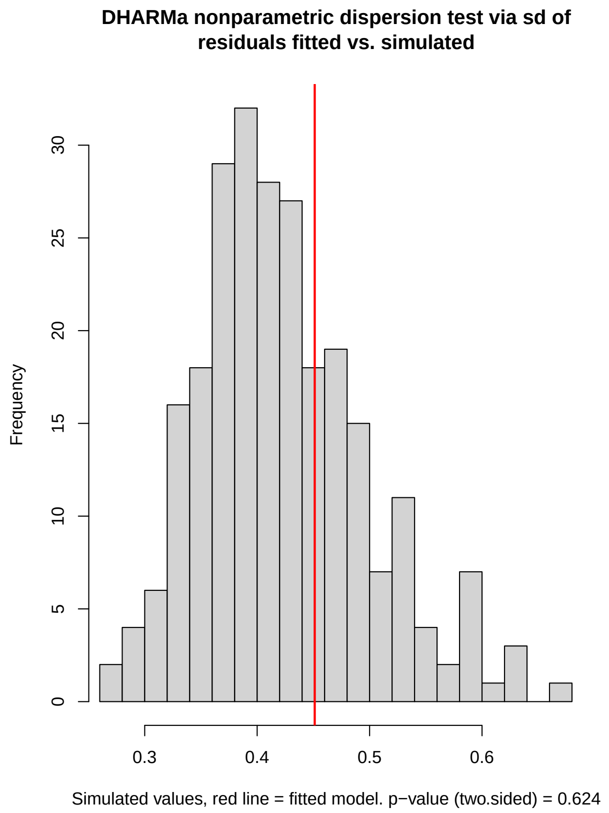

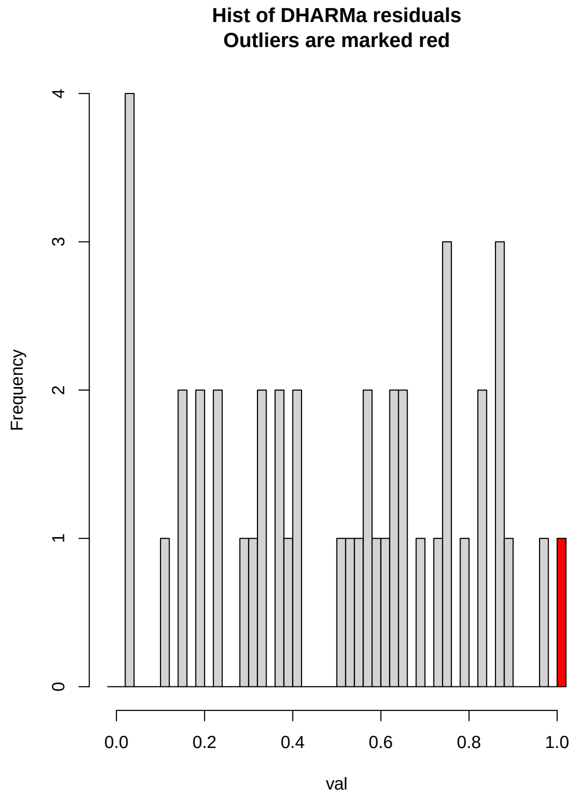


```
## $uniformity
##
##  One-sample Kolmogorov-Smirnov test
##
## data:  simulationOutput$scaledResiduals
## D = 0.07255, p-value = 0.9581
## alternative hypothesis: two-sided
##
##
## $dispersion
##
##  DHARMa nonparametric dispersion test via sd of residuals fitted vs.
##  simulated
##
## data:  simulationOutput
## ratioObsSim = 1.064, p-value = 0.624
## alternative hypothesis: two.sided
##
##
## $outliers
##
##  DHARMa outlier test based on exact binomial test
##
## data:  simulationOutput
## outLow = 0.0000000, outHigh = 1.0000000, nobs = 45.0000000, freqH0 =
## 0.0039841, p-value = 0.3289
## alternative hypothesis: two.sided
```

```
plot_predictors(exp2, m2totalres, "Solution")
```

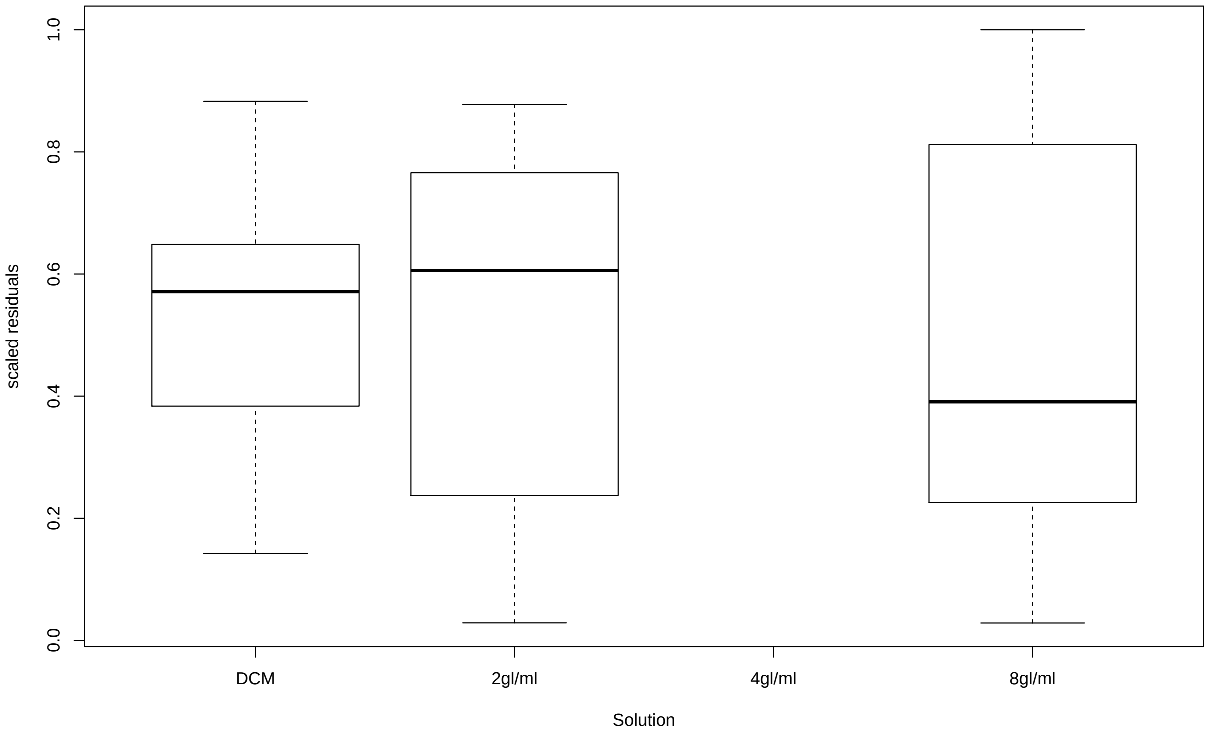

Results

```
Anova(m2totalsetup)
```

```
## Analysis of Deviance Table (Type II Wald chisquare tests)
##
## Response: log(Total.time.on.setup)
##           Chisq Df Pr(>Chisq)
## Solution 0.5236  2     0.7697
```

```
emmip(m2totalsetup, ~Solution, response=T, CIs = T, type="response")+
  ylab("Predicted total time on setup [sec]")+
  xlab("Solution")
```

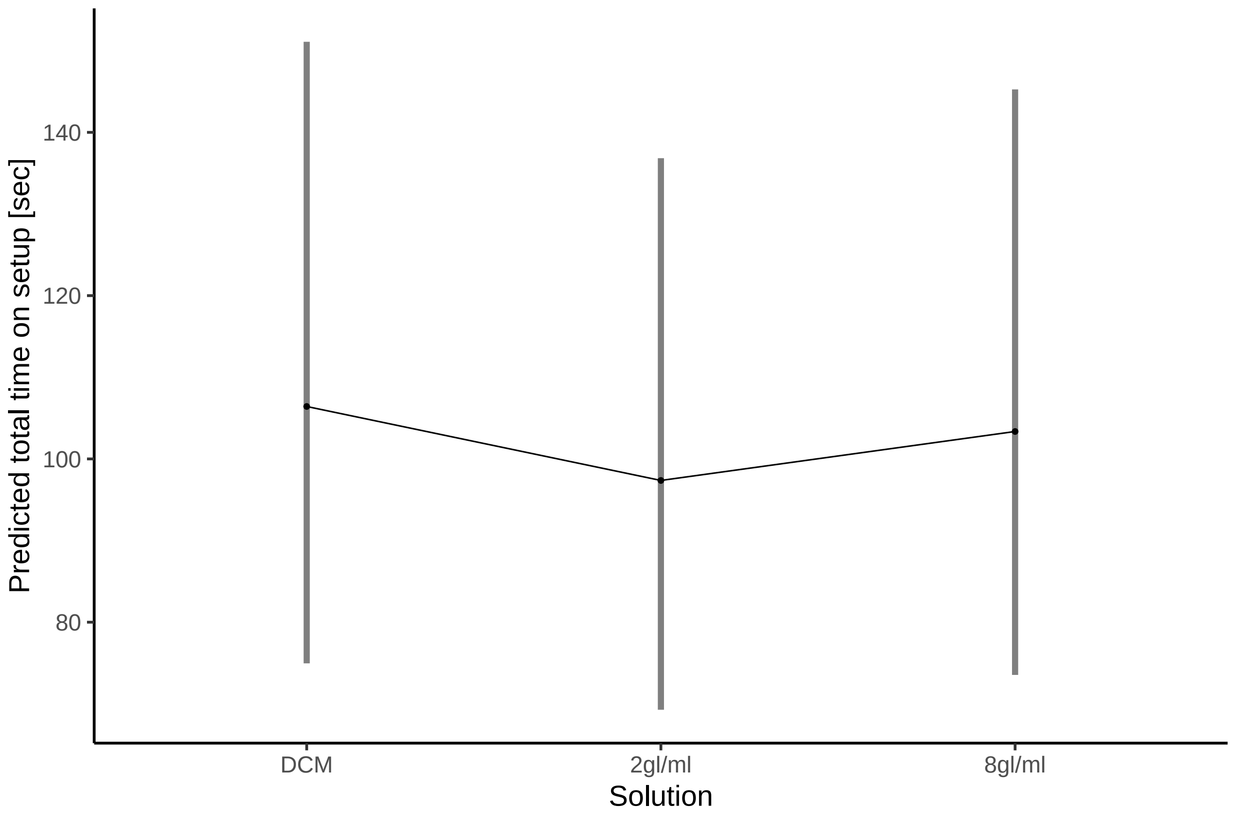

Panel figure

```
variables <-
  c("Duration.of.first.drinking", "Time.drinking", "Drinking.interruptions",
    "U.turns.to.nest", "Total.time.on.setup")
xlabs <- c("Duration first drinking [sec]", "Total time drinking [sec]",
           "Number of drinking interruptions", "U-turns to nest",
           "Total time on setup [sec]")

figs <- list()

for (plot in 1:length(variables)){
  figs[[plot]] <-
  ggplot(exp2, aes(x = Solution, y = .data[[variables[plot]]]))+
    geom_boxplot(outlier.size = 1) +
    scale_x_discrete(labels = c("DCM", "2gl/ml", "4gl/ml", "8gl/ml"))+
    ylab(paste0("\n", xlabs[plot]))+
    xlab(NULL)+
    theme_classic(8)
}

grid2<-
plot_grid(ncol = 2, labels = "AUTO",
          figs[[1]]+coord_cartesian(ylim = c(0,150)),
          figs[[2]]+coord_cartesian(ylim = c(0,150)),
          figs[[3]]+coord_cartesian(ylim = c(0,20)),
          figs[[4]]+coord_cartesian(ylim = c(0,3)),
```

```
          figs[[5]]+coord_cartesian(ylim = c(0,400)))
grid2
```

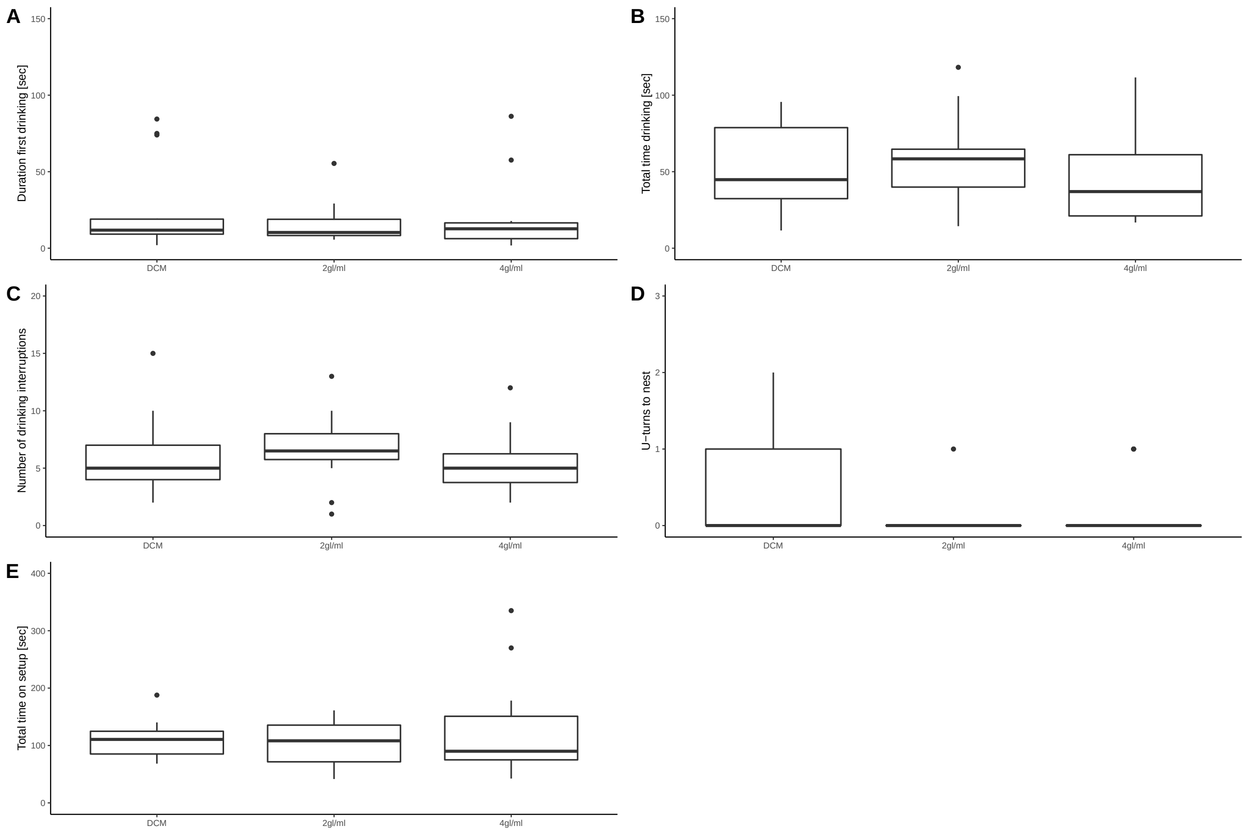

Experiment 3 - Food acceptance of sucrose-quinine solution

Load data

```
exp3<-read.xlsx("ESM2_raw_data.xlsx", sheetIndex = 4) %>%
  mutate(Solution = factor(Solution,
                           levels = c("DCM", "Pheromone"),
                           labels = c("DCM", "4gl/ml")),
         Quinine.dilution  = as.factor(Quinine.dilution)) %>%
  rename(`Quinine dilution` = Quinine.dilution)
```

Sample size

```
exp3 %>%
  group_by(`Quinine dilution`, Solution) %>%
  tally() %>%
  adorn_totals("row") %>%
  create_table()
```

Quinine dilution	Solution	n
8	DCM	96
8	4gl/ml	97
8.5	DCM	141

Quinine dilution	Solution	n
8.5	4gl/ml	136
Total	-	470

Dilution

In this experiment, we made a series dilution starting with 10 mM quinine in 0.5M sucrose and diluting it by adding same volume of 0.5M sucrose each step. Step 1 is 10mM. Each subsequent step has half the amount of quinine as the previous. What was our concentration?

```
levels(exp3$`Quinine dilution`)
```

```
## [1] "8"   "8.5"
```

```
seq = 2 #dilution 1 is 10mM quinine
start = 10
while (seq < 10) {
  start = start * .5
  print(paste0(start, "mM in dilution ", round(seq)))
  seq = seq +1
}
```

```
## [1] "5mM in dilution 2"
## [1] "2.5mM in dilution 3"
## [1] "1.25mM in dilution 4"
## [1] "0.625mM in dilution 5"
## [1] "0.3125mM in dilution 6"
## [1] "0.15625mM in dilution 7"
## [1] "0.078125mM in dilution 8"
## [1] "0.0390625mM in dilution 9"
```

So, dilution 8 corresponds to a 0.078125mM quinine solution. The 8.5 dilution corresponds to a $(0.078125 + 0.0390625)/2 = 0.0585938$mM quinine solution.

We now replace dilution with the concentration. To prevent too many digits, we convert to µM.

```
levels(exp3$`Quinine dilution`) <- c(paste0(round(0.078125 * 1000,1),"µM"),
                                     paste0(round(0.0585938 * 1000,1),"µM"))
```

Analysis

Food acceptance

```
exp3 %>%
  group_by(`Quinine dilution`, Solution) %>%
  summarise(percent_accepting_food = mean(Food.acceptance)*100,
            n = length(Food.acceptance),
            not_accepting = n - sum(Food.acceptance)) %>%
  create_table(column_names = c("Dilution", "Solution",
                                 "% accepting food", "Total ants",
                                 "Not accepting"),
               digits = 1)
```

Dilution	Solution	% accepting food	Total ants	Not accepting
78.1µM	DCM	16.7	96	80
78.1µM	4gl/ml	18.6	97	79

Dilution	Solution	% accepting food	Total ants	Not accepting
58.6µM	DCM	39.0	141	86
58.6µM	4gl/ml	44.9	136	75

```
m3<-glmmTMB(Food.acceptance ~ Solution * `Quinine dilution` + (1|colony),
            data = exp3,
            family = "binomial")
```

```
m3res<-simulateResiduals(m3)
checkmodel(m3res)
```

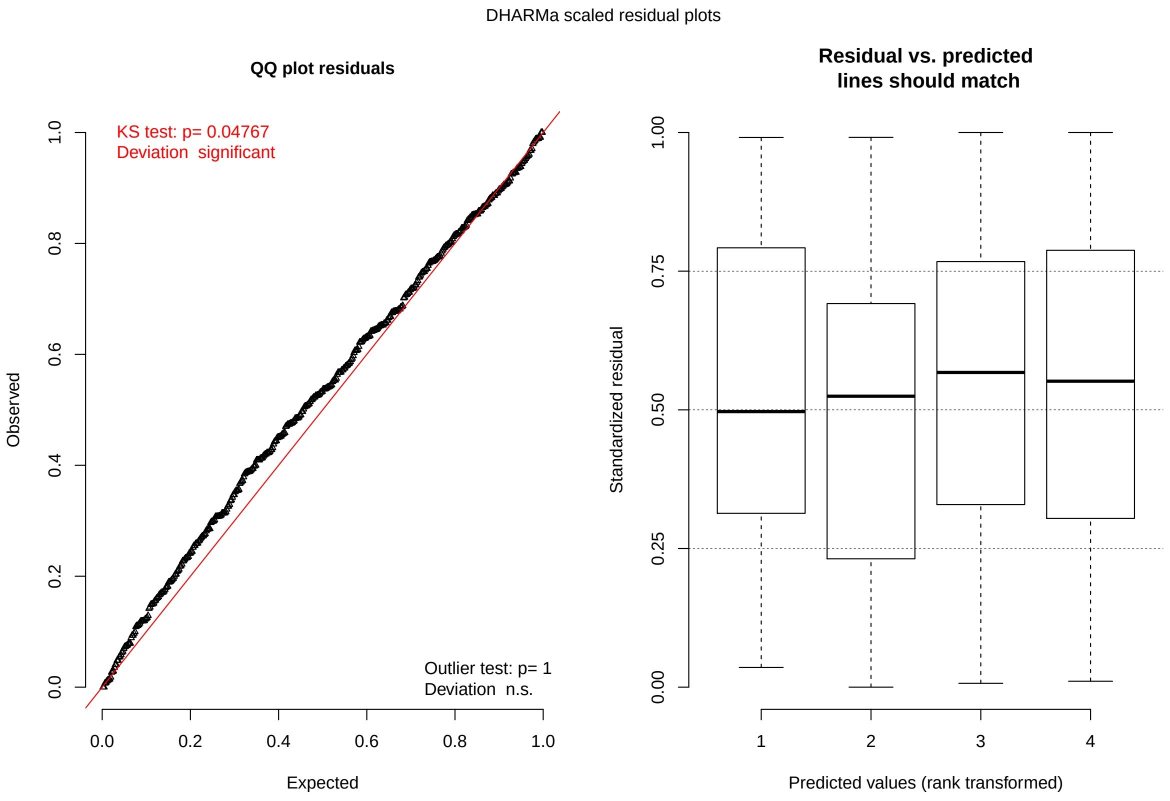

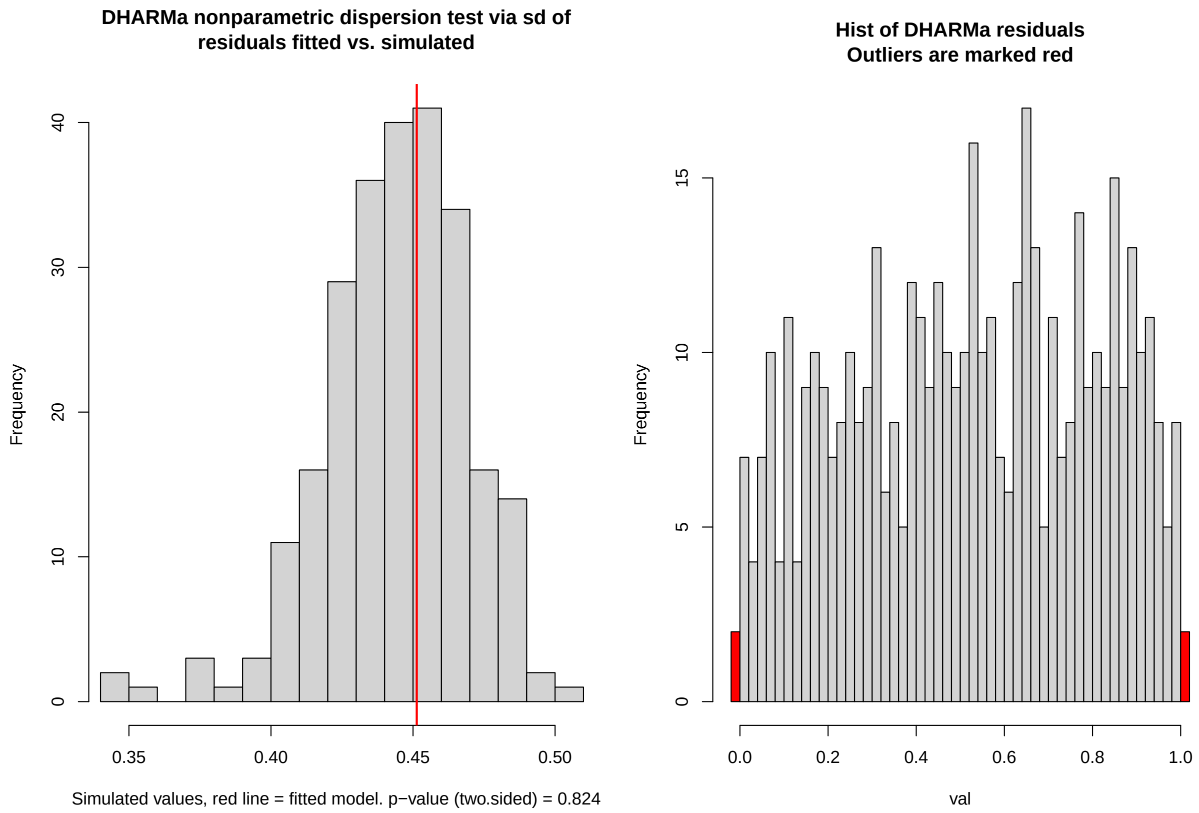


```
## $uniformity
##
##  One-sample Kolmogorov-Smirnov test
##
## data:  simulationOutput$scaledResiduals
## D = 0.063047, p-value = 0.04767
## alternative hypothesis: two-sided
##
##
## $dispersion
##
##  DHARMa nonparametric dispersion test via sd of residuals fitted vs.
##  simulated
##
## data:  simulationOutput
## ratioObsSim = 1.0167, p-value = 0.824
## alternative hypothesis: two.sided
##
##
## $outliers
##
##  DHARMa outlier test based on exact binomial test
##
## data:  simulationOutput
## outLow = 2.0000e+00, outHigh = 2.0000e+00, nobs = 4.7000e+02, freqH0 =
## 3.9841e-03, p-value = 1
## alternative hypothesis: two.sided
```

We have significant deviation. To account for that, we add a random variable ID, which identifies each row individually.

```
exp3$ID<-seq(1:nrow(exp3))
```

Now, we run the model again, with ID as random intercept.

```
m3<-glmmTMB(Food.acceptance ~ Solution * `Quinine dilution` + (1|colony) + (1|ID),
            data = exp3,
            family = "binomial")

m3res<-simulateResiduals(m3)
checkmodel(m3res)
```

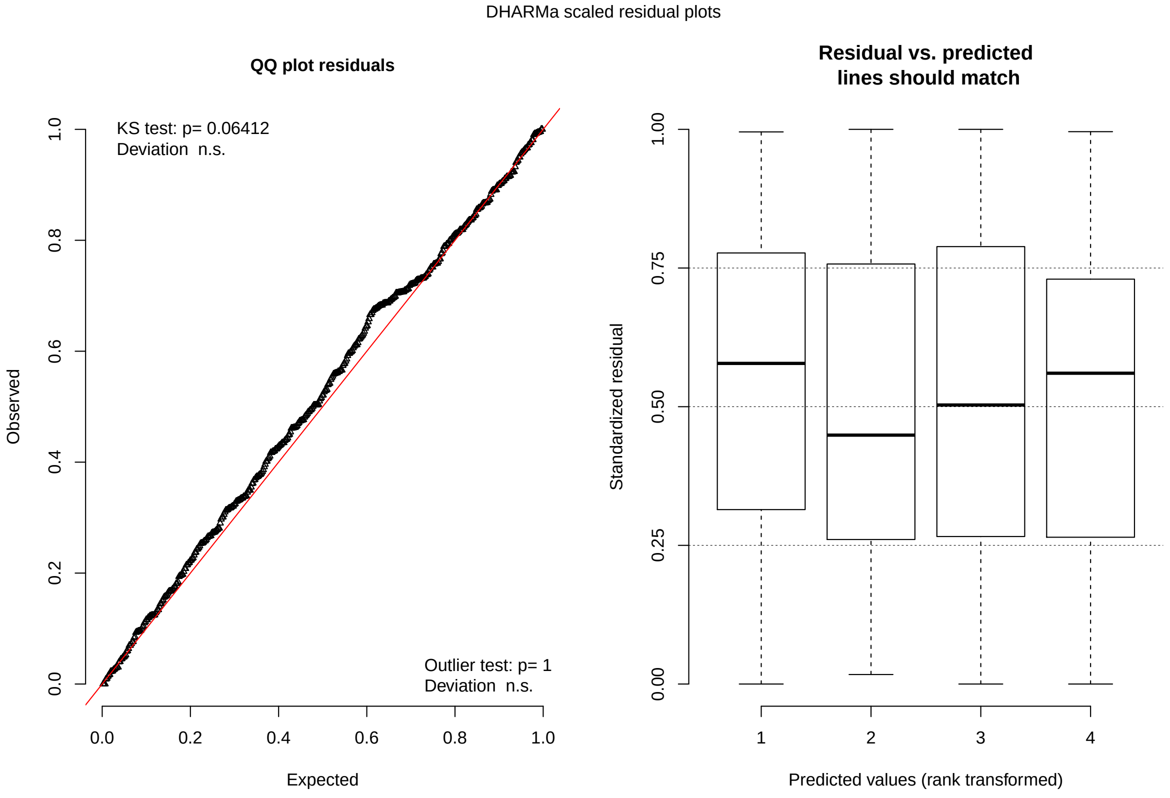

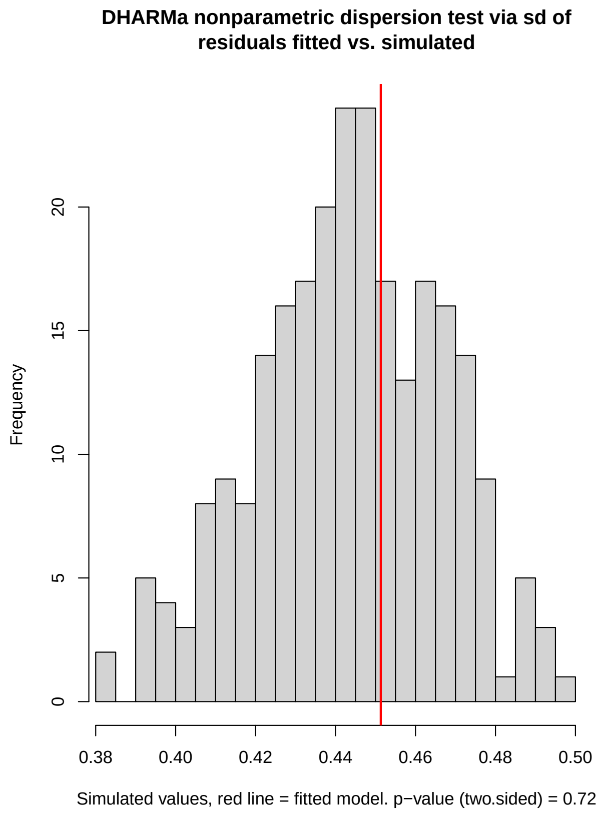

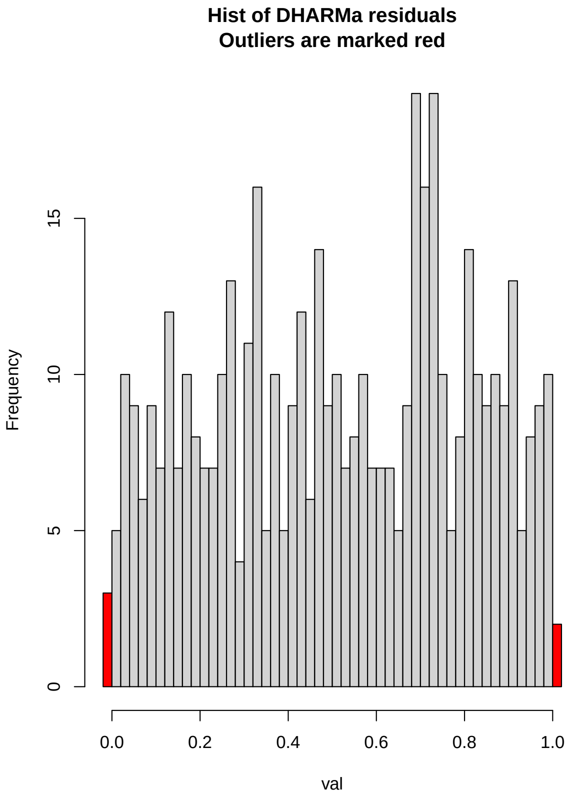


```
## $uniformity
##
##  One-sample Kolmogorov-Smirnov test
##
## data:  simulationOutput$scaledResiduals
## D = 0.060496, p-value = 0.06412
## alternative hypothesis: two-sided
##
##
## $dispersion
##
##  DHARMa nonparametric dispersion test via sd of residuals fitted vs.
##  simulated
##
## data:  simulationOutput
## ratioObsSim = 1.0177, p-value = 0.72
## alternative hypothesis: two.sided
##
##
## $outliers
##
##  DHARMa outlier test based on exact binomial test
##
## data:  simulationOutput
## outLow = 3.0000e+00, outHigh = 2.0000e+00, nobs = 4.7000e+02, freqH0 =
## 3.9841e-03, p-value = 1
## alternative hypothesis: two.sided
```

```
plot_predictors(exp3, m3res, predictors = c("Solution", "Quinine dilution"))
```

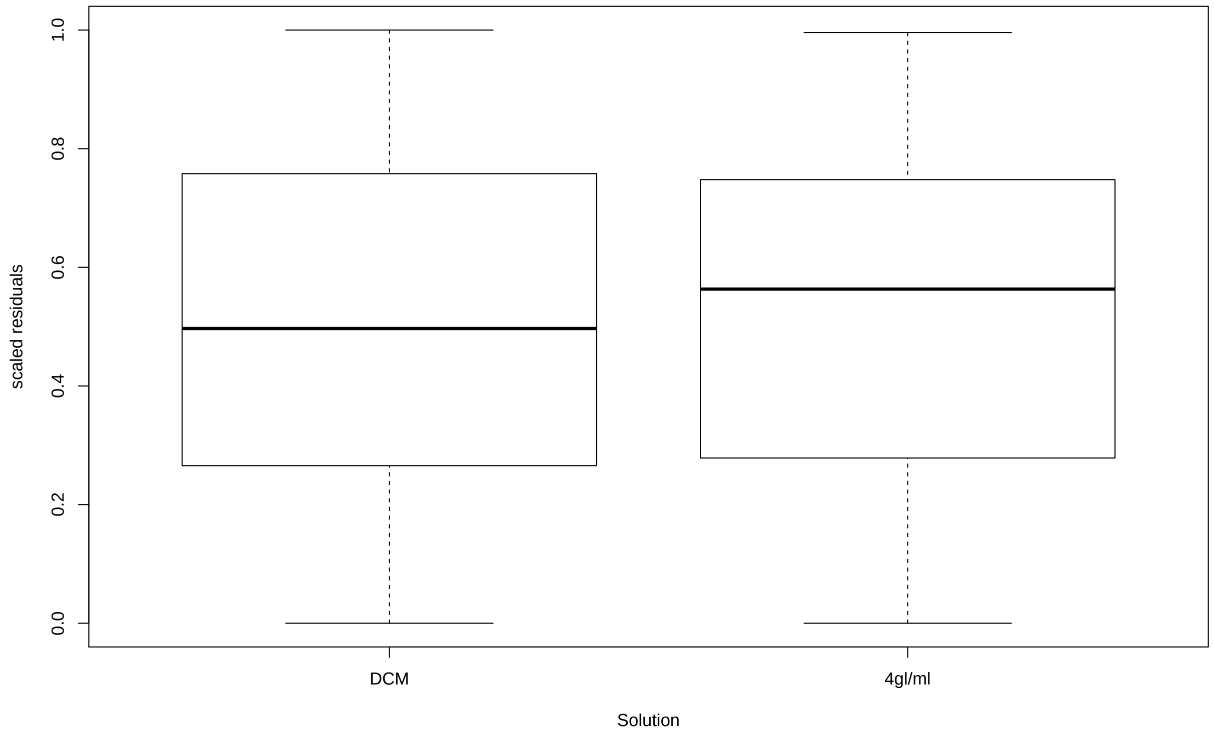

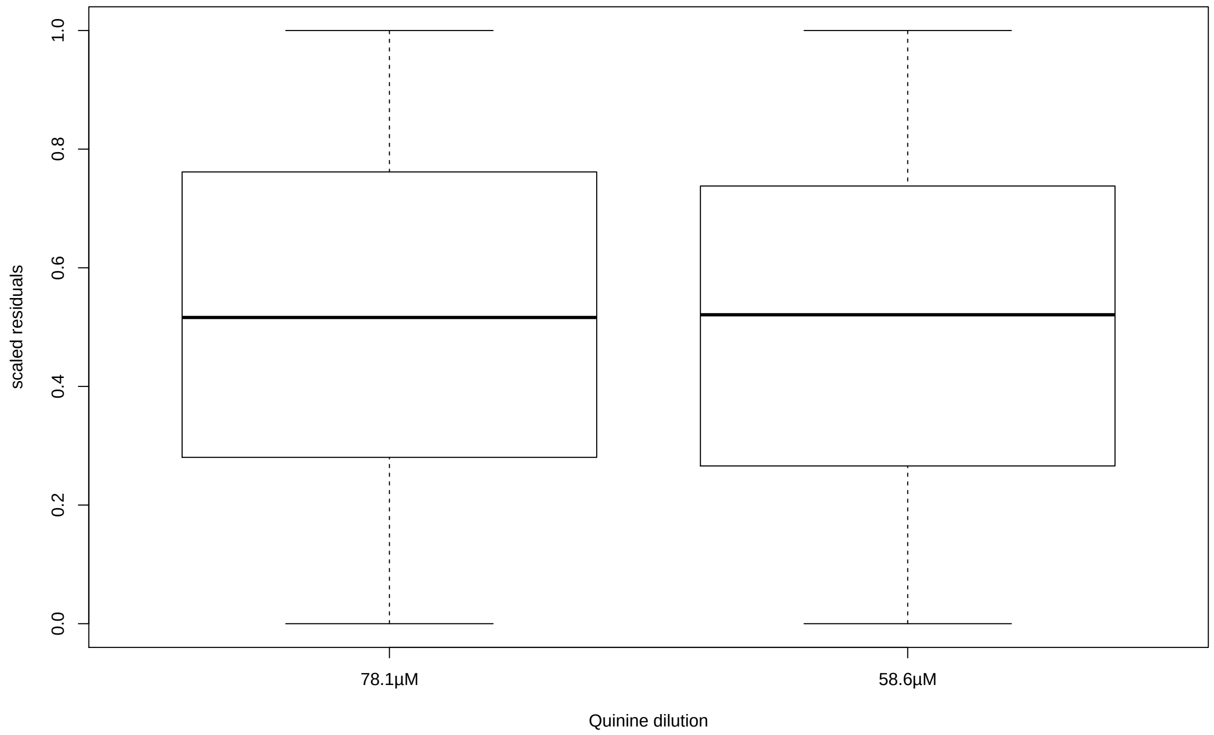

Better.

Results

```
Anova(m3)
```

```
## Analysis of Deviance Table (Type II Wald chisquare tests)
##
## Response: Food.acceptance
##                              Chisq Df Pr(>Chisq)
## Solution                    0.2964  1    0.58615
## `Quinine dilution`          9.8057  1    0.00174 **
## Solution:`Quinine dilution` 0.3352  1    0.56264
## ---
## Signif. codes:  0 '***' 0.001 '**' 0.01 '*' 0.05 '.' 0.1 ' ' 1
```

```
f<-
emmip(m3, `Quinine dilution` ~ Solution,
      CIs=T,
      type="response")+
  ylab("Predicted food acceptance")+
  xlab("Solution")+
  scale_color_manual(values = col)+
  coord_cartesian(ylim = c(0,1))
f
```

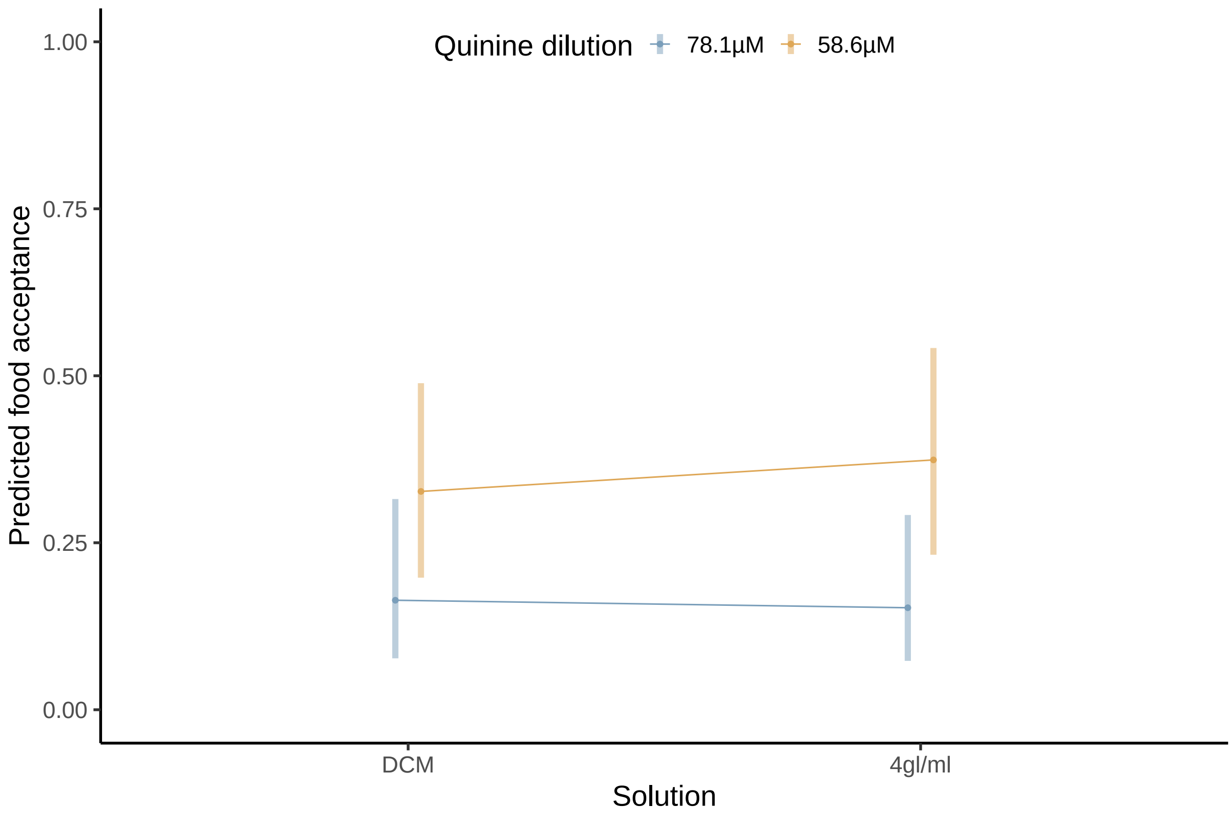

Figure

```
txtsize = 4
fig4<-
ggplot(exp3, aes(x=`Quinine dilution`,
                 y=Food.acceptance,
                 fill=Solution))+
  stat_summary(fun = mean, geom = "bar", color="black",
               position = "dodge")+
  stat_summary(fun.data = mean_cl_boot, geom = "errorbar", width = .1,
               position = position_dodge(.9))+
  scale_y_continuous(expand = c(0,0))+
  ylab("Quinine solution")+
  scale_x_discrete(limits = c("58.6µM", "78.1µM"))+
  scale_fill_manual(values = col)+
  coord_cartesian(ylim = c(0,1))+
  stat_summary(fun.y = function(x) x*0, fun.ymax = length,
               geom = "text", size=3,
               aes(label = paste0("n = ",..ymax..)),
               vjust=-1, position = position_dodge(width = .9))+
  ylab("Food acceptance")+
  annotate(geom = "text", x = 1.5, y = .72, label = "**", size = txtsize)+
  annotate(geom = "segment", x = 1, xend = 2, y = .7, yend = .7)+
  annotate(geom = "text", x = 1, y = .62, label = "n.s.", size = txtsize)+
  annotate(geom = "segment", x = .8, xend = 1.2, y = .6, yend = .6)+
  annotate(geom = "text", x = 2, y = .62, label = "n.s.", size = txtsize)+
  annotate(geom = "segment", x = 1.8, xend = 2.2, y = .6, yend = .6)
```

```r
fig4
```

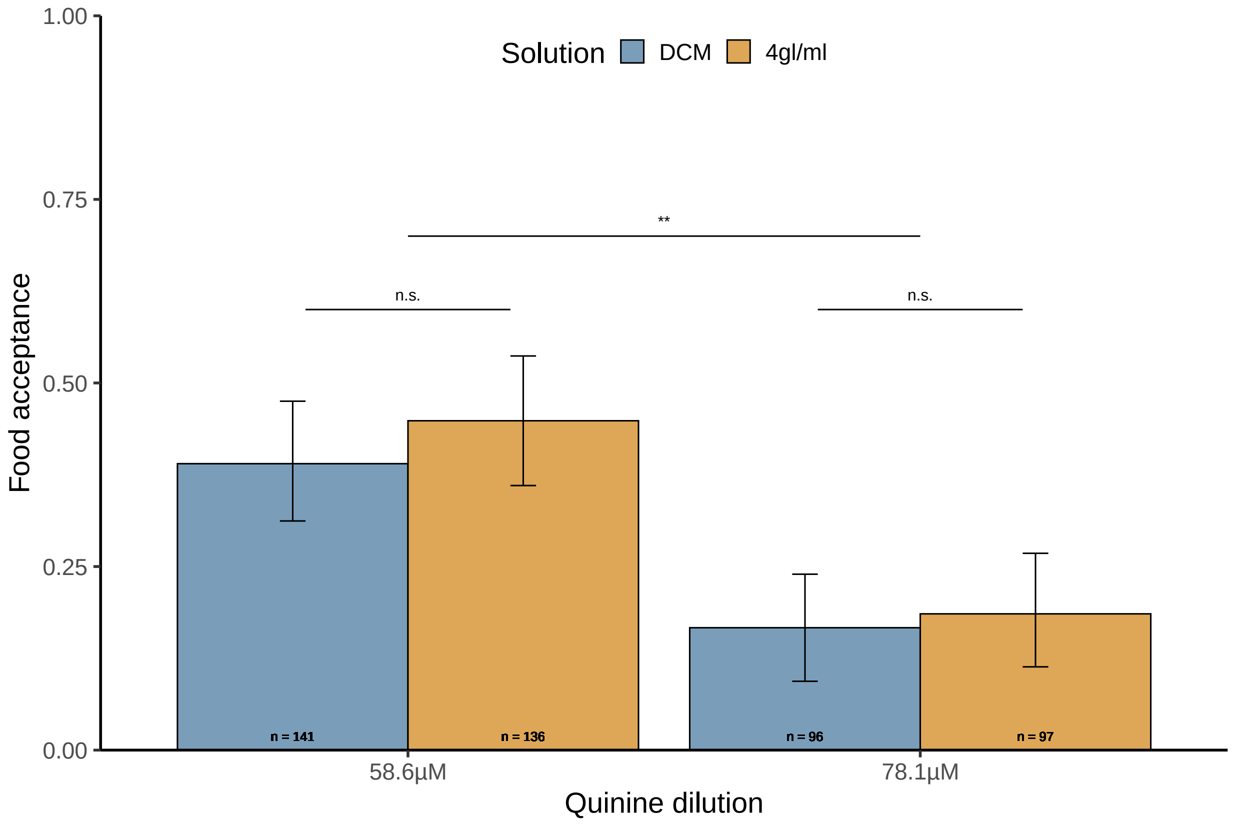

Package information

Package versions

```r
for (package in lib) {print(paste(package,"=", packageVersion(package)))}
```

```
## [1] "xlsx = 0.6.3"
## [1] "ggplot2 = 3.3.0"
## [1] "cowplot = 1.0.0"
## [1] "knitr = 1.28"
## [1] "DHARMa = 0.2.7"
## [1] "glmmTMB = 1.0.1"
## [1] "car = 3.0.7"
## [1] "emmeans = 1.4.5"
## [1] "dplyr = 0.8.5"
## [1] "janitor = 1.2.1"
```

Package references

We thank all package authors for their efforts in creating and maintaining the packages.

Brooks, Mollie E., Kasper Kristensen, Koen J. van Benthem, Arni Magnusson, Casper W. Berg, Anders Nielsen, Hans J. Skaug, Martin Maechler, and Benjamin M. Bolker. 2017. "glmmTMB Balances Speed and

Flexibility Among Packages for Zero-Inflated Generalized Linear Mixed Modeling.” *The R Journal* 9 (2): 378–400. https://journal.r-project.org/archive/2017/RJ-2017-066/index.html.

Dragulescu, Adrian, and Cole Arendt. 2020. *Xlsx: Read, Write, Format Excel 2007 and Excel 97/2000/Xp/2003 Files*. https://CRAN.R-project.org/package=xlsx.

Firke, Sam. 2020. *Janitor: Simple Tools for Examining and Cleaning Dirty Data*. https://CRAN.R-project.org/package=janitor.

Fox, John, and Sanford Weisberg. 2019. *An R Companion to Applied Regression*. Third. Thousand Oaks CA: Sage. https://socialsciences.mcmaster.ca/jfox/Books/Companion/.

Hartig, Florian. 2020. *DHARMa: Residual Diagnostics for Hierarchical (Multi-Level / Mixed) Regression Models*. https://CRAN.R-project.org/package=DHARMa.

Lenth, Russell. 2020. *Emmeans: Estimated Marginal Means, Aka Least-Squares Means*. https://CRAN.R-project.org/package=emmeans.

R Core Team. 2020. *R: A Language and Environment for Statistical Computing*. Vienna, Austria: R Foundation for Statistical Computing. https://www.R-project.org/.

Wickham, Hadley. 2016. *Ggplot2: Elegant Graphics for Data Analysis*. Springer-Verlag New York. https://ggplot2.tidyverse.org.

Wickham, Hadley, Romain François, Lionel Henry, and Kirill Müller. 2020. *Dplyr: A Grammar of Data Manipulation*. https://CRAN.R-project.org/package=dplyr.

Wilke, Claus O. 2019. *Cowplot: Streamlined Plot Theme and Plot Annotations for 'Ggplot2'*. https://CRAN.R-project.org/package=cowplot.

Xie, Yihui. 2014. “Knitr: A Comprehensive Tool for Reproducible Research in R.” In *Implementing Reproducible Computational Research*, edited by Victoria Stodden, Friedrich Leisch, and Roger D. Peng. Chapman; Hall/CRC. http://www.crcpress.com/product/isbn/9781466561595.

———. 2015. *Dynamic Documents with R and Knitr*. 2nd ed. Boca Raton, Florida: Chapman; Hall/CRC. https://yihui.org/knitr/.

———. 2020. *Knitr: A General-Purpose Package for Dynamic Report Generation in R*. https://yihui.org/knitr/.
